# DNA damage and transcription stress cause ATP-mediated redesign of metabolism and potentiation of anti-oxidant buffering

**DOI:** 10.1038/s41467-019-12640-5

**Published:** 2019-10-25

**Authors:** Chiara Milanese, Cíntia R. Bombardieri, Sara Sepe, Sander Barnhoorn, César Payán-Goméz, Donatella Caruso, Matteo Audano, Silvia Pedretti, Wilbert P. Vermeij, Renata M. C. Brandt, Akos Gyenis, Mirjam M. Wamelink, Annelieke S. de Wit, Roel C. Janssens, René Leen, André B. P. van Kuilenburg, Nico Mitro, Jan H. J. Hoeijmakers, Pier G. Mastroberardino

**Affiliations:** 1 Department of Molecular Genetics, Erasmus University Medical Center, Rotterdam, the Netherlands; 2 Facultad de Ciencias Naturales y Matemáticas, Universidad del Rosario, Bogotá, Colombia; 3 Department of Pharmacological and Biomolecular Sciences, University of Milan, Milan, Italy; 4 Princess Máxima Center for Pediatric Oncology, Utrecht, The Netherlands; 5 Cologne Excellence Cluster for Cellular Stress Responses in Ageing-Associated Diseases (CECAD) and Systems Biology of Ageing Cologne, University of Cologne, Cologne, Germany; 6 Department of Clinical Chemistry, VU University Medical Center, Amsterdam, the Netherlands; 7 Laboratory of Genetic Metabolic Diseases, Academic Medical Center, Amsterdam, the Netherlands; 8 Oncode Institute, Princess Máxima Center, Utrecht, Netherlands; 9 Department of Life, Health and Environmental Sciences, University of L’Aquila, L’Aquila, Italy

**Keywords:** Metabolomics, Nucleotide excision repair

## Abstract

Accumulation of DNA lesions causing transcription stress is associated with natural and accelerated aging and culminates with profound metabolic alterations. Our understanding of the mechanisms governing metabolic redesign upon genomic instability, however, is highly rudimentary. Using *Ercc1*-defective mice and *Xpg* knock-out mice, we demonstrate that combined defects in transcription-coupled DNA repair (TCR) and in nucleotide excision repair (NER) directly affect bioenergetics due to declined transcription, leading to increased ATP levels. This in turn inhibits glycolysis allosterically and favors glucose rerouting through the pentose phosphate shunt, eventually enhancing production of NADPH-reducing equivalents. In NER/TCR-defective mutants, augmented NADPH is not counterbalanced by increased production of pro-oxidants and thus pentose phosphate potentiation culminates in an over-reduced redox state. Skin fibroblasts from the TCR disease Cockayne syndrome confirm results in animal models. Overall, these findings unravel a mechanism connecting DNA damage and transcriptional stress to metabolic redesign and protective antioxidant defenses.

## Abstract (web summary)

ERCC1 is involved in a number of DNA repair pathways including nucleotide excision repair. Here the authors showed that reduced transcription in *Ercc1*-deficient mouse livers and cells increases ATP levels, suppressing glycolysis and rerouting glucose into the pentose phosphate shunt that generates reductive stress.

## Introduction

Information encoded by DNA is at the hierarchical apex of virtually all biological systems and its accurate preservation is therefore essential for life. DNA, however, is constantly exposed to exogenous and endogenous physicochemical threats that can modify its nucleotides. Unless promptly resolved, these alterations corrupt genome fidelity and compromise normal physiology promoting either cancer or aging and its related diseases^1^. The cell has therefore developed an intricate network of mechanisms designated to maintain genome integrity, which comprises pathways specialized in correcting distinctive classes of DNA lesions.

Nucleotide Excision Repair (NER) eliminates base pair-disrupting lesions. It involves more than 30 genes and progresses along two converging branches; global genome NER (GG-NER) operates genome-wide, while transcription-coupled repair (TCR) removes only lesions blocking elongating RNA polymerase II (RNAPII) to rescue transcription^2,3^. Defects in GG-NER therefore lead to the accumulation of base pair-disrupting lesions in the whole genome, with the notable exception of transcribed regions, in which DNA damage is removed by still functional TCR. Correction of the transcribed strand in GG-NER defective organisms allows cell survival even in the presence of high DNA damage load. Upon replication, however, accumulated lesions are bypassed by trans-lesion polymerases, which are more error-prone and thus increase the onset of mutations and hence cancer. On the other hand, TCR deficiencies cause transcription arrest because of unrepaired damage, which in turn induces premature cell death and expression defects accelerating aging. These effects are particularly pronounced in those organs and tissues that strongly depend on TCR, for instance post-mitotic tissues, which cannot dilute accumulating DNA damage by DNA replication (segmental progeria)^1,3^. When GG-NER and TCR defects are combined–as in the case of mutations in the multi-functional NER protein ERCC1, which also causes deficiencies in cross-link repair–the aging process is further accelerated and extended to additional organs and tissues. Consistently, in mouse models, expression of only a truncated *Ercc1* allele (*Ercc1*^Δ/−^) causes impaired NER and cross-link repair, increased DNA damage, and a wide-spread progeroid phenotype that includes prominent liver, kidney, and neuronal aging^4,5^. Overall, *Ercc1*^Δ/−^ mutants exhibit an exceptionally wide spectrum of natural aging features in a strongly accelerated fashion, recapitulate the corresponding human progeroid XFE-1 syndrome, and prove a causative nexus between DNA damage accumulation and aging-associated multi-morbidity^1,6^.

DNA damage accumulation instigates a cellular response aimed at attenuating growth and activating protective mechanisms. Current knowledge of this adaptive, or ‘survival’ response mainly derives from gene expression experiments in repair-deficient models^4,7^ indicating that metabolic redesign is central to adaptation and suggesting that glucose catabolism is suppressed in DNA repair deficient mice. Gene expression studies have also shown parallel up-regulation of genes involved in oxido-reductive (redox) homeostasis, consistently with a key role of reactive species (RS) in generating endogenous DNA damage^8^.

Gene expression, however, remains an insufficient indicator of enzymes’ levels and activities, and of metabolic fluxes, which are extensively regulated by allosteric mechanisms^9^. High concentrations of global modulators–e.g. ATP, the main energy carrier–favor their interaction with target enzymes to induce structural rearrangements that cause functional (de)activation and therefore feedback loop control^9^. Allosteric regulation allows robust and swift modulation of the metabolic fluxes and therefore critically contributes to the acute response to stress. On these premises, the important elements emerged from previous studies based on gene expression analysis in DNA repair deficient mice are necessarily incomplete and need to be complemented by biochemical analyses to resolve the mechanisms coupling metabolic reprogramming to DNA damage accumulation and the adaptive response.

An eventual role of ATP levels, and therefore of the cellular energetic status, and of the connected allosteric modulation of metabolism in response to DNA damage has never been described. Yet, the following biochemical concepts suggest that ATP-mediated regulation might, at least in principle, act on and respond to DNA damage accumulation to contribute to the adaptive response: from the energetic standpoint, macromolecular synthesis is a highly demanding process^10,11^ and it is therefore conceivable that reduced transcription and consequent translational levels may impact the cellular bioenergetics balance with direct consequences on metabolism. Hence it is possible that genomic instability and continued transcription-arrest affect the cellular bioenergetics balance and metabolism contributing to activation of the adaptive response.

In this work we describe a molecular process coupling detection of transcription stalling to metabolic rearrangements operated by ATP-mediated allosteric mechanisms to potentiate cellular defenses to stress. Overall, we provide evidence for a mechanism governing the adaptive response elicited by sustained block of transcription caused by defective DNA repair.

## Results

### Defective NER causes progressive decline of transcription

**Fig. 1:**
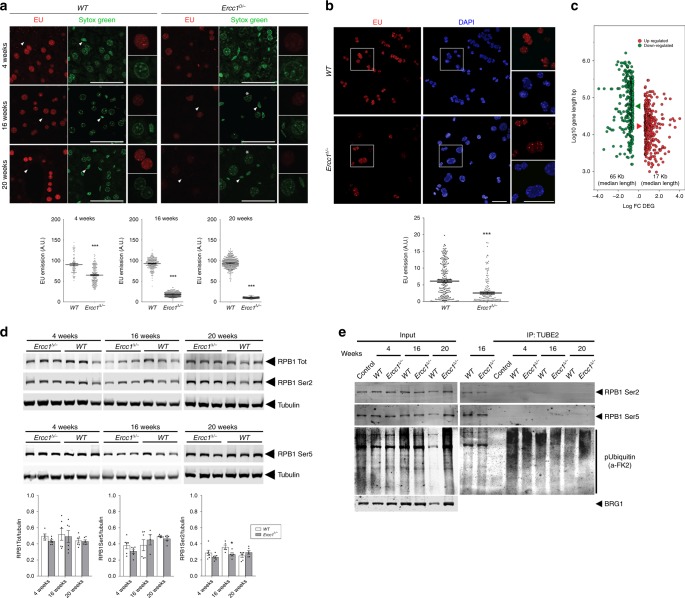
Defective NER causes transcription block. **a** Incorporation of the uridine analog EU into nascent RNA in vivo is reduced in *Ercc1*^*∆/*−^ mouse livers and further decreases with age. EU was injected in the peritoneum of 4, 16, and 20-week old animals; tissues were harvested 5 h after injection. EU incorporation occurs predominantly in hepatocytes compared to transcriptionally less active epithelial cells (arrowheads); Sytox green nucleic acid staining is detectable in both cell types. Polyploid nuclei (asterisk), which are a hallmark of natural liver aging, are already detectable in 16 and 20-week old *Ercc1*^*∆/*−^ mutants; *n* ≥ 80 cells from 3 mice per group. **b** Decreased EU incorporation (3 mM, 2 h) in freshly isolated primary hepatocytes from adult mice (16-week old, *n* = 3). Scatter dot plot represents the EU intensity distribution of biological replicates. **c** Graphical representation of the correlation between expression levels (DEG - differentially expressed gene; FC- fold-change) and gene length in 16-week old *Ercc1*^∆/−^ versus wild-type livers. In *Ercc1*^∆/−^, 356 genes were up-regulated and 382 down-regulated; the median length of up-regulated genes is 17371 bp; (red arrowhead), while that of down-regulated ones is 65010 bp (green arrowhead). Mann–Whitney test (*p* < 0.0001) indicates that gene length distribution between up- and down-regulated genes is statistically significant. Transcription decline in *Ercc1*^*∆/−*^ is more pronounced in longer genes. Each point represents an individual gene. Gene length is in log10 scale, while gene expression level is in log2. **d** Representative western blot of RNA pol II (RPB1) expression and phosphorylation levels of Ser2 (elongating RNA pol II) and Ser5 (initiating RNA pol II) residues in 4, 16 and 20 week-old mice (*n* ≥ 3 per group, top panel). Bar graphs from 2 independent technical replicates (lower panel). **e** Ubiquitinated RNA pol II is undetectable in both *Ercc1*^*∆/−*^ and control livers. Liver lysates were incubated with Ubiquitin Affinity Matrix (TUBE2); both unbound (input) and eluted (bound) fractions were resolved by SDS/PAGE and probed with antibodies against Ser2- and Ser5-phosphorylated RNA Pol II and Ubiquitin. Error bars indicate mean ± s.e.m. *p < 0.05; ***p < 0.001; Student’s *t*-test. Scale bar: 50 μm. Original data and images are provided as a Source Data file

TCR amends DNA lesions that arrest RNAPII elongation and defective repair in *Ercc1*^Δ/−^ mice–which feature severe repair defects that also include TCR–might predictably compromise transcription. To test this hypothesis in vivo, we determined global transcription levels in mice at different ages by measuring incorporation of intraperitoneally injected 5-ethynyluridine (EU), a uridine analog detectable with fluorescent azides using a copper (I)-catalyzed cycloaddition reaction (“click” chemistry)^12^. EU incorporation, reflecting overall transcriptional output, was significantly and progressively reduced in *Ercc1*^Δ/−^ livers (Fig. 1a); it transitioned from ~70% of matched controls at 4 weeks–when polyploidization, a distinctive hepatic sign of natural aging that is also associated with of *Ercc1* deficiency^13–15^, is already apparent–to ~20% at 16 weeks, to finally reach a remarkably low level of 10% at 20 weeks. At this age, many *Ercc1*^Δ/−^ animals are moribund and some have already succumbed. Transcription reduction in *Ercc1*^Δ/−^ was confirmed in freshly isolated hepatocytes and in organotypic liver slices from adult mice (age 16 weeks) (Fig. 1b), ruling out the possibility that the difference in signal was due to different EU penetration or uptake into livers of control and experimental groups. Because NER also repairs lesions in the ribosomal genes^16^, we measured EU intensities in the nucleolus versus the rest of the nucleus to examine whether RNA Pol I, RNA Pol II, or both are involved in this transcriptional collapse. We found that reduction of EU signal in mutant animals is comparable in the two sub-compartments (Supplementary Fig. 1), consistently with a genome-wide operating process. These studies also disclosed striking heterogeneity in levels of nascent RNA synthesis among hepatocytes of *Ercc1*^Δ/−^ mice, in sharp contrast with the observations in wild-type controls: while in mutant livers some cells displayed levels of RNA synthesis comparable to wild-type animals–thus indicating that EU-incorporation *per se* is unaffected–other neighboring hepatocytes exhibited very low RNA synthesis. Moreover, EU incorporation in vivo indicates that in *Ercc1*^Δ/−^ heterogeneity changes in time and is more pronounced in young animals. Such high and time-dependent variability is consistent with a stochastic pattern in the underlying cause, such as that expected from DNA damage accumulation.

Transcription decline was independently substantiated by determining the expression of long and short genes as inferred from liver transcriptomic data^17^. Because DNA damage is stochastic, the likelihood that a gene suffers a lesion is directly proportional to its length and thus accumulation of DNA damage is expected to cause preferential depletion in longer transcripts^18,19^. Indeed, the expression profile of *Ercc1*^Δ/−^ livers (16-weeks old) was relatively enriched in transcripts from short genes and significantly depleted in transcripts of long genes (Fig. 1c, Supplementary Table 1).

We investigated whether strong overall transcription decline was attributable to reduced RNA polymerase II (RPB1) and therefore evaluated its expression at mRNA and protein level, as well as its phosphorylation status in *Ercc1*^Δ/−^ mice of different ages. Immunoblot analysis did not reveal a drop in RNA pol II largest subunit, RPB1 (Fig.1d–RPB1 tot). Analysis of mRNA levels also failed to indicate a down-regulation of RNA pol II subunits (Supplementary Table 2). To gather further insights on transcription, we investigated RNA pol II phosphorylation on Ser2 and Ser5 in its C-terminal repeat domain, which respectively reflect transcription elongation and initiation^20^. Immunoblot analysis did not detect a decline in time in Ser2 or Ser5 phosphorylation in *Ercc1* liver extracts; in fact, there is a even moderate increase in Ser5 phosphorylation (see Fig. 1d). We also explored whether transcription decline in *Ercc1*^Δ/−^ is associated with increased ubiquitination of RNA Pol II, which–despite involvement of non-conventional signaling functions of ubiquitinylaiton in genome integrity maintenance^21^–has been shown to target the enzyme to degradation as a consequence of DNA damage^22,23^. Immunoblot analysis following immunocapture of ubiquitinated proteins, however, failed to reveal any signal for RNA Pol II (Fig. 1e), in agreement with our observation that total RNA pol II levels in *Ercc1*^Δ/−^ livers were unaffected. General ubiquitination increased only in 20-week old *Ercc1*^Δ/−^ mice (Supplementary Fig. 2). Collectively these data clearly rule out a decrease of RNA Pol II in *Ercc1*^Δ/−^ mice that can explain the dramatic decline in transcriptional output. The finding that the level of elongating, Ser2-phosphorylated RNA Pol II at 16 and 20 weeks is unaffected while transcriptional output is collapsing (Fig. 1a) suggests that these elongating RNA Pol II molecules are non-productive, in line with the concept that they may be stalled because of accumulating, unrepaired DNA lesions.

### Decreased transcription leads to elevated ATP pool

**Fig. 2:**
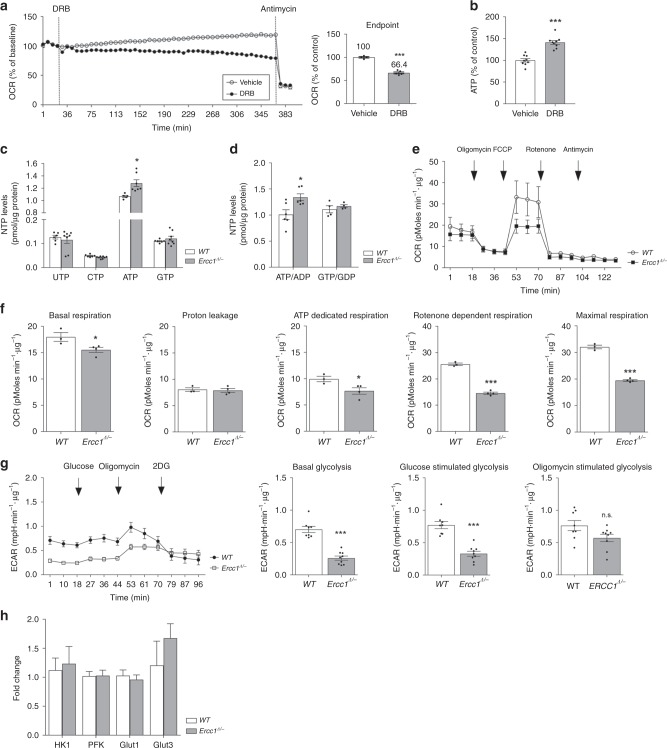
Diminished transcription leads to increased intracellular ATP levels. **a** Mitochondrial respiration–measured in real time as oxygen consumption rate (OCR)–is reduced in hepatocytes after the injection of the chemical transcription inhibitor DRB, thus indicating the bioenergetics burden of macromolecular synthesis (left trace). After 6 h of DRB treatment, transcription is reduced to 66.4% of its original level (right panel). **b** 6-h incubation with DRB leads to increased intracellular ATP levels, which confirm the bioenergetics burden of transcription; *n* ≥ 4, data were collected from 2 independent technical experiments. **c**, **d** HPLC analysis indicates that defective NER in *Ercc1*^*Δ/−*^ (16 weeks) is associated with increased ATP levels and ATP/ADP ratio in vivo (16 week-old animals); *n* ≥ 4, data were obtained from 2 independent technical experiments. **e**, **f** Measure of respiration in primary hepatocytes shows that increased ATP levels in *Ercc1*^*Δ/−*^ are not the consequence of augmented mitochondrial respiration. OCR was measured in freshly extracted hepatocytes in basal conditions and after sequential injections of the following molecules modulating mitochondrial activity: oligomycin, FCCP, rotenone and antimycin-A (see methods for details). Basal mitochondrial respiration and proton leakage are unaltered, while ATP-dedicated respiration, maximum respiratory capacity and rotenone-sensitive respiration are decreased in mutants. Cells were extracted from *n* ≥ 3 mice per group, data were collected from 2 independent technical replicates. **g** Increased ATP levels in *Ercc1*^*Δ/−*^ are associated with glycolysis inhibition. Basal- and glucose-stimulated glycolysis, measured as extracellular acidification rate (ECAR), is decreased in *Ercc1*^*Δ/−*^ mutants; oligomycin-stimulated glycolytic capacity, however, is retained and comparable to *WT*. Left, Seahorse Extracellular Flux Analyzer trace; right, bar graph of basal and glucose- and oligomycin-stimulated ECAR. Cells were extracted from *n* = 3 16-week old mice, 3 technical replicates. **h** Quantitative PCR indicates that the observed effects do not depend on expression changes in rate-limiting glycolytic enzymes or in glucose transporters (*n* ≥ 4 mice per group, 16 weeks). Error bars indicate mean ± s.e.m. *p < 0.05, **p < 0.01, ***p < 0.001; Student *t*-test. Original data are provided as a Source Data file

Transcription is a high energy-demanding process and reduced transcription likely also diminishes translation, which is even more energy-consuming. Hence reduced transcription may impact the overall cellular energy status. We initially probed energy parameters by monitoring the activity of the major cellular ATP generator, mitochondria, in cells where transcription was chemically blocked and found that 6 h treatment with 5,6-dichloro-1-beta-D-ribofuranosylbenzimidazole (DRB) suppresses mitochondrial oxygen consumption in both transformed hepatocytes (Fig. 2a) and primary fibroblast cultures (Supplementary Fig. 3A). This is a general effect, genuinely attributable to transcription block, because it could be reproduced by other inhibitors acting through different mechanisms: alpha-amanitin and actinomycin D (Supplementary Fig. 3A). A more direct link between transcription stalling and bioenergetics emerges from the analysis of ATP levels, which–as expected–are increased in cells exposed to transcription inhibitors (Fig. 2b, Supplementary Fig. 3B).

In line with these observations in vitro, severely reduced transcription in *Ercc1*^Δ/−^ livers (16-week old, Fig. 1a) is paralleled by higher intracellular concentration of ATP and ATP/ADP ratio (Fig. 2c, d); as expected, phosphorylation of AMPK, which responds to ATP deficit rather than its surplus, is unchanged in NER mutants (Supplementary Fig. 4A, B). The ATP surplus in *Ercc1*^Δ/−^ mice, however, is not caused by potentiation of the major biochemical pathways producing ATP: mitochondrial respiration and glycolysis. Analysis of freshly isolated primary hepatocytes, in fact, revealed that mitochondrial basal respiration was comparable in *Ercc1*^Δ/−^ and controls. In addition, measures of oxygen consumption rates show that mitochondria from *Ercc1*^Δ/−^ specimens present features such as diminished reserve capacity (i.e. induced by the uncoupler FCCP) and rotenone-sensitive respiration–which is principally attributable to complex I–that rather point to bioenergetics deficits (Fig. 2e, f). Additionally, in *Ercc1*^Δ/−^ liver glycolysis is reduced in both basal conditions and under stimulation with glucose (Fig. 2g). Consistently, *Ercc1*^Δ/−^ mice display reduced mRNA levels of pyruvate dehydrogenase beta subunit (*Pdhb*), which converts the end-product of glycolysis pyruvate into acetyl-CoenzymeA (acetyl-CoA) for the Krebs’ cycle. Further suppression at the level of Kreb’s cycle entry point is indicated by reduced mRNA levels of cytoplasmic Acetyl-Coenzyme A Synthetase (*Acss2*), which is also involved in the production of acetyl-CoenzymeA, and of ATP-citrate synthase (*Acly*), which catalyzes the condensation of oxaloacetate and Acetyl-CoenzymeA to generate citrate (Supplementary Tables 3,4). Collectively, these data strengthen the concept that increased ATP levels are the consequence of decreased consumption rather than enhanced production.

Analysis of glycolytic function, however, also reveals that maximal capacity after administration of ATP synthase inhibitor oligomycin–which completely redirects ATP production on glucose metabolism–is retained in *Ercc1*^Δ/−^ liver (Fig. 2g). This evidence suggests that the molecular machinery for glucose catabolism is present in *Ercc1*^Δ/−^ mutants but, unless stimulated by drastic conditions (i.e. oligomycin administration), its function is suppressed. Importantly, the rapid completion of the glycolysis assay ( <1 h) excludes that these differences are caused by altered transcription. In fact, steady state mRNA levels of key glycolytic enzymes are unchanged in *Ercc1*^Δ/−^ mice (Fig. 2h, Supplementary Table 4). Collectively, these elements suggest allosteric modulation as alternative regulatory mechanism.

### ATP alterations cause redesign of carbohydrate fluxes

**Fig. 3:**
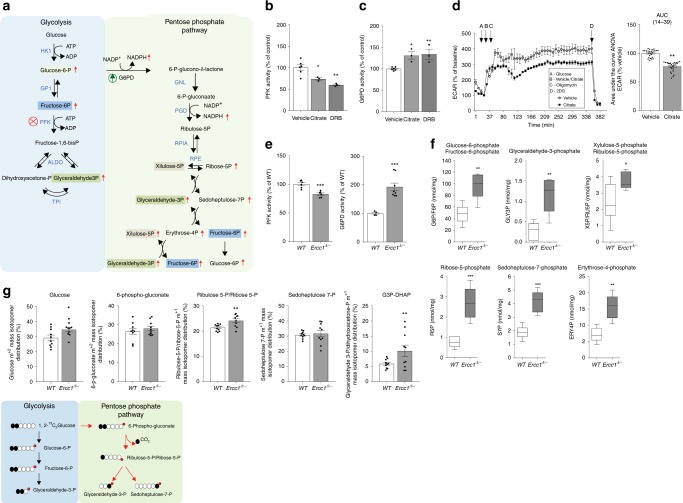
Potentiation of the pentose phosphate pathway (PPP) in *Ercc1*^Δ/−^ mice. **a** Schematic of the glycolysis and PPP interconnected pathways. Some sugar phosphates are generated in both pathways and are highlighted by same color boxes (description of abbreviations in Supplementary table 6). Enzymes’ names are in blue font. Red arrows indicate molecules that were increased in *Ercc1*^Δ/−^ liver extracts. **b**, **c** The PFK inhibitor citrate and the transcription inhibitor DRB down-regulate PFK enzymatic activity–the glycolysis rate limiting step–and potentiate the G6PD activity in primary fibroblasts. *n* ≥ 3, data were collected from three independent experiments. **d** The PFK inhibitor citrate induces reduction in the glycolytic flux, measured as ECAR in extracellular flux analysis (left trace). Treatment with citrate for 6 h is sufficient to reduce glycolysis to 77.2% of its original value (right graph). *n* ≥ 4; data were collected from two independent experiments. **e** PFK and G6PD enzymatic activities are respectively significantly reduced and increased in 16-week old *Ercc1*^Δ/−^ mice (*n* ≥ 4 per group). **f** Sugar-phosphate quantitative metabolomics indicates an increase in vivo of several metabolites involved in the PPP. Box and whiskers represents data from *n* ≥ 4 16 weeks old mice per group. **g** Metabolic tracing experiments indicate augmented flux of carbohydrates through the PPP. Cells were incubated with [1,2^13^C_2_]Glucose (2 mM) for 6 h prior to HPLC-coupled mass spectrometry analysis. The schematic (bottom panel) illustrates how the different measurable isotopes are generated from [1,2^13^C_2_]Glucose; the red dot represents the phosphate group. *n* = 10 independent biological replicates extracted from *n* = 3 mice per group. Error bars indicate mean ± s.e.m. *p < 0.05, **p < 0.01, ***p < 0.001; one way ANOVA followed by Dunnet’s test in **b**, **c** or Student’s *t*-test in **e**–**g**. Original data are provided as a Source Data file

Besides its cardinal role in bioenergetics, ATP functions as a potent allosteric regulator. For instance, in circumstances of energetic surplus, high ATP levels inhibit the master regulator of glycolysis, phosphofructokinase (PFK), via a negative feedback loop preventing superfluous glucose catabolism^24–26^. We therefore speculated that abnormally elevated ATP levels–as those induced by transcription stalling–might elicit similar metabolic rearrangements. First, we tested the effects of chemical inhibition of transcription on PFK activity in cell cultures and found that, in DRB-treated samples, increased ATP levels (shown in Fig. 2a) are paralleled by significantly decreased PFK activity (Fig. 3b), consistently with reduced basal glycolysis in *Ercc1*^*Δ/−*^ livers (described in Fig. 2g). Decreased PFK activity, and thus inhibition of glycolysis, may in turn favor glucose catabolism through the alternative pentose phosphate pathway (PPP), which branches from the glycolytic pathway itself (Fig. 3a, Supplementary Table 5). To investigate whether allosteric inhibition of PFK activates the PPP switch, human primary fibroblasts were treated with the PFK allosteric modulator citrate^27^, which is internalized via specific membrane transporters expressed in this cell type (Supplementary Fig. 5A). Citrate treatment caused reduction of both PFK activity and downstream glycolysis (Fig. 3b, d) paralleled by increased activity of glucose-6-phosphate dehydrogenase (G6PD) (Fig. 3c) i.e. the entry-step for glucose in the PPP. These elements support the notion that PFK inhibition favors glucose re-routing to the PPP through the G6PD node.

Hence, we evaluated the possibility that elevated ATP levels in livers of 16-week old *Ercc1*^Δ/−^ mice might be also associated with glucose re-routing through the PPP. Indeed, *Ercc1*^Δ/−^ liver extracts exhibit both reduced PFK and increased G6PD activity (Fig. 3e), which is in turn associated with significantly augmented levels of PPP metabolic intermediates (Fig. 3f). Metabolites tracing with [1–2^13^C_2_] glucose further confirmed potentiation of the PPP in *Ercc1*^Δ/−^ (Fig. 3g). Observations in *Ercc1*^Δ/−^ in vivo therefore recapitulate the paradigm of citrate-induced glucose rerouting in cell cultures. Importantly, transcriptomic analysis failed to detect any differentially regulated genes in the PPP, therefore supporting that glucose rerouting occurs through an allosteric mechanism (Supplementary Table 5).

### Metabolic redesign culminates reductive redox stress

**Fig. 4:**
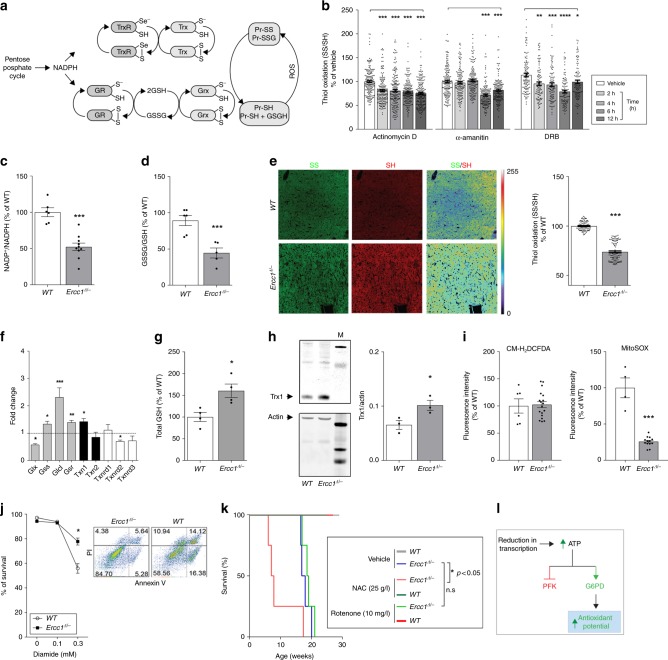
Effects of genomic instability on redox homeostasis. **a** Schematic showing the glutaredoxin and thioredoxin major redox circuits. PPP-generated NADPH provides the electrons required to maintain the circuits in a reduced state. **b** Chemical inhibition of transcription in primary fibroblasts leads to reduction of the thiol/disulfide redox couple. *n* ≥ 150, 4 mice per group. Data were collected from three independent experiments. **c**–**e** Liver extracts of 16 weeks old *Ercc1*^Δ/−^ show significant reduction of the redox state in the NADP^+^/NADPH, GSSG/GSH, and thiol/disulfide redox couples. *n* ≥ 4 per group. **f** Increased mRNA levels in redox-controlling genes, including genes involved in GSH metabolism, in livers from 16 weeks old *Ercc1*^Δ/−^ (genes abbreviations follow NCBI official nomenclature and are described in Supplementary table 7). *n* = 3 mice per group. **g**, **h** Upregulation of genes in the GSH and Trx circuits in *Ercc1*^Δ/−^ is paralleled by increased levels of GSH and of the protein thioredoxin (Trx1). Graph in H represents the densitometric analysis of the bands, *n* = 3. **i** Unchanged ROS production measured with CM-H_2_DCFDA in freshly isolated hepatocytes from 16-week old *Ercc1*^Δ/−^. Mitochondrial superoxide generation measured with MitoSOX is significantly lower in *Ercc1*^Δ/−^ mice. *n* ≥ 3 per group. Cells were analyzed by fluorescence assisted cell sorting (FACS). **j** Annexin V/propidium iodide cell death assay and FACS analysis reveal decreased sensitivity of *Ercc1*^Δ/−^ mouse embryonic fibroblasts show to the pro-oxidant diamide. Panels in the right show the percentage of surviving population (bottom left square) in mutant and WT after treatment with 0.3 mM diamide. *n* ≥ 3 per group. **k** Survival curve showing increased sensitivity of *Ercc1*^Δ/−^ mice to the antioxidant N-acetylcysteine, but not to the pro-oxidant rotenone, *n* = 4 per group. *p < 0.05, Mantel–Cox test. *WT* mice do not show sensitivity to treatments (100% of survival, lines are overlapping in the graph). **l** Schematic describing the mechanism by which transcription-blocking lesions trigger an acute antioxidant response via ATP accumulation. Error bars indicate mean ± s.e.m. *p < 0.05, **p < 0.01, ***p < 0.001, one way ANOVA by Dunnet’s test in **b** and **f**; Student’s *t*-test in **c**–**j** Original data and images are provided as Source Data file

Glucose rerouting from glycolysis to the PPP constitutes a rapid metabolic strategy to counteract oxidant stress. This pathway provides reducing equivalents in the form of NADPH, the upstream driving force to maintain redox homeostasis via the glutathione (GSH) and thioredoxins (Trxs) based thiol-disulfide circuits (Fig. 4a)^28^. Accordingly, our previous findings in hepatocytes and fibroblasts–showing that persistent chemical inhibition of transcription (4 h) leads to increased intracellular levels of ATP, diminished PFK activity, and decreased glycolysis (Figs. 2a,  3b, Supplementary Fig. 3)–are paralleled by concomitant reduced redox state (Fig. 4b). These were general effects, genuinely attributable to decreased transcription, because three inhibitors acting through different mechanisms–alpha-amanitin, Actinomycin D, and DRB–elicited the same effects on glycolysis and redox homeostasis.

In vivo, in livers of 16-week old *Ercc1*^Δ/−^ mice, the increased flux of carbohydrate through the PPP mice is paralleled by a significant reduction of the NADP^+^/NADPH redox couple (Fig. 4c), which culminates, as expected, in the abnormal reduction of the GSSG/GSH redox couple (Fig. 4d) and of the global thiol-disulfide equilibrium (Fig. 4e), which was determined by the ratiometric measurement of oxidized and reduced cysteines specifically derivatized with maleimide-conjugated fluorophores^29,30^.

These metabolic rearrangements are also accompanied by a robust increase in the biosynthesis of molecules controlling redox homeostasis via thiol-disulfide mechanisms. In particular, NER/TCR-defective mice show selective increased transcription of glutamate-L-cysteine ligase (*Glcl*) (Fig. 4f), a key enzyme in the synthesis of glutathione, and increased GSH and Trx-1 protein levels (Fig. 4g, h). Intriguingly, at 16 weeks of age, the strong antioxidant response is not associated with increased production of reactive species (RS), as indicated by reduced signal in freshly dissociated *Ercc1*^Δ/−^ hepatocytes loaded with the generic RS sensor CM-H_2_DCFDA or with the mitochondrial superoxide probe mitoSOX (Fig. 4i). Consistently, protein carbonyls–which are a footprint of RS^31^ because protein repair capacity is very limited and largely confined to the reduction of oxidized sulfur-contain amino acids- are lower in 16-week old *Ercc1*^Δ/−^ livers compared to wild-type controls (Supplementary Fig. 6). Overall, potentiation of the PPP and increased reduction of the NADP^+^/NADPH redox couple without parallel increase in ROS cause a counterintuitive reduced redox state in *Ercc1*^Δ/−^ liver.

To further probe the reductive redox balance in *Ercc1*^Δ/−^ mutants, we performed toxicity assays in vitro and in vivo to assess sensitivity to chemicals targeting the intracellular redox state. Murine *Ercc1*-deficient fibroblasts display increased resistance to the pro-oxidant diamide (Fig. 4j), which specifically promotes formation of disulfides^32^. Consistently, chronic treatment of *Ercc1*^*Δ/−*^ mice to the pro-oxidant toxin rotenone dissolved in drinking water did not shorten lifespan.

On the other hand, chronic exposure to the reducing agent N-acetylcysteine (NAC)–which has been extensively used as a therapeutic antioxidant also in humans–significantly shortened lifespan in *Ercc1*^*Δ/−*^ mice (Fig. 4k), pointing to abnormally reduced environment in this mutant strain.

### Manipulation of ATP levels elicit metabolic redesign

Collectively, our findings identify a mechanism coupling defects in NER and TCR, and transcription decline to abnormal redox reduction stress via ATP surplus and metabolic redesign. Additionally, chemical inhibition of transcription in vitro recapitulates the findings in TCR-defective mouse mutants.

**Fig. 5:**
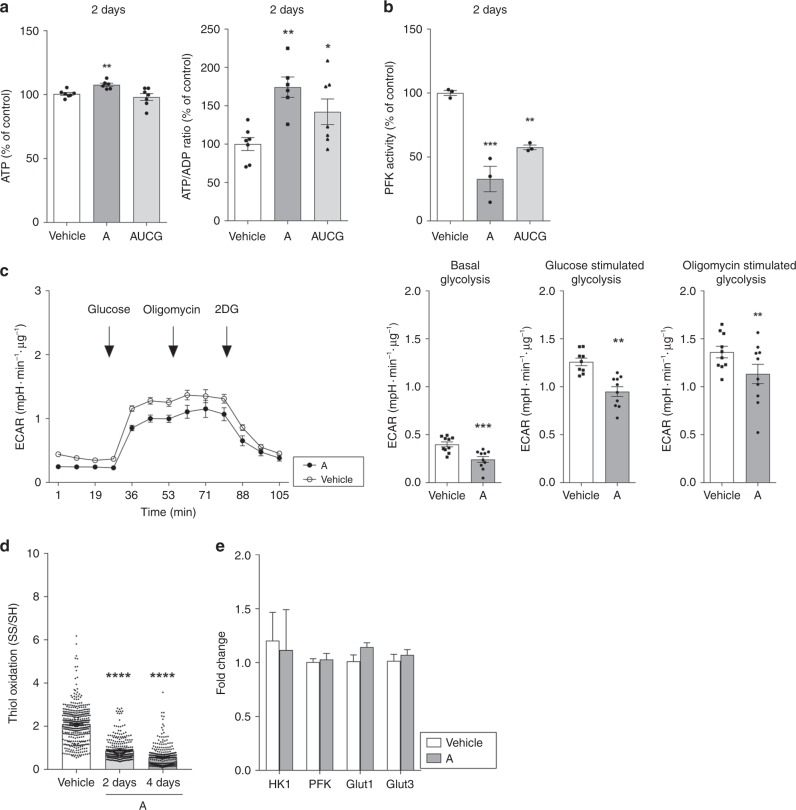
Exogenous treatment with nucleosides in cell cultures reproduces the main features observed in *Ercc1*^Δ/−^-deficient animals. **a** Two days exposure of mouse fibroblasts to exogenous adenosine (50 nM–A) or to a mixture of the four nucleosides (50 nM–AUCG) results in significant increase of ATP levels and ATP/ADP ratio. *n* = 7 from three independent experiments. **b**–**d** Nucleosides administration results in decreased PFK activity, diminished glycolysis measured as ECAR in extracellular flux analysis, and reduction in the thiol/disulfide redox couple measured by monitoring the emission of reduced and oxidized cysteines labeled with maleimide-conjugated fluorophores. *n* = 3 in B, *n* ≥ 150 in D from three independent experiments. Line graphs in C represent real time glycolysis changes in basal condition and upon stimulation with glucose and oligomycin; *n* = 10. Data were collected from three independent experiments. **e** Quantitative PCR indicates that expression of genes coding for glucose transporters and glycolysis rate-limiting enzymes remains unaltered after nucleosides administration. *n* ≥ 8, data were collected from 2 independent experiments. Error bars indicate mean ± s.e.m. *p < 0.05, **p < 0.01, ****p < 0.0001 one way ANOVA followed by Dunnet’s test in **a**, **b** and **d** or Student’s *t*-test in **c**, **e**. Original data are provided as Source Data file

To further explore this model, we manipulated the crucial driving factor in the process, i.e., intracellular ATP concentration in mouse primary fibroblasts, by administration of nucleosides, i.e., adenosine or a mix of the four nucleosides adenosine, uridine, cytidine, and guanosine (AUCG. Fig. 5a). After uptake, nucleosides promote synthesis of nucleic acid precursors via the salvage pathway^33,34^, which is energetically favored over expensive de novo synthesis. ATP expenditure is therefore reduced with consequent increase of ATP intracellular levels^35,36^. We used PFK and G6PD activity, as well as redox balance as metabolic readout measures.

Two days treatment of primary fibroblasts with adenosine, or with the combination of the four nucleosides, elevated ATP/ADP ratios (Fig. 5a) and reduced PFK activity (Fig. 5b), even though we failed to detect significant differences in G6PD in these conditions (not shown). These effects were more pronounced in the treatment with adenosine alone, which was therefore used in subsequent experiments. As expected, adenosine treatment inhibited glycolysis (Fig. 5c) and shifted thiol-disulfide equilibria towards a reduced state (Fig. 5d). Metabolic reprogramming was not due to down-regulation of glycolytic enzymes at transcriptional level or by decreased bioavailability of glucose, as evidenced by unaltered expression levels of key glucose transporters (Fig. 5e).

Alterations in glucose metabolism, including impaired glycolysis, characterize a subgroup of metabolic inborn errors that often manifest at very early life stages^37^. We therefore questioned whether the defects associated with *Ercc1* mutation manifest in very young individuals. We measured transcription levels, ATP concentration, PFK and G6PD activity, NADP^+^/NADPH ratio, and thiol/disulfide redox state also in young *Ercc1*^Δ/−^ mice. Four-week old animals failed to exhibit alterations in these hallmarks (Supplementary Fig. 7A-F, H) despite an increase in transcription of redox genes (Supplementary Fig. 7G) and the decrease in transcription output reported in this study (Fig. 1a). Reduction of transcription at 4 weeks, however, was less pronounced than at later time points (Fig. 1a). These data indicate that detected metabolic changes are progressive in time and appear later in life, during aging, lending support to the possibility that the phenomena observed in *Ercc1*^Δ/−^ mice are caused by time-dependent accumulation of cellular alterations, possibly DNA damage.

Altogether, these elements suggest that a persistent diminishment in transcription output redesigns glucose metabolism and augments the reductive capacity of the cell, and propose a further connection between defective DNA repair, metabolism, and aging.

### Glucose rerouting and reductive stress in the *Xpg*^−/−^ NER model

**Fig. 6:**
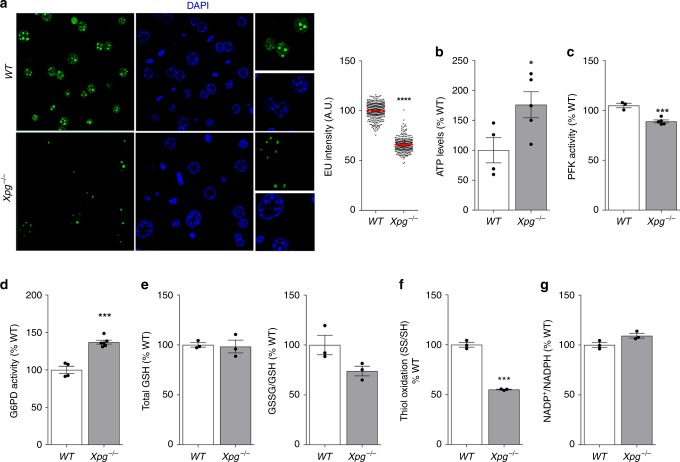
Experiments in 14 weeks old *Xpg*^−/−^ NER model recapitulate the data obtained in *Ercc1*^Δ/−^ mice. **a** Reduction in transcription levels in vivo measured by EU incorporation. EU was injected in *WT* and *Xpg*^−/−^ mice 14- week old (*n* = 3 per group). Horizontal bars in the scatter dot plots represent the average fluorescence intensity, *n* ≥ 200 cells from 3 mice per group. **b**–**d** Increased ATP levels paralleled by reduced PFK and increased G6PDH activity. *n* ≥ 4 per group. **e**–**g** Reduced redox state in *Xpg*^−/−^ mice as indicated by the GSSG/GSH and thiol/disulfide redox couples. *n* = 3 per group. Error bars indicate mean ± s.e.m. *p < 0.05, **p < 0.01, ***p < 0.001. Student’s *t*-test. Original data are provided as Source Data file

We next asked if ATP-mediated rerouting of glucose through the PPP to produce excess reducing equivalents in the form of NADPH were general consequences of defective TCR augmented by a GG-NER defect rather than other repair defects associated with *Ercc1* deficiency. We therefore extended analysis to another repair mutant with a combined GG-NER/TCR defect, the *Xpg* knock-out mouse (*Xpg*^−*/−*^)^38^. Here, we evaluated the hallmarks of metabolic redesign previously observed in *Ercc1*^Δ/−^ mice: transcription decline, increased ATP, PFK inhibition, G6PD activity, and reduced thiol/disulfide redox state. 14-weeks-old *Xpg*^−/−^ mice indeed display all of these features (Fig. 6a–g), recapitulate previous observations in *Ercc1*^Δ/−^, and therefore demonstrate the general nature of our findings.

### Metabolic reprogramming in the TCR disease Cockayne syndrome

To determine the relevance of our findings for human diseases, we investigated segmental progeroid syndromes caused by GG-NER/TCR defects, specifically in primary fibroblasts^39,39^ from a patient (96RD235) suffering profound GG-NER/TCR defects due to a very severe mutation caused by a deoxycytidine deletion in the codon coding for the Arg45 in the NER gene *XPG*, that causes a C-terminal truncation in the protein^39–41^. The severe clinical outcome combines xeroderma pigmentosum and Cockayne syndrome (XP/CS) symptoms, similar to the *Xpg*^*−/−*^ mouse model used above^42^.

**Fig. 7:**
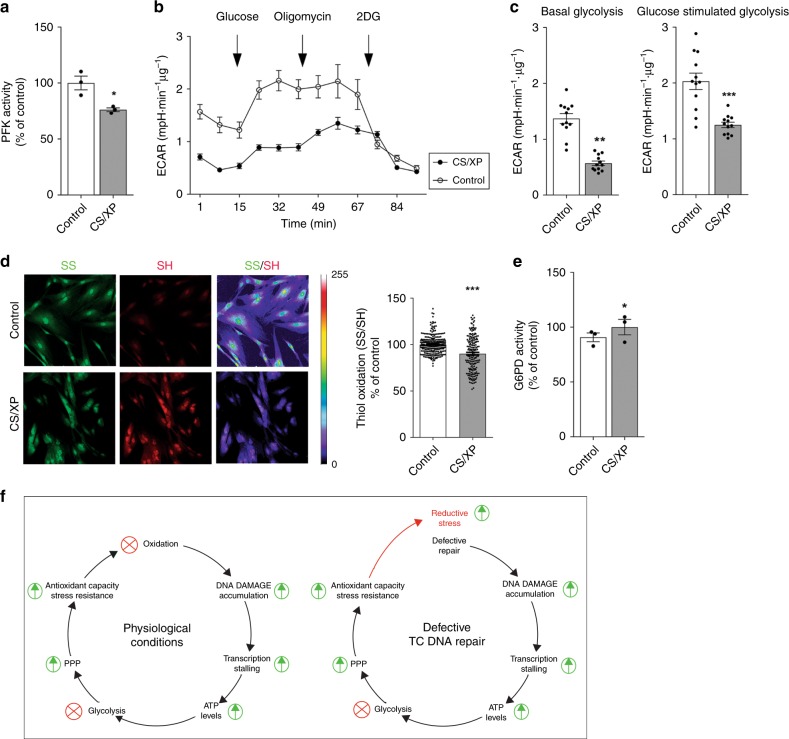
Primary fibroblasts derived from skin biopsy from a patient suffering profound GG-NER/TCR defects due to a gene *XPG* mutation show metabolic modifications. **a** PFK activity is reduced in the patient’s cell and **b**, **c** is paralleled by decreased glycolysis, measured as ECAR in extracellular flux analysis. Line graph in B represents real time glycolysis changes in basal condition and upon stimulation with glucose and oligomycin. *n* ≥ 3, data were obtained from two independent experiments. **d** In the patient’s cells, the thiol-disulfide redox equilibrium, which was measured after derivatization of oxidized and reduced cysteines with maleimides conjugated with different fluorophores, is shifted toward a reducing state. *n* ≥ 150, data were obtained from three independent experiments. **e** G6PD activity shows a consistent trend, which however did not reach statistical significance. *n* = 3, data were obtained from two independent experiments. **f** Schematic representing the model we propose. Accumulation of transcription-blocking lesions triggers an acute antioxidant response driven by metabolic redesign. In normal conditions, pro-oxidant species are the principal source of DNA damage. However, in GG-NER/TCR-deficient mice DNA damage accumulation is not caused by increased oxidative stress, but is rather due to compromised repair. Thus, the intrinsic antioxidant response in this case is not paralleled by increased reactive species and it culminates in an overall reduced redox state. Error bars indicate mean ± s.e.m. *p < 0.05, **p < 0.01, ***p < 0.001, Student’s *t*-test. Original data are provided as Source Data file

Previous studies have already demonstrated increased ATP levels in CS primary fibroblasts;^43^ here, we show that XP/CS primary fibroblasts also exhibit reduced PFK activity (Fig. 7a) paralleled by decreased glycolysis (Fig. 7b, c) and alterations in the thiol-disulfide redox equilibria toward a reducing state (Fig. 7d). G6PD activity showed a consistent trend, which however did not reach statistical significance (Fig. 7e). These findings confirm the principal observations in the mouse repair mutants and extend them to the corresponding human disorder.

## Discussion

In this study we demonstrate a progressive dramatic decrement of transcription in NER/TCR-defective mouse models. Although, ERCC1 and other NER/TCR factors have also been associated with transcription regulation^44^, we favor the idea that–rather than being caused by anomalies in or lack of specific transcription factors–the collapse of RNA synthesis observed in the liver of *Ercc1* and *Xpg* mutant mice is the consequence of compromised repair function (and consequently DNA damage). This possibility is supported by multiple arguments: first, the drastic and progressive decline of overall transcriptional output (up to 90%) (Fig. 1a) is difficult to reconcile with anomalies in or lack of a specific transcription factor. These molecules, in fact, control expression of a specific subset of genes and defects in a specific factor would affect expression of its target genes, with immediate rather than progressive consequences, the latter occurring over a timespan of months. Second, here and in a previous study^17^ we reported that transcription decline is gene length-dependent (Fig. 1c); hitherto, no transcription factor has been described that regulates genes based on gene size, at least to our best knowledge. Third, the gene length effect is accurately recapitulated in a dose-dependent fashion when cultured cells are exposed to transcription-blocking UV damage, and is exaggerated and permanent in TCR mutants^45^. Fourth, our observation that unaltered levels of elongating Ser2-phosphorylated RNA Pol II in aged *Ercc1* liver (Fig. 1d) are paralleled by drastic reduction in total transcription output, which in *Ercc1* mutants is only 10% of controls, implies that many RNA Pol II molecules in elongation mode are not productive; this evidence is consistent with DNA damage mediated RNA Pol II stalling. Altogether, these arguments strongly favor time-dependent accumulation of DNA damage as the principal cause of the transcription stress. Unfortunately, hitherto no reliable methods exist to accurately measure physiological levels of spontaneous DNA lesions, with the exception of cyclopurines, which have been reported to be elevated in liver of *Ercc1* mouse mutants^46^. Since the latter oxidative NER lesions are capable of transcription stalling^47,48^, they are candidates for at least part of the transcription problems described in this work.

Our study proves that transcription arrest in defective DNA repair organisms leads to ATP surplus, which in turn inhibits the key glycolytic enzyme PFK to promote glucose rerouting through the PPP and ultimately favors production of reducing equivalents in the form of NADPH (Fig. 7f). Thus, we provide evidence for an adaptive mechanism that couples defects in TCR and consequent transcription stalling with redesign of glucose metabolism and activation of antioxidant defenses. The process can be framed as part of the ‘survival response’ that we described at transcriptional level previously;^4,49,50^ in this study, we extend these findings demonstrating that metabolic reprogramming depends on ATP-dependent allosteric mechanisms and we therefore reveal a faster cellular response to DNA damage. In fact, unchanged levels of glycolytic enzymes mRNA and the rapid time-course of cell culture experiments (i.e., insufficient to observe transcription effects) indicate that the process is independent of and precedes the well-documented transcriptional response. Elevated ATP levels in NER-deficient primary human cell have been previously described in other independent studies^43^. The consequences of altered levels of this potent allosteric modulator on metabolism and its link with transcriptional stress, DNA damage, and the antioxidant response, however, have to our best knowledge never been described. Obviously, additional ATP based mechanisms independent from allosteric modulation may participate in the adaptive cellular response, possibly by reshaping the transcriptional landscape. In bacteria, for instance, it has been demonstrated that both high and low intracellular ATP concentrations affect macromolecular synthesis^11,51^ and that some promoters are directly activated by ATP^11,52^. These mechanisms, however, have not been investigated in comparable detail in eukaryotic systems–at least to our best knowledge–and further studies will be necessary to elucidate how intracellular ATP concentration regulates the transcriptome.

Of note, we provide evidence that chemical inhibition of RNA pol II recapitulates the features observed in NER mutants; however, additional studies will have to address also the contribution of both RNA pol I to the observed metabolic changes, given that TC-repair can amend lesions in ribosomal DNA^16^.

Increasing evidence highlights the relevance of metabolism modulation and of the response to DNA damage in the complex process of aging;^53,54^ however, whether and possibly how they are related has remained largely elusive. Metabolic reprogramming to favor the PPP over glycolysis in DNA repair deficient systems may be framed as part of the survival response previously described in segmental progeria^4,7,50^. Glucose metabolism, in fact, plays a major role in the aging process and has a profound influence on lifespan, as indicated by the relevance of IGF-1/insulin signaling for longevity. Moreover, reduction of glucose metabolism–and of glycolysis in particular–have been even proposed as mimetics of dietary restriction, the most efficient form of intervention to delay aging^55^, even most prominently in DNA repair deficient accelerated aging mutants^17^.

One apparent consequence of rerouting metabolism from glycolysis through the PPP is to mount a first-line defense against oxidation, which is critical to counteract stress^56^ including genomic stress caused by oxidative DNA damage. The nexus between activation of the PPP shunt and DNA damage is not unprecedented. For instance, glucose-6-phosphate dehydrogenase (G6PD), which catalyzes the first reaction in the PPP, protects against endogenous DNA damage^57^. Increased activation of G6PD can be mediated by the DNA damage response protein ATM, which is a primary sensor for double-strand breaks, to stimulate the PPP pathway, synthesis of nucleotides, and antioxidant response^58^. In this case, however, PPP activation proceeds through Hsp27-mediated G6PD stabilization.

An additional link between the PPP and ATM stems from observations in rheumatoid arthritis patients, who display deranged DNA repair in T-cells^59,60^. Here, an excessively reduced redox environment prevents ATM activation, which in turn allows T-cells bypassing the G2/M checkpoint. The latter results in augmented proliferation and consequent enrichment of the pro-inflammatory pool population^61^. Accordingly, administration of pro-oxidants in rheumatoid arthritis counterbalances the abnormally reductive redox environment, restores redox signaling, and suppresses synovial inflammation^62^.

These independent results from our and other laboratories consistently point to a key role of the PPP in the response to DNA damage, demonstrating that multiple pathways converge on its potentiation to compensate for genomic stress. Unlike previous evidence, however, our study reveals a mechanism based on allosteric regulation, a biochemical strategy allowing highly rapid adaptation of cellular function against stress.

Augmented redox-buffering capacity is intrinsic to longevity and is a prerogative of long-lived species^63^. The increased availability of redox-sensitive thiol groups, which we found in GG-NER/TCR-deficient mice, is fundamental to counterbalance oxidative stress. In this work we have shown that short-lived, progeroid NER-deficient mice display an abnormally reduced redox state; albeit surprising and counterintuitive given the notion that oxidation increases with age^64^ and that longevity -instead of a short lifespan- is associated with enhanced redox-buffering. However, our findings are in line with previous reports showing higher sensitivity of fibroblasts from patients with severe DNA repair defects to the thiol-reducing agent dithiothreitol (DTT)^65^; while this feature could be caused by effects not directly related to reducing stress–for instance DTT mediated ER stress and consequent failure to properly fold proteins–it certainly establishes a nexus between reductive redox stress and defective DNA repair. To explain this apparent paradox, we propose the model depicted in Fig. 7f. Our data demonstrate that accumulation of transcription-blocking lesions triggers an acute antioxidant response driven by metabolic redesign and indicate that evolution selected mechanisms where DNA damage sensing and antioxidant capacity potentiation are intrinsically coupled. This concept is consistent with the dominant idea that, in normal conditions, pro-oxidant species are a principal source of DNA damage. In GG-NER/TCR-deficient mice, however, DNA damage accumulation is not caused by increased oxidative stress, but is rather due to compromised repair. Thus, the intrinsic antioxidant response in this case is not paralleled by increased reactive species and it culminates in an overall reduced redox state, as we have shown in repair-deficient liver samples (Fig. 4e). Of note, abnormal reductive environment–which is deleterious and has been associated with hyperactivation of the PPP in some forms of cardiomyopathies and in the autoimmune syndrome rheumatoid arthritis^62,66^–might also be implicated in the pathogenesis of diseases caused by GG-NER/TCR defects (i.e. CS and XP) and its role in the progression of these disorders should be explored further. Furthermore, future investigations should determine whether chemotherapeutic agents acting through mechanisms involving transcription block–for instance platinum-based anti-neoplastics–involuntarily potentiate the PPP and antioxidant defenses in cancer cells, therefore diminishing the efficacy of the treatment. Finally, our findings suggest that trials with antioxidants might not be recommended for DNA repair deficient progeroid patients and further research is certainly required to determine the validity of such strategies in these diseases.

Multiple lines of evidence support a causative role for DNA damage accumulation and TCR defects in natural aging^1^ and associated morbidities, for instance Parkinson’s and Alzheimer’ disease^67–69^. Moreover, we found perturbation in transcription to be intrinsic to the aging process from both a qualitative and quantitative standpoint: transcriptional output decreases in natural aging^17,70,71^ and studies at single cell level demonstrated an age-related increase in variance and background noise in gene expression^72^. Our findings therefore strengthen the parallels between accelerated and natural aging, confirm the validity of the repair mutants, and highlight declining transcription as a novel important parameter in the aging process. Future studies are warranted to determine in greater detail the contribution of transcription decline to natural aging and physiological deterioration. In conclusion, our study provides mechanistic insights about the metabolic consequences of DNA damage accumulation and paves the road for future investigations in multiple fields of biomedical sciences.

## Methods

### Reagents

All reagents were purchased by Sigma-Aldrich unless otherwise specified.

### Animals

The generation and characterization of TC-NER deficient *Ercc1*^Δ/−^ and *Xpg*^*−/−*^ mice has been previously described^15,38^. Mice were generated in an FVB:C57BL/6 J (50:50) genetic background and wild type littermates were used as controls. Animals were kept on a regular diet and housed at the Animal Resource Center (Erasmus University Medical Center), which operates in compliance with the “Animal Welfare Act” of the Dutch government, following the “Guide for the Care and Use of Laboratory Animals” as its standard. This study was approved by the Erasmus University Medical Center ethical committee for animal welfare. Mutant mice and respective controls (males and females) were used at the age of 4, 16, and 20 weeks old for *Ercc1*^Δ/−^ and 14 weeks old for *Xpg*^*−/−*^.

### Primary hepatocytes isolation

Primary hepatocytes were isolated from 4 to 16 and 20 weeks old animals by the collagenase perfusion method^73^, with modifications. Briefly, liver was perfused via cannulation of the abdominal vena cava inferior using wash buffer (HBSS modified with 10 mM HEPES and 16 mM NaHCO_3_) at 37 °C at a flow rate of 7 ml/min. After 10 min, the buffer was switched to the perfusion solution (HBSS, 10 mM HEPES, 16 mM NaHCO_3_, 150 U/mL collagenase type IV (Sigma) and 5 mM CaCl_2_) and the liver was perfused for 14 min. The liver was then excised, minced and dissociated tissue was filtered using a 70 µm sterile filter in washing medium (DMEM containing 10% FBS, 1% Pen/Strep, 0.5 U/mL insulin, 20 ng/mL murine EGF, 7 ng/ml glucagon and 7.5 µg hydrocortisone). Parenchymal cells were purified by centrifugation (50 g, at 4 °C for 10 min) using Percoll (GE Healthcare) in 1:1 dilution with wash medium. Viability, which was typically around 90%, was determined using Trypan Blue exclusion. Isolated liver cells were then seeded on previously Cell-Tak (22.4 μg/ml; 354240, BD Biosciences) coated plates or coverslips, and maintained in DMEM containing 10% FBS, 1% Pen/Strep.

### Nascent RNA synthesis detection in primary cells and tissues

Quantification of nascent RNA synthesis was performed with Ethylene uridine (EU) taking advantage of the copper (I)-catalyzed cycloaddition reaction between azides and terminal alkynes, as described in a previous study^12^. The experiments were performed using the Click-iT Nascent RNA capture kit (C10365, Invitrogen).

To detect de novo transcription in primary hepatocytes, 3 mM EU was dissolved into the culture medium for 2 h. Cells were then washed twice with PBS, and then blocked with 3% BSA in PBS for 30 min. The reaction was then performed according to the manufacturer’s instructions.

To measure de novo transcription in mouse tissues, animals received an intraperitoneal injection (i.p.) of EU (0.088 mg/g in sterile PBS). Tissues were harvested 5 h after injection and were fixed in paraformaldehyde overnight, kept in 30% sucrose for additional 24 h, embedded in Tissue-Tek CRYO-OCT (1437365, Fisher Scientific) and cryostat-cut in 10 μm sections. For the staining, the sections were rinsed with PBS, incubated 30 min in a PBS solution containing 3% BSA and then treated at room temperature with a solution containing 100 mM Tris, pH 8.5, 1 mM CuSO_4_, 25 µM tetramethylrhodamine-5-carbonyl azide and 100 mM ascorbic acid for 30 min. The sections were then washed twice with PBS-BSA, 2 with PBS, and finally stained with DAPI (10236276001, Sigma) or SYTOX Green nucleic acid stain (S7020, Invitrogen). Both cells and sections were imaged with a Leica TCS SP5 laser scanning confocal microscope. EU intensity was analyzed in at least 150 nuclei per line in a semi-automated fashion using constant thresholding parameters with the Metamorph software(Molecular Devices) or ImageJ software.

### Bioinformatics

Microarray data Affymetrix^TM^ mouse 430 V2.0 arrays have been obtained from 6 *Ercc1* ^*Δ/−*^ livers and 6 wildvtype 16 week old mice and have been used for this study. The data are available through the public repository ArrayExpress at accession code: E-MEXP-1503. Detailed description about the samples collection, RNA extraction, cDNA synthesis, microarray hybridization and scanning is included in a previous report^49^. Expression intensities were summarized, log2 transformed and normalized using RMA as is implemented in R open statistical package (http://www.r-project.org/). Linear model from Gentleman implemented in R 53 was used to calculate the fold change (FC), p value and false discovery rate (FDR) between *Ercc1* ^*Δ/−*^ and wild type samples.

To assess the relationship between gene length and fold-change (FC), probesets in the Affymetrix^TM^ array with multiple gene annotation were filtered out and BioMart 54 was used to ask the gene length for the remaining probe sets. Differentially expressed genes were selected using FDR 540.05 and linear fold change ± 1.5. Shapiro–Wilk test was applied to contrast the normality of the gene length distribution in the different list of DEG. The Mann-Whitney for non-paired samples test was used to evaluate whether the distributions of gene length of DEG genes were different between up-regulated and down-regulated genes.

In order to increase the understanding of the compromise of several cellular processes secondary to the accumulation of DNA damage, we evaluated the level expression of selected genes for core of RNA Pol II and Pentose Phosphate, Glycolysis/Gluconeogenesis and unfolded protein response (UPR) pathways as are described in KEGG database. The criteria to identify a gene as differentially expressed was the same described (FDR < 0.05 and linear fold change ± 1.5).

### Intracellular nucleotide pool measurement by HPLC

The measures were performed as previously described^74^, with minor modifications. Briefly, liver was grinded in liquid nitrogen and treated with 0.4 M HClO_4_. After centrifugation the supernatant was neutralized with 5 M K_2_CO_3_. The pellet was used for protein determination and the supernatant was used to measure and quantified the nucleotide pool by HPLC analysis (Alliance 2690; Waters, Milford, MA) using a Partisphere-SAX anion exchange column (4.6 × 125 mm; Whatman International Ltd., Maidstone, UK). The following gradient was used: 5 min with 100% buffer A (5 mM NH_4_H_2_PO_4_, pH 5); a 15-min linear gradient of 100% buffer A to 100% buffer B (300 mM ^+^NH_4_H_2_PO_4_, pH 5); 20 min with 100% buffer B; a 5-min linear gradient to 100% buffer A; and equilibration at 100% buffer A for 5 min. The UV absorbance of the peaks was recorded at 262 nm.

### RNA isolation and qPCR

Total RNA was isolated from liver or cells using Trizol (15596026, Invitrogen), according to the manufacturer’s protocol. First-strand complementary cDNA was synthesized from 1 µg RNA using SuperScript First-Strand cDNA Synthesis Kit (11904018, Invitrogen). qPCR was performed on a C1000 Thermal Cycler, CFX96 Real-Time System (Bio-Rad), SYBR Green I (4385612, Invitrogen) and Platinum *Taq* polymerase (10966-083, Invitrogen). The primer sequences are showed in Supplementary Table 7. Primer efficiency and specificity have been confirmed. Data were analyzed using the second derivative maximum method: (E1_gene of interest_^ΔCP (cDNA of wt mice - cDNA of Ercc1Δ/- mice) gene of interest)^/(E_*house keeping*_^ΔCP (cDNA wt mice- cDNA of Ercc1Δ/- mice) housekeeping gene^). Data were reported as the average of values obtained from tissues froma minimum of 5 animals ± the standard deviation.

### Bioenergetics Assays

Oxygen consumption rates (OCR) and extracellular acidification rate (ECAR) were measured using a XF-24 Extracellular Flux Analyzer (Seahorse Bioscience), as previously described^75,76^. On the experimental day, medium was changed to unbuffered DMEM (XF Assay Medium–Agilent Technologies, Santa Clara, Ca, USA) supplemented with 2 mM glutamine, 5 mM glucose and 1 mM sodium pyruvate, and incubated 1 h at 37° C in the absence of CO_2_. Medium and reagents acidity was adjusted to pH 7.4 on the day of the assay, according to manufacturer’s procedure. Hepatocytes were seeded at a density of 3 × 10^4^ cells/well on Cell-Tak (22.4 μg/ml; 354240, BD Biosciences) coated Seahorse plates and analyzed 3 h after isolation. Fibroblasts were seeded at a density of 6 × 10^4^ cells/well on uncoated Seahorse plates and analyzed after 24 h. Optimal cells densities were determined experimentally to ensure a proportional response to FCCP with cell number (data not shown).

Mitochondrial respiration was measured as oxygen consumption rate (OCR). After four baseline measurements cells were sequentially challenged with injections of mitochondrial toxins: 0.5 µM oligomycin (ATP-synthase inhibitor, which informs on the level of respiration allocated for ATP production), 0.4 μM fluoro-carbonyl cyanide phenylhydrazone (FCCP, oxidative phosphorylation uncoupler, which informs on the maximal achievable level of respiration) for hepatocytes or 1 µM for fibroblasts, 1 μM rotenone (complex I inhibitor, which informs on the level of respiration dependent on complex I) and 1 μM antimycin A (complex III inhibitor, which completely ablates mitochondrial respiration and informs on the level of non-mitochondrial respiration). Basal respiration was defined as the average OCR values at baseline; proton leakage was defined as mitochondrial respiration detected after oligomycin injection and maximal respiration was defined as OCR values after FCCP injection. Respiration dedicated to ATP production was calculated as difference between basal respiration and the respiration measured after oligomycin injection and the rotenone dependent respiration parameter–which accounts for mitochondrial complex I activity -was calculated as the difference between the maximal respiration value and the OCR values obtained after the rotenone injection.

Glycolysis was measured as extracellular acidification rate (ECAR), which reflects lactate production via glucose catabolism. On the experimental day, medium was changed to unbuffered DMEM (BASE Medium–Agilent Technologies, Santa Clara, Ca, USA) supplemented with 2 mM glutamine, 1 mM sodium pyruvate, and incubated 1 h at 37° C in the absence of CO_2_. Medium and reagents acidity was adjusted to pH 7.4 on the day of the assay, according to manufacturer’s procedure. ECAR was measured in basal conditions, after stimulation with 11 mM glucose, then with 5 μM oligomycin, and finally inhibited with 100 mM 2-deoxyglucose (2-DG).

### Sugar phosphate quantitative metabolomics

Sugar phosphates were quantified by LC–MS/MS, as previously described^28^. Briefly, metabolites were extracted in HBSS with 2% perchloric acid, and proteins were precipitated after neutralization with a phosphate buffer. The samples were subsequently supplemented with an internal isotope labeled standard ^13^C_6_-glucose-6P, separated on a water-acetonitrile gradient on a C_18_ RP-HPLC column (LC packings), and analyzed on an API3000 triple quadrupole mass spectrometer (AB/Sciex).

### Enzymatic activity assays

PFK (MAK093 Sigma) and G6PD (ab102529, ABCAM) activity was measured with commercial kits according to the manufacturer’s direction.

### ATP measurement

ATP (MAK190, Sigma) was measured with commercial kit. Briefly, tissues were homogenized in ATP assay buffer and the high-molecular components were removed by size-exclusion filtration using Amicon Ultra-2 10 K columns (Z740164, Sigma). The colorimetric assay was performed according to manufacturer’s instructions. ATP/ADP ratio was measured with the ADP/ATP Ratio Assay Kit (MAK135, Sigma) according to manufacturer’s instructions.

### NADP^+^/NADPH measurement

Tissues were homogenized in lysis buffer (0.1 M Tris–HCl pH 8.0, 0.01 M EDTA, 0.05% Triton X-100) and mixed with a same volume of a phenol:chloroform:isoamyl (25:24:1) solution. After centrifugation, the upper phase was collected and passed onto Phase Lock gel tubes (5Prime) to remove any organic solvent. The high-molecular components were removed by size-exclusion filtration using Amicon Ultra 10 K columns (Millipore). Colorimetric assays were performed using the NADP^+^/NADPH Quantification Kit (JM-K347-100 MBL) according manufacturer’s instructions.

### Glutathione measurement

Total glutathione (GSH) and the ratio between reduced (GSH) and oxidized (GSSG) glutathione in liver were analyzed by using Glutathione (GSH/GSSG/Total) Assay kit (K264-100, Biovision). The assay is based on oxidation of GSH by the sulfhydryl reagent 5,5′-dithio-bis(2-nitrobenzoic acid) (DTNB) to form 5′-thio-2-nitrobenzoic acid (TNB), which is yellow and thus spectrophotometrically measurable at 412 nm. Briefly, liver tissues were homogenized in ice-cold glutathione assay buffer and mixed with 6 N perchloric acid (PCA) to precipitate proteins. After centrifugation (13,000 × *g* for 5 min), the supernatant was neutralized with half volume of 6 N KOH. Total GSH and GSSG/GSH levels were determined according manufacturer’s procedure with a GLOMAX multi detection system spectrophotometer (Promega).

### Determination of thiol- disulfide redox equilibrium

Assays were performed as previously described, according to a procedure we refer to as redox immunohistochemistry (RHC)^29,30^. Briefly, cells or freshly cut tissue sections (10 µm) from liver were fixed with 4% paraformaldehyde, 1 mM N-ethilmaleymide, 5 µM Alexa Fluor 555-Maleimide, 0.05% Triton X-100 for 20 min. Quenching of unreacted thiols was achieved with 100 mM NEM. The samples were washed twice in PBS and then incubated for 20 min with 5 mM tris(2-carboxyethyl)phosphine (TCEP) in PBS. Samples were washed twice in PBS and the second labeling step was performed by incubating with 5 µM Alexa Fluor 488-Maleimide, 1 mM NEM for 20 min followed by two additional washing steps. Samples were acquired using a Leica SP5 laser scanning confocal microscope and analyzed using the MetaMorph software (Molecular Devices). The ratiometric disulfide/thiol redox state was calculated as the Alexa488/Alexa555 ratio. Alternatively, oxidized and reduced cysteines were labeled with IRDye 800 maleimide (Li-COR biosciences) and Alexa Fluor 680-Maleimide, and proteins were extracted from the samples and resolved by SDS-electrophoresis; the gel was scanned with an Odyssey Platform (Li-COR Biosciences) and its Image Studio Lite software was used to analyze the fluorescent signal of oxidized and reduced cysteines^29,30^.

### Flow cytometry

General ROS was measured with 5-(and 6)-chloromethyl-2′,7′-dichlorodihydro-fluresceindiacetate, acetyl ester (CM-H_2_DCFDA, C6827, Molecular Probes), while mitochondrial superoxide production was detected with MitoSOX Red (M36008, Invitrogen), according to manufacturer’s instructions. Annexin V/Propidium iodide (PI) staining was also performed according to the manufacturer’s guidelines. Briefly, cells were treated with diamide dissolved in cell media at the indicated concentrations. After 4 h, the cells were detached with trypsin, washed in ice-cold HBSS without Ca^2+^ and Mg^2+^ and suspended in 0.1 M Hepes (pH 7.4) 1.4 M NaCl, 25 mM CaCl_2_ containing Propidium Iodide (PI; P4170, Sigma) or Annexin V (550474 BD Biosciences). Cells were incubated for 15 min at room temperature and flow cytometry was immediately performed using FACSARIA or FACScan (BD Biosciences). FlowJo Software (Tree Star Inc.) was used for data analysis.

### Cell cultures

Isogenic primary mouse embryonic fibroblasts (MEF) lines were isolated from E13.5 embryos and cultured in low oxygen (3%). Human primary fibroblasts were cultured at 20% oxygen in DMEM supplemented with 10% fetal bovine serum (FBS) and 1% Pen/strep. All participants or their legal caretakers gave written informed consent according to Erasmus MC institutional review board requirements.

Depending upon the specific experiment, cells were treated with 50 nM nucleosides (adenosine, uridine, citydine and guanosine), 10 µg/ml α-amanitin (Sigma), 5 µg/ml actinomicyn D or 50 µM 5,6-dichloro-1-β-D-ribofuranosylbenzimidazole (DRB) for the time frame indicated in the experiments.

### Immunoblot procedures

Immunoblot analysis were performed according to the LI-COR Biosciences Guidelines; images have been acquired by the Odyssey Platform and analyzed by the Image Studio lite software (Li-COR Biosciences). The following primary antibodies have been used: Thioredoxin-1 (1:5000, AB9328, Millipore), β-actin (1:5000, MAB1501, Millipore), anti-SLC13A5 (1:1000, HPA044343, Sigma Aldrich); AMPK-α (1:1000; 2793, Cell Signaling Technologies); p-AMPK-α (1:1000; 2535, Cell Signaling Technologies); Ubiquitin (1:1000, rabbit polyclonal Z0458, DAKO and mouse monoclonal FK2, BML-PW8810-0500, Enzo Life Sciences); total RNA Pol II (RPB1-NTD, 1:1000, 14958 S, Cell Signaling Technologies); RNA Pol II-phosphoSer5 (1:5000, 3E10, Chromotek); RNA Pol II-phosphoSer2 (1:5000, 3E8, Chromotek); Tubulin (1:5000, T6074, Sigma). IRDye secondary antibodies (LI-COR) were used 1:7500.

Briefly, frozen liver samples were extracted in RIPA buffer (150 mM NaCl, 1% NP40, 0.5% DOC, 0.1% SDS, 50 mM Tris HCl) supplemented with protease and phosphatase inhibitor from Roche diagnostics. To preserve protein sample ubiquination, 5 mM EDTA, 5 mM EGTA and 20 mM NEM was added to the RIPA buffer before the extraction. Protein samples were loaded on precast 4–12% Bis-Tris gradient gel (NuPAGE Novex, NP0335, Invitrogen), separated with NuPAGE running buffer (NP0002, Invitrogen) and transferred on PVDF membrane (IPFL00010, Millipore).

To pull down ubiquitinated proteins from liver extracts, Tandem Ubiquitin Binding Entities (TUBE2) was used coupled to agarose purchased from Boston BioChem (AM-130). This affinity resin was washed 2 times with RIPA buffer (150 mM NaCL, 1% NP40, 0.5% DOC, 0.1% SDS, 5 mM EDTA, 5 mM EGTA, 20 mM NEM, 50 mM Tris HCl supplemented with protease and phosphatase inhibitor from Roche diagnostics). Subsequently, 1 mg protein of each liver extract (sonicated in RIPA) was incubated with 20 µl of TUBE2-agarose for 3 h on a Eppendorf rotator at 4 degrees. As a control, a mixture of the tissue lysates was incubated with 20 µl of Pierce protein G agarose beads (20398, Thermo Scientific). Following incubation, beads were washed 4 times with 1 ml of RIPA. To elute and denature ubiquitinated proteins, 70 μl 2x Laemmli sample buffer was added to the beads and boiled for 3 min. For detection of ubiquitinated proteins on western blot, the mouse monoclonal FK2 antibody from Enzo Life Sciences (BML-PW8810-0500) was used. In addition, RNA pol II (Rpb1) was stained using the phospho-specific rat monoclonal antibodies from chromotek (3E8 and 3E10).

Protein carbonylation was measured as previously described^77^. Carbonyls were derivatized with 2,4-dinitrophenylhydrazine (DNPH) in 12% SDS, 5% trifluoroacetic acid (TFA), to form 2,4-dinitrophenylhydrazone, which was in turn detected using an anti-dinitrophenyl primary antibody (1:1000, D8406 Sigma Aldrich) and a rat anti-mouse IgE-HRP secondary antibody (1:1000, 1130-05, Southern Biotech). Negative controls were prepared omitting DNPH in the reaction. Immunoblots were developed with ECL (Amersham Biosciences) and membranes were acquired using a chemioluminescent imaging system (Uvitec Cambridge).

### In vivo drug treatments

N-acetylcysteine (NAC–A5270, Sigma) and Rotenone (MP Biomedicals - 150154) have been administered to the mice in a concentration of 25 g/L and 10 mg/L respectively, dissolved in mice drinking water starting from the age of 4 weeks. The solutions were freshly prepared and replenished every 3 days. The range of NAC doses was selected on the basis of previous literature^78–80^. As far as rotenone is concerned, we took as a starting point the work of Pan-Montojo and colleagues^81^; here, a dose of 5 mg/kg principally affected the gastrointestinal tract, therefore minimizing systemic toxicity. In total 5 mg/kg correspond to 0.125 mg for an average mouse with 25 grams body weight. Here, we decided to use a slightly lower dosage than what reported by Pan-Montojo and co-workers in light of the fragile phenotype of *Ercc1* mutants and of their general sensitivity. We therefore administered 10 mg/mL rotenone in drinking water, which approximately corresponds to 0.04 mg per mouse per day given that mice (including *Ercc1* mutants) drink ~4 mL of water daily (please see http://web.jhu.edu/animalcare/procedures/mouse.html).

### Metabolic flux analysis

Isogenic primary mouse embryonic fibroblasts (MEF) derived from *WT* or *ERCC1* ^*D/−*^ were kept in culture to reach confluency (1 × 10^6^). Cells were then exposed to [1–2^13^C_2_]Glucose 2 mM (Sigma-Aldrich, 453188) for 6 h. Cells were then washed twice with cold PBS and collected with scrapers. Tubes were centrifuged at 1000 g for 10′ at 4 °C and then frozen in liquid nitrogen. Cells were then harvested in 250 µl of ice-cold methanol/acetonitrile 1:1 and spun at 20,000 × *g* for 5 min at 4 °C. Supernatant were then filtered through a regenerated cellulose filter, dried and resuspended in 100 µl of MeOH for subsequent analysis. The analysis was performed on an API-4000 triple quadrupole mass spectrometer (AB Sciex) coupled with a HPLC system (Agilent) and CTC PAL HTS autosampler (PAL System). The identity of all metabolites was confirmed using pure standards. Quantification of different metabolites was performed with a liquid chromatography/tandem mass spectrometry (LC–MS/MS) method using a cyano-phase LUNA column (50 mm × 4.6 mm, 5 μm; Phenomenex). Samples were analyzed by a 3 min run in negative ion mode. The mobile phases were phase A: H_2_0 and phase B: 2 mM ammonium acetate in MeOH. The gradient was 90% B for all the analysis with a flow rate of 500 µl/min. MultiQuant™ software (version 3.0.2) was used for data analysis and peak review of chromatograms.

### Statistical analysis

Experiments were performed at least in three independent biological and at least two independent technical replicates. All analysis were performed using Graph Pad Prism version 7.03 for Windows (GraphPad Software, La Jolla California USA). *P* values expressed as *P < 0.05; **P < 0.01, ***P < 0.001 were considered to be significant; in absence of indications, comparisons should be considered non-significant. Comparisons for two groups were calculated by unpaired two-tailed Student’s *t* tests and comparisons for more than two groups were calculated by one way ANOVA followed by Dunnet’s multiple comparison *post-hoc* test. Survival-curve statistical analysis was performed using the product-limit method of Kaplan and Meier (Log-rank Mantel-Cox test).

### Reporting Summary

Further information on research design is available in the Nature Research Reporting Summary linked to this article.

## Supplementary information

Supplementary Information

Peer Review

Reporting Summary

Source data

## Data Availability

A reporting summary for this Article is available as a Supplementary Information file. The datasets generated and/or analyzed in Fig. 1c, Supplementary Table 1–5 are available in the ArrayExpress repository, accession codeE-MEXP-1503, https://www.ebi.ac.uk/arrayexpress/. Source data for the figures provided are available as a Source Data file. All relevant data supporting the key findings of this study are available within the article and its Supplementary Information files or from the corresponding author upon reasonable request.
