# Supplementary material for: DNA damage and transcription stress cause ATP-mediated redesign of metabolism and potentiation of anti-oxidant buffering
Source: Nat Commun. 2019 Oct 25;10:4887. doi: 10.1038/s41467-019-12640-5 (PMC6814737; doi:10.1038/s41467-019-12640-5)
Supplement: Supplementary file 1 — Supplementary Information [file 41467_2019_12640_MOESM1_ESM.docx]

## SUPPLEMENTARY INFORMATION

**DNA damage and transcription stress cause ATP-mediated redesign of metabolism and potentiation of anti-oxidant buffering**

**Authors**

Chiara Milanese^1*^, Cíntia R. Bombardieri^1,*^, Sara Sepe^1^, Sander Barnhoorn^1^, César Payán-Goméz^1,2^, Donatella Caruso^3^, Matteo Audano^3^, Silvia Pedretti^3^, Wilbert P. Vermeij^4^, Renata M.C. Brandt^1^, Akos Gyenis^1,5^, Mirjam M. Wamelink^6^, Annelieke S. de Wit^1^, Roel C. Janssens^1^, René Leen^7^, André B. P. van Kuilenburg^7^, Nico Mitro^3^, Jan H. J. Hoeijmakers^1,4,5,8^, and Pier G. Mastroberardino^1,9^

## Supplementary figures and figure legends


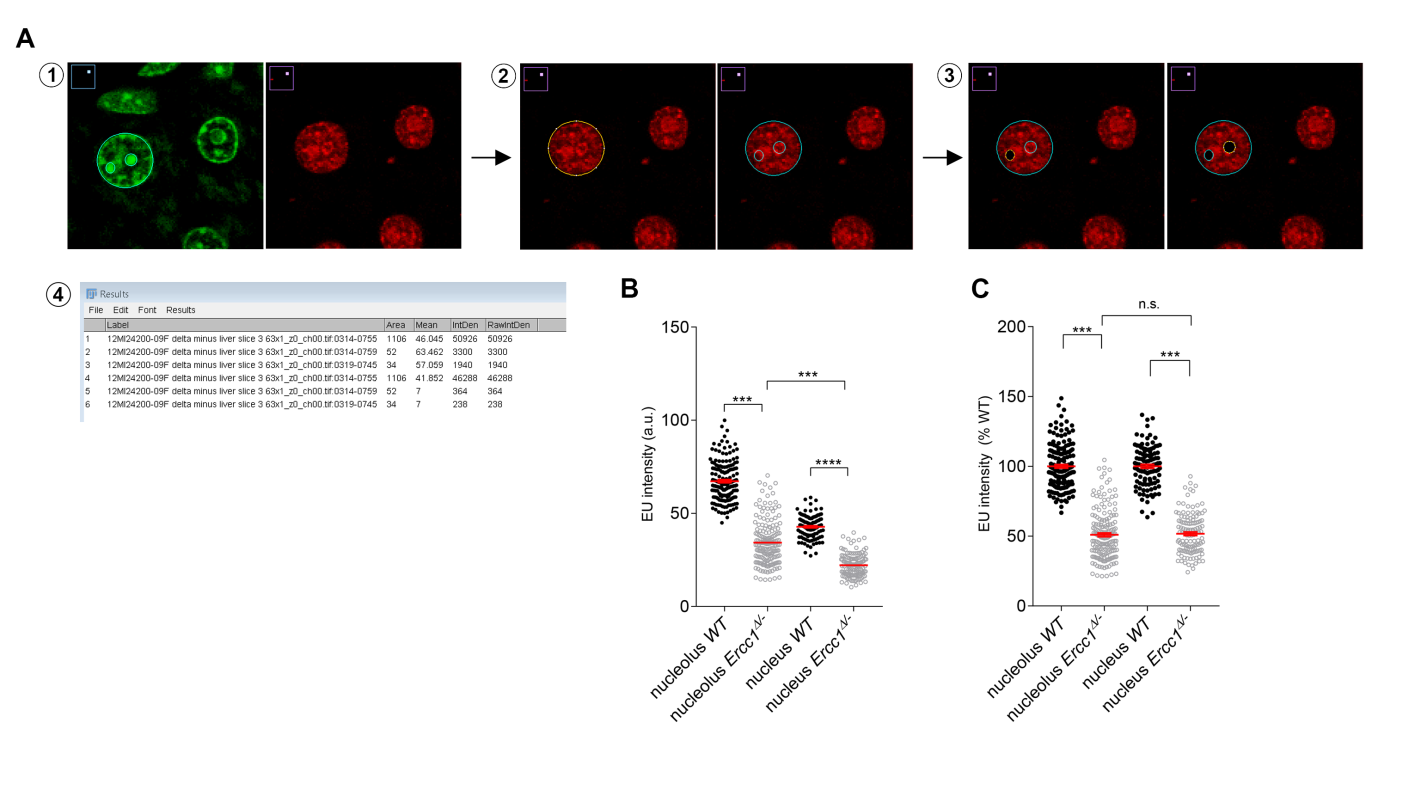


**Supplementary figure 1**. Quantification of nascent RNA synthesis in liver cells. **A.** Step by step procedure to quantify EU immunofluorescence in nuclei. Step 1: Region of interest (ROI) were designed around the nucleus and nucleoli of the cell on the green channel (SYTOX green). Step 2: ROIs were transferred from the green to the red channel (EU) and the intensity within each ROI was measured. Step3: ROIs around nucleoli were filled with background color to selectively exclude fluorescence in those areas. Step 4: Fluorescence intensity within nuclear ROI was measured. **B, C.** Dot plot graphs representing EU intensity distribution in nucleoli and nuclei from *WT* and *Ercc1^Δ/-^* liver cells. Data in B are expressed as raw mean intensity, data in C are expressed as percentage of WT mean value. n≥164 for nucleoli, n≥111 for nuclei. ***p<0.001; Student’s test.


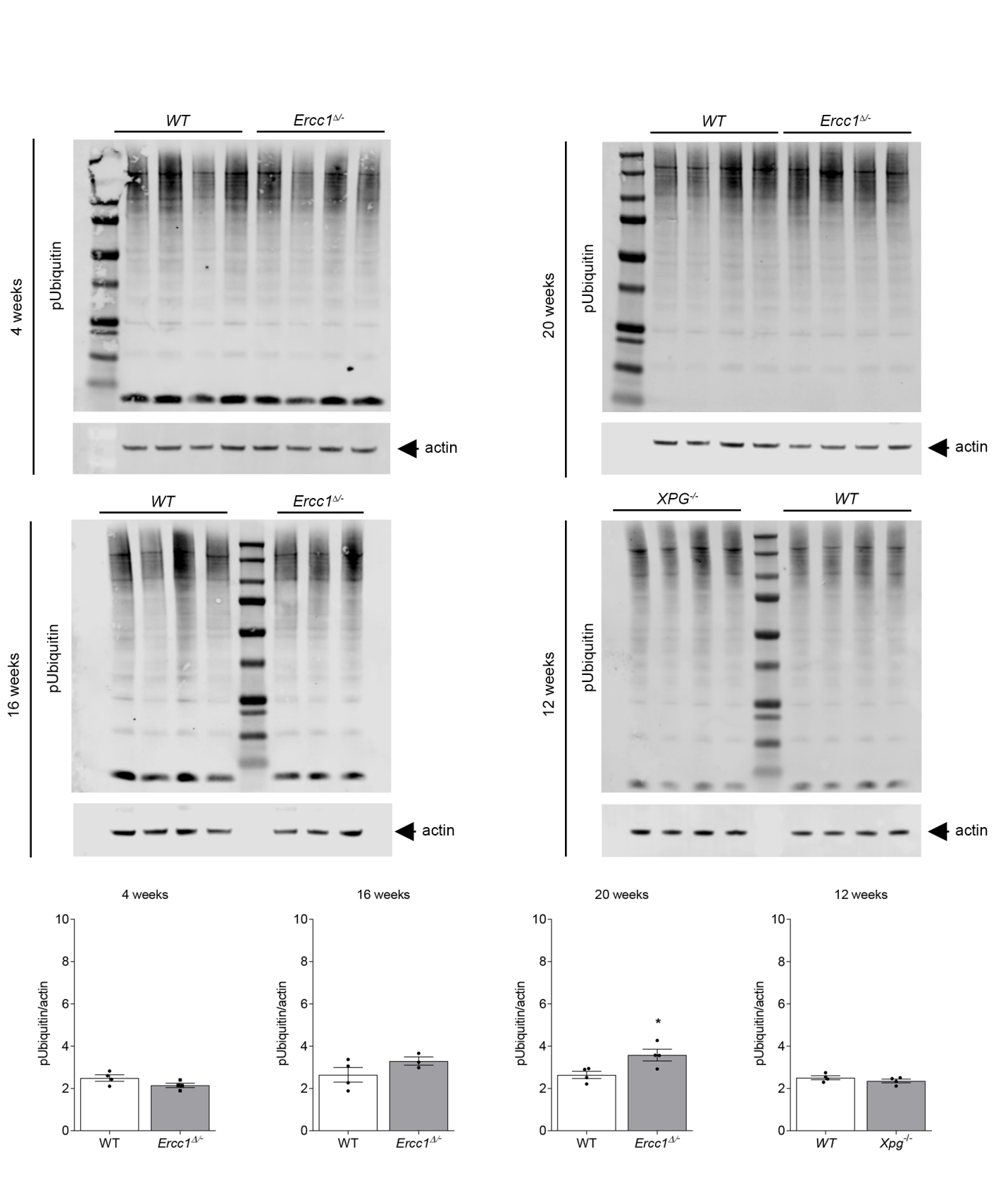


**Supplementary figure 2.** Accumulation of ubiquitinated proteins in NER defected mice. Representative images of liver protein extracts showing that protein ubiquitination is significantly increased in *Ercc1^Δ/-^* mice only at later age (20 weeks). Actin was used as loading control. Bar graphs represent mean ± s.e.m, n≥3 mice per group, *p<0.05, Student’s t-test. Images and data are provided as Source Data file.


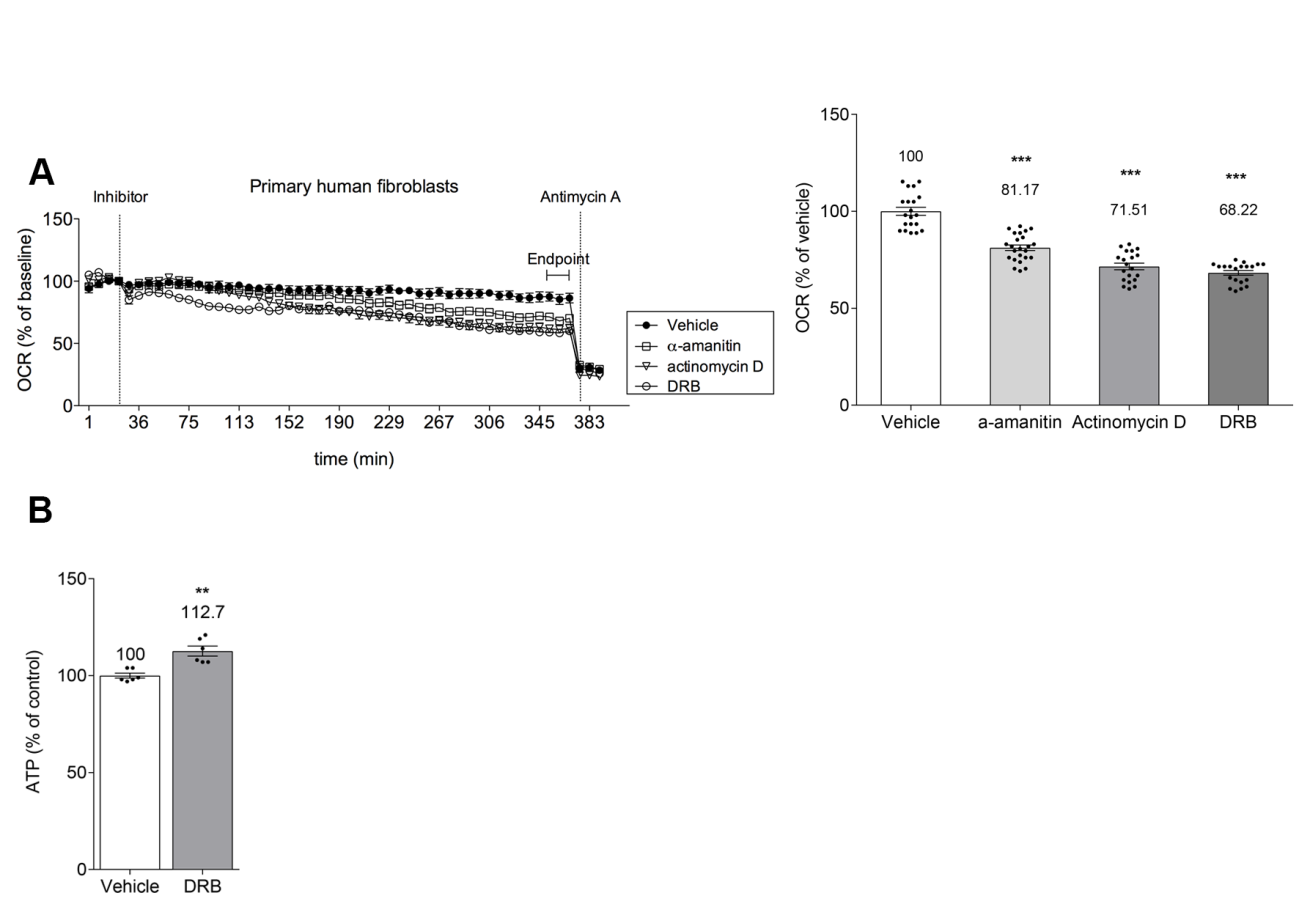


**Supplementary figure 3**. **A**. Chemical inhibition of transcription results in suppression of mitochondrial respiration, quantified at the endpoint of the respirometry analysis. **B**. Increased intracellular ATP levels in primary human fibroblasts treated for four hours with the transcription inhibitor DRB. Graph represents means ± s.e.m., n≥5, **p<0.002; Student’s t-test ; ***p<0.0001; ANOVA followed by Dunnett’s post hoc test. Data were obtained from two independent experiments. Original data are provided as a Source Data file.


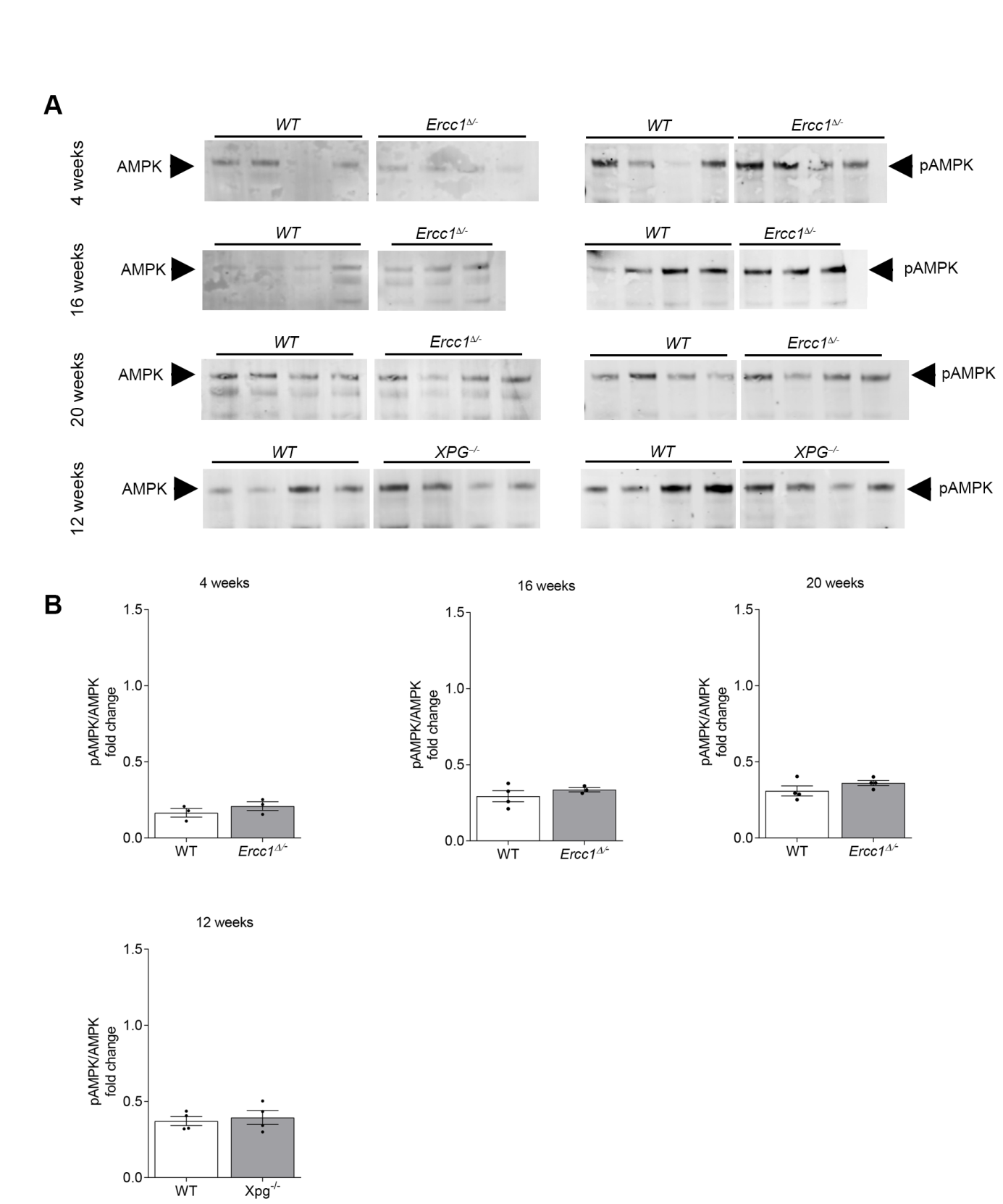


**Supplementary figure 4.** Liver accumulation of ATP in NER defective mice is not dependent on AMPK activation. Representative images of liver protein extracts showing no significant differences in AMPK phosphorylation levels between WT and NER defective mice. Bar graphs represent mean ± s.e.m, n≥3 mice per group, Student’s t-test. Images and data are provided as Source Data file.


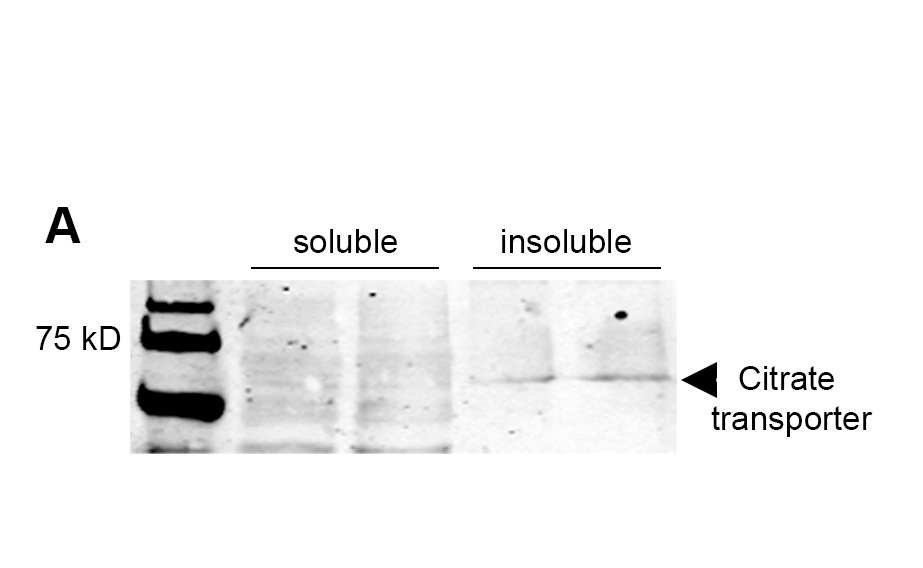


**Supplementary Figure 5**. **A**. Human fibroblasts express the membrane citrate transporter SLC13A5.

**
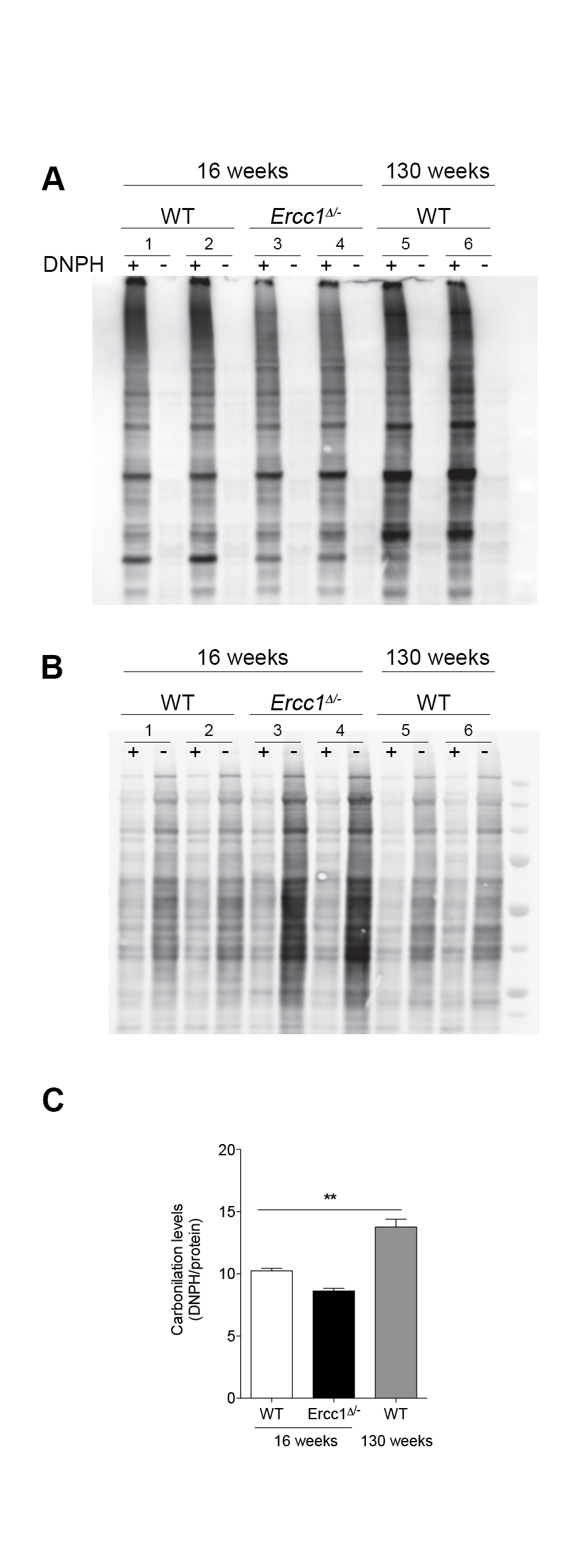

Supplementary Figure 6**. Representative immunoblot of three independent technical replicates to detect protein carbonyls derivatized with DNPH from 16-week old wild type and *Ercc1*^∆/-^ mice liver samples. **A**. Immunoblot using an anti-DNPH antibody. In the negative control (-) the DNPH reagent was omitted from the reaction. **B.** The same membrane was stained with Ponceau S to visualize protein loading and normalize DNPH signal. **C.** Carbonylation - expressed as normalized value between the DNPH and total protein signal - is decreased in mutant mice. Graph represents means ± s.e.m., n=2, **p<0.01; Student’s test. The experiment has been performed in three independent technical replicates.


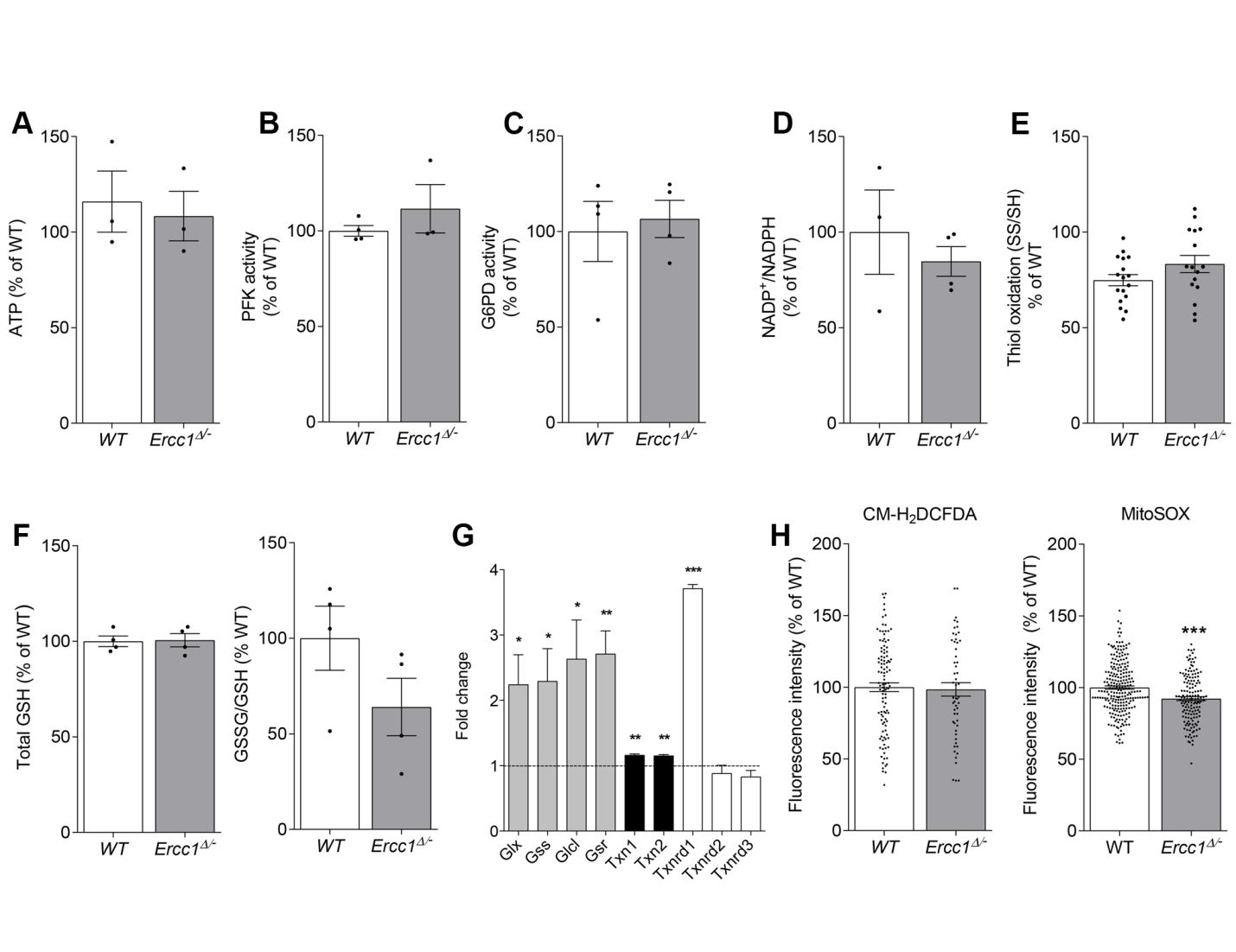


**Supplementary figure 7.** ATP-dependent metabolic redesign in *Ercc1*^Δ/-^ mice is age-dependent and does not occur in young animals. **A-C**. ATP levels, PFK and G6PD activity are unchanged in 4-week old *Ercc1*^Δ/-^ mice (n≥3 per group). Bar graphs represent mean ± s.e.m., Student’s t-test was performed. **D**-**F**. Unaltered redox state in 4-week old *Ercc1*^Δ/-^ mice as indicated by the NADP^+^/NADPH, thiol/disulfide, and GSSH/GSH redox couples. Bar graphs represent mean ± s.e.m., n≥3, Student’s t-test was performed. **G**. Transcription of genes in the GSH and Trx redox circuits in increased in 4-week old *Ercc1*^Δ/-^ livers. Bar graphs show mean ± s.e.m , n=3, * p<0.05, **p<0.01, ***p<0.001, one way ANOVA followed by Dunnett’s test**. H**. Unaltered ROS levels in freshly dissociated hepatocytes from young *Ercc1*^Δ/-^ as measured by the general ROS probe CM-H_2_DCFDA (left graph). The superoxide specific probe MitoSOX reveals a small, yet significant signal reduction in young *Ercc1*^Δ/-^ specimens (right graph). Bar graphs show mean ± s.e.m , n≥3 mice per group, ***p<0.001, Student’s t-test. Original data are provided as Source Data file.

## Supplementary tables

**Supplementary table 1.** Descriptive statistic of correlating up- and down-regulation with gene length in *Ercc1*^∆/-^ versus wild-type controls.

|  | **Number** | **Median gene length (bp)** | **Median log10 gene length (bp)** |
| --- | --- | --- | --- |
| **Total DEG** | 738 | 28374 | 4.45 |
| **Upregulated genes** | 356 | 17371 | 4.24 |
| **Downregulated genes** | 382 | 65010 | 4.81 |

**Supplementary table 2.** RNA Pol II genes expression levels in livers of *Ercc1^Δ/-^* mice at 16 weeks, as inferred from transcriptomic data. No RNA pol II subunits are down-regulated in *Ercc1^Δ/-^* mutants. In fact, the Polr2a probe-set detecting the more distal portion of the transcript (i.e. toward the 3’) even registered a higher level of transcript in mutant mice. Collectively these data clearly rule out a decrease of Pol II in *Ercc1^Δ/-^* mice.

| **Probe ID** | **Gene Symbol** | | **logFC *Ercc1^Δ/-^* vs *WT* (16 weeks)** | **P value** |
| --- | --- | --- | --- | --- |
| 1417138_s_at | *Polr2e* | 0.49 | | 0.0005 |
| 1426242_at | *Polr2a* | 0.76 | | 0.0014 |
| 1449155_at | *Polr3g* | -0.60 | | 0.0126 |
| 1439266_a_at | *Polr3k* | -0.41 | | 0.0446 |
| 1428494_a_at | *Polr2i* | -0.40 | | 0.0511 |
| 1447320_x_at | *Polr1d* | 0.17 | | 0.1062 |
| 1435057_x_at | *Polr1e* | 0.39 | | 0.1498 |
| 1416270_at | *Polr2g* | 0.22 | | 0.2360 |
| 1443466_s_at | *Polr3b* | -0.23 | | 0.2768 |
| 1451364_at | *Polr3gl* | 0.24 | | 0.2934 |
| 1424228_at | *Polr3h* | 0.11 | | 0.3323 |
| 1417720_at | *Polr2j* | 0.10 | | 0.3626 |
| 1422517_a_at | *Znrd1* | -0.13 | | 0.4108 |
| 1424258_at | *Polr2d* | 0.23 | | 0.4164 |
| 1456066_a_at | *Polr1a* | 0.10 | | 0.4410 |
| 1427402_at | *Polr2f* | 0.07 | | 0.4916 |
| 1444310_at | *Polr3a* | -0.16 | | 0.5252 |
| 1416126_at | *Polr1b* | 0.30 | | 0.5555 |
| 1429686_at | *Polr3f* | -0.18 | | 0.5722 |
| 1449648_s_at | *Polr1c* | 0.12 | | 0.5917 |
| 1416341_at | *Polr2c* | -0.09 | | 0.5993 |
| 1424565_at | *Polr3d* | 0.07 | | 0.6801 |
| 1453256_at | *Polr3c* | -0.10 | | 0.8432 |
| 1433552_a_at | *Polr2b* | -0.02 | | 0.9459 |

**Supplementary table 3. KEGG Citrate and TCA cycles .** List of genes significantly changed in the Citrate and TCA cycles of *Ercc1^Δ/-^* mice at 16 weeks. Only two genes are downregulated in the TCA pathway. Pyruvate dehydrogenase beta subunit (*Pdhb*) and cytoplasmic Acetyl-Coenzyme A Synthetase (*Acss2*) are involved in the production of acetyl-Coenzyme A for the TCA cycle and their suppression is consistent with reduced mitochondrial activity (fig. 2E in the main text).

| **ProbeID** | **Gene Symbol** | **logFC *Ercc1^Δ/-^* vs *WT* (16 weeks)** | **P value** |
| --- | --- | --- | --- |
| 1448214_at | Pdhb | -0.62 | 0.0003 |
| 1425326_at | Acly | -0.98 | 0.0004 |
| 1422479_at | Acss2 | -1.07 | 0.0013 |
| 1423644_at | Aco1 | -0.33 | 0.0027 |
| 1433984_a_at | Mdh2 | 0.34 | 0.0073 |
| 1448172_at | Mdh1 | -0.29 | 0.0107 |
| 1418560_at | Pdha1 | -0.28 | 0.0136 |
| 1418005_at | Sdhb | -0.22 | 0.0245 |
| 1425615_a_at | Pck2 | 0.40 | 0.0340 |
| 1427441_a_at | Suclg2 | -0.23 | 0.0357 |
| 1450048_a_at | Idh2 | 0.47 | 0.0360 |
| 1426688_at | Sdha | -0.24 | 0.0543 |
| 1422433_s_at | Idh1 | -0.19 | 0.0558 |
| 1447701_x_at | Idh3a | -0.25 | 0.0575 |
| 1426265_x_at | Dlat | -0.34 | 0.0600 |
| 1452206_at | Sucla2 | -0.22 | 0.0609 |
| 1422577_at | Cs | -0.31 | 0.0668 |
| 1451274_at | Ogdh | -0.20 | 0.0723 |
| 1418885_a_at | Idh3b | -0.16 | 0.1576 |
| 1423710_at | Dlst | -0.20 | 0.1672 |
| 1433263_at | Dld | 0.20 | 0.2558 |
| 1428235_at | Sdhd | -0.15 | 0.2967 |
| 1416788_a_at | Idh3g | 0.11 | 0.3569 |
| 1435986_x_at | Sdhc | -0.10 | 0.5685 |
| 1415891_at | Suclg1 | -0.05 | 0.7316 |
| 1436934_s_at | Aco2 | 0.03 | 0.8509 |
| 1450962_at | Pdha2 | -0.03 | 0.8628 |
| 1423439_at | Pck1 | -0.06 | 0.9228 |

**Supplementary table 4. KEGG Glycolysis/Gluconeogenesis.** Transcriptomic analysis of genes involved in glycolysis and gluconeogenesis (KEGG Glycolysis/Gluconeogenesis pathway). No genes are upregulated in 16-week old *Ercc1^Δ/-^* mice. Three transcripts are down-regulated genes. The only dysregulated gene strictly belonging to the glycolytic pathway was the pyruvate kinase (*Pklr*), which catalyzes the final glycolytic reaction to generate pyruvate. Transcriptional repression of the very last step of glycolysis is consistent with rerouting of glucose in upstream reactions as detected in the multiple convergent experiments presented in this study. The other two down-regulated genes are involved in glucose storage and particularly in gluconeogenesis (G6pc, i.e. glucose 6-phosphatase) and in glycogen formation (Gck, i.e glucokinase).

| **Probe ID** | **Gene Symbol** | **logFC *Ercc1^Δ/-^* vs *WT* (16 weeks)** | **P value** |
| --- | --- | --- | --- |
| 1419146_a_at | Gck | -2.27 | 3.10E-07 |
| 1421258_a_at | Pklr | -0.93 | 0.0001 |
| 1448214_at | Pdhb | -0.62 | 0.0003 |
| 1448470_at | Fbp1 | -0.31 | 0.0032 |
| 1437398_a_at | Aldh9a1 | -0.48 | 0.0100 |
| 1418560_at | Pdha1 | -0.28 | 0.0136 |
| 1417880_at | G6pc | -1.77 | 0.0229 |
| 1425615_a_at | Pck2 | 0.40 | 0.0340 |
| 1426265_x_at | Dlat | -0.34 | 0.0600 |
| 1415902_at | Aldh7a1 | -0.27 | 0.0679 |
| 1419737_a_at | Ldha | 0.20 | 0.0882 |
| 1455235_x_at | Ldhb | 0.40 | 0.1334 |
| 1451149_at | Pgm2 | -0.17 | 0.1813 |
| 1437974_a_at | Hk1 | 0.22 | 0.1887 |
| 1422070_at | Adh4 | 0.37 | 0.2133 |
| 1416069_at | Pfkp | 0.22 | 0.2225 |
| 1433263_at | Dld | 0.20 | 0.2558 |
| 1439435_x_at | Pgk1 | 0.11 | 0.2853 |
| 1423529_at | G6pc2 | -0.13 | 0.3198 |
| 1421058_at | Adh7 | 0.18 | 0.3542 |
| 1418373_at | Pgam2 | 0.10 | 0.3576 |
| 1452582_at | Galm | -0.13 | 0.3633 |
| 1426554_a_at | Pgam1 | 0.12 | 0.3956 |
| 1419023_x_at | Eno1 | -0.12 | 0.4126 |
| 1415865_s_at | Bpgm | -0.25 | 0.4142 |
| 1453283_at | Pgm1 | 0.20 | 0.4297 |
| 1452927_x_at | Tpi1 | 0.09 | 0.4341 |
| 1437583_x_at | Acss1 | 0.12 | 0.4614 |
| 1448789_at | Aldh1a3 | 0.09 | 0.4801 |
| 1416780_at | Pfkm | -0.10 | 0.4894 |
| 1451260_at | Aldh1b1 | 0.28 | 0.5108 |
| 1434987_at | Aldh2 | 0.07 | 0.5383 |
| 1416185_a_at | Adh5 | 0.08 | 0.6107 |
| 1434247_at | Ldhal6b | 0.08 | 0.6603 |
| 1415776_at | Aldh3a2 | -0.28 | 0.7198 |
| 1449088_at | Fbp2 | 0.10 | 0.7280 |
| 1422612_at | Hk2 | 0.05 | 0.7324 |
| 1417951_at | Eno3 | 0.06 | 0.7628 |
| 1416784_at | Pgk2 | 0.05 | 0.7747 |
| 1451194_at | Aldob | 0.03 | 0.8005 |
| 1415847_at | Ldhc | -0.05 | 0.8158 |
| 1418829_a_at | Eno2 | -0.04 | 0.8179 |
| 1450962_at | Pdha2 | -0.03 | 0.8628 |
| 1442798_x_at | Hk3 | 0.04 | 0.8650 |
| 1418752_at | Aldh3a1 | 0.02 | 0.8680 |

**Supplementary table 5. KEGG Pentose Phosphate Pathway.** List of genes in the PPP pathway in the *Ercc1^Δ/-^* mice at 16 weeks.

| **Probe ID** | **Gene Symbol** | **logFC *Ercc1^Δ/-^* vs *WT* (16 weeks)** | **P value** |
| --- | --- | --- | --- |
| 1451015_at | *Tkt* | -0.56 | 0.0003 |
| 1424047_at | *Dera* | -0.40 | 0.0021 |
| 1448470_at | *Fbp1* | -0.31 | 0.0032 |
| 1416705_at | *Rpe* | -0.53 | 0.0040 |
| 1424840_at | *Rbks* | -0.36 | 0.0053 |
| 1454843_at | *Prps2* | -0.42 | 0.0163 |
| 1452145_at | *H6pd* | 0.26 | 0.1259 |
| 1416052_at | *Prps1* | 0.23 | 0.1552 |
| 1451149_at | *Pgm2* | -0.17 | 0.1813 |
| 1425129_a_at | *Taldo1* | -0.13 | 0.1919 |
| 1416069_at | *Pfkp* | 0.22 | 0.2225 |
| 1453283_at | *Pgm1* | 0.20 | 0.4297 |
| 1430434_at | *Tktl2* | 0.09 | 0.4409 |
| 1416780_at | *Pfkm* | -0.10 | 0.4894 |
| 1449567_at | *Tktl1* | -0.11 | 0.6419 |
| 1436771_x_at | *Pgd* | 0.10 | 0.7123 |
| 1449088_at | *Fbp2* | 0.10 | 0.7280 |
| 1418337_at | *Rpia* | -0.06 | 0.7840 |
| 1453203_at | *Prps1l1* | -0.05 | 0.7857 |
| 1451194_at | *Aldob* | 0.03 | 0.8005 |
| 1453541_at | *Pfkl* | -0.04 | 0.8705 |

**Supplementary table 6.** Abbreviation used in figure 3 for the enzymes participating to the glycolytic and the pentose phosphate pathways.

| \| **Symbol** \| **Enzyme** \| \| --- \| --- \| \| HK1 \| Hexokinase-1 \| \| GPI \| Glucose-6-phosphate isomerase \| \| PFK \| 6-phosphofuctokinase \| \| ALDO \| Fructose-bisphosphate aldolase \| \| TPI \| Triosephosphate isomerase \| \| G6PD \| Glucose-6-phosphate 1-dehydrogenase \| \| GNL \| Gluconolactonase \| \| PGD \| 6 -phosphogluconate dehydrogenase \| \| RPIA \| Ribose-5-phosphate isomerase \| \| RPE \| Ribulose-phosphate 3-epimerase \| \| TKT \| Transketolase \| \| TALDO \| Transaldolase \| |
| --- | --- | --- | --- | --- | --- | --- | --- | --- | --- | --- | --- | --- | --- | --- | --- | --- | --- | --- | --- | --- | --- | --- | --- | --- | --- | --- |
|  |

**Supplementary table 7.** List and sequences of the primers used in this study.

| **Species** | **Gene symbol** | **Gene name** | **Primer forward (5’-3’)** | **Primer reverse (5’-3’)** |
| --- | --- | --- | --- | --- |
| Mus musculus | ***Hk 1*** | hexokinase 1 | CTCCTTACGTGGGGAACGTG | CCAGAGCAGGAGAATGACGG |
| Mus musculus | ***Pfkl*** | phosphofructokinase | TCCGCACCTACAACATCCAC | GGCTGGGATGACACACATGA |
| Mus musculus | ***Glut-1*** | Glucose transporter 1 | AGTGTATCCTGTTGCCCTTCT | CATCGGCTGTCCCTCGAAGC |
| Mus musculus | ***Glut-3*** | Glucose transporter 3 | GACTGCTTCTGAGTGCTGCTA | CATTGGCGATCTGGTCAACC |
| Mus musculus | ***Gclc*** | glutamate-cysteine ligase, catalytic subunit | AACACAGACCCAACCCAGAG | TGGCACATTGATGACAACCT |
| Mus musculus | ***Gsr*** | glutathione reductase | CACGACCATGATTCCAGATG | CAGCATAGACGCCTTTGACA |
| Mus musculus | ***Txn1*** | thioredoxin 1 | TGGATCCATTTCCATCTGGT | GCCTCCTGAAAAGCTTCCTT |
| Mus musculus | ***Txn2*** | thioredoxin 2 | CTTGTTGCCATTCCCCTAGA | CAGAGGCAGGAGAGGAAATG |
| Mus musculus | ***Txnrd1*** | thioredoxin reductase 1 | TGTCACACCGACTCCTCTTG | TCCGTCATTTTCTCCCAGTC |
| Mus musculus | ***Txnrd2*** | thioredoxin reductase 2 | GCTTCTGGCAAGGAAGACAC | CCCTCAGCAACATCTCCAAT |
| Mus musculus | ***Txnrd3*** | thioredoxin reductase 3 | TTGCTGGCAGAGACAACAAC | CGTGATTTCACCAGCATTTG |
| Mus musculus | ***Gss*** | glutathione synthetase | AGCCCTGAAACAGATCGAGA | CATAGAGCTCCCAGGCTTTG |
| Mus musculus | ***Glrx*** | glutaredoxin | AGTTGATGTGCATCGCAGAG | GGGCAAATTGCAACAGTTTT |
