# Supplementary material for: DNA damage and transcription stress cause ATP-mediated redesign of metabolism and potentiation of anti-oxidant buffering
Source: Nat Commun. 2019 Oct 25;10:4887. doi: 10.1038/s41467-019-12640-5 (PMC6814737; doi:10.1038/s41467-019-12640-5)
Supplement: Supplementary file 2 — Peer Review [file 41467_2019_12640_MOESM2_ESM.pdf]

Reviewers' comments:

Reviewer #1 (Remarks to the Author):

In this Milanese et al. paper, the authors describe how the presence of transcription-blocking lesions, primarily but not exclusively, in an ERCC1Δ/- context, induces a DNA damage dependent change of cellular metabolism. In particular, the authors propose that because of the presence of transcription-blocking lesions, there is an accumulation of ATP in cells, as ATP is not anymore needed for transcription. ATP allosterically inhibits the PFK enzyme inhibiting the glycolisis pathway favouring consequently the pentose phosphate pathway. This change in metabolic pathways alters the redox metabolism in the cells ending up generating reductive stress. This paper builds on a series of high profile publications that over the last decade have showed how in contexts with defective nucleotide excision repair there is an alteration of the metabolism. In this sense, although the actual findings presented in the paper are new and add an important level of details to the field, the whole concept that DNA damage accumulation/persistence has an impact on the choice of metabolic pathways is not that novel. Importantly, while the change in the metabolic pathway choice in the cells in clear and convincing, there are however some major concerns I have with this paper.
Throughout the paper the authors refer to the presence of transcription-blocking lesions and/or transcription stress as the reason that reduces the transcription levels in an ERCC1Δ/- context, leading to the accumulation of ATP in the cells. However, neither in this paper, nor in any of the cited papers, there is a direct evidence of the presence of transcription stress or transcription stalling lesions in untreated conditions. There is indeed evidence that there are higher levels of DNA damage present in these cells, and ERCC1 plays a role in multiple DNA damage repair pathways and not only NER as highlighted by the authors, that will potentially hinder on cells ability of performing transcription; but there is no direct evidence that this is due to the presence of transcription stalling lesions because of an inability of completing NER. This is for me however a crucial point that the authors should address, also in light of data produced by several labs over the years (Egly and Svejstrup to mention a couple), that show that NER factors, including ERCC1, have also endogenous roles in transcription regulation independent from their role in NER. Therefore, is the reduced transcription observed in the ERCC1 mutant mice due to the accumulation and persistence of transcription-stalling lesions, or is it due to a defect in transcribing specific genes independently of the presence of lesions? In other words, is the progressive phenotype due to an increased accumulation of DNA damage in the cells, or a progressive inability to express genes because of the intrinsic role of ERCC1 in transcription regulation? This is a crucial point to me that needs to be addressed considering the general statements done in this paper.
If there are transcription stalling lesions one prediction is that there is an increase of stalled/paused RNA Pol II, that we know will be consequently ubiquitilated and degraded: is there increased ubiquitilated RNA Pol II in these ERCC1 mutant cells? And if so, is the reduction in transcription levels associated with a progressive reduction of total RNA Pol II levels in cells while they age? This would directly link the phenotype observed in ERCC1 mutant mice with its direct role in NER rather than a more generic role in transcription regulation. This because there is also the possibility that the transcription defect could depend on reduced mRNA expression of the actual RNA Pol II subunits. Mouse Polr2a is a 24.64 kb long gene, but Polr2b is almost 40kb, so its expression could be reduced in a transcription defective context leading to a reduction of RNA Pol II levels in the cells and reduced transcription output, unrelated to the fact that the cells may have or not transcription-stalling lesions. This can be easily assessed by real time PCR of the mRNA of RNA Pol II subunits in the aging mice.
Related to the transcription deregulation in ERCC1 mutant cells, there is the conclusion in the paper that the phenotype observed is directly and specifically dependent on the accumulation of ATP in the cells because of the lack of transcription. However, in the Schumacher et al., 2008 paper cited by the authors, NER defective including ERCC1 mutants present changes in transcription levels of many metabolic pathways, including deregulations in glycolysis genes expression. Although the expression of many metabolic factors is not affected, there are also some that change in the paper, and again, is this due to the presence of DNA damage-induced transcription stress, or simply changes in the transcriptome? The fact that transcription inhibition via DRB shows similar changes to ATP levels and metabolic changes to those observed in ERCC1 mutant cells, to me it does not solve completely this

point, as DRB leads to an accumulation of RNA Pol II in the proximity of the TSS, that although showing a similar result in terms of EU output, is intrinsically different to the presence of the DNA damage in cells.
There are then some specific points on which I would like to raise authors' attention:
- Fig1: the authors should prove that the reduced progressive transcription output is dependent on increasing DNA damage in the cells (gH2AX foci for example) that leads to transcription stalling, hence increased levels of ubiquitilated RNA Pol II and/or reduced RNA Pol II levels in cells upon aging, as mentioned above.
- Fig1C: the authors present the average mean gene length for transcripts that are up- and down-regulated in ERCC1 mutant cells. However, average means on population distributions are biased by outliers and populations are better described by average medians. Indeed, for their t-test the authors use Mann-Whitney that is a non-parametric t-test that compares medians rather than means, so please correct.
- Fig2: in the legend the authors state that "Graphs represent the typical results of at least three independent biological replicates", does this mean that the graphs are from a "representative" experiment or the average of multiple biological replicates? This needs to be stated clearly. Moreover, there is throughout all the figure legends the lack of important details that need to be added, like the number of replicates (N) and whether the showed plots with SEM are from technical or biological replicates, as these are not specified in the material and methods section either.
- Fig3 legend, reorder F after E.
- Fig3E: are the quantifications from multiple biological replicates on cells extracted from the same mouse, or from multiple mice? Please specify in the legend.
- Fig4H: the authors should present also a western blot for GSH and not only the quantification, as consistency within the same figure with panel I.
- Fig4L: in the legend there are 6 curves but only 4 are visible in the plot, are the missing ones completely overlapping or are they not present in the graph? It would be important mentioning this in the figure legend.
- Lines 166-172: in the text, the authors go from describing Fig3 to figure 4C and then back to Fig3E; this is a bit confusing for the reader, so please reorder.
- Line 203: the paragraph title is "Manipulation of transcription levels…" but there is nothing in the paragraph about it, please change title.
- Line 208: "human" instead of "mouse" fibroblasts.
- Line 241: the authors should provide some description of where this mutation is, just to give and idea of how severe this mutation is for protein activity.
- Line 454: which is the GSH antibody used? It is not described
- Reporting summary: the authors have ticked the boxes for the first two points ("The exact sample size (n) for each experimental group/condition, given as a discrete number and unit of measurement" and "An indication of whether measurements were taken from distinct samples or whether the same sample was measured repeatedly") but this is not done consistently throughout the paper and as previously raised, these are missing in the figure legends for many results.

Reviewer #2 (Remarks to the Author):

This manuscript by Milanese et al. explores relationships between transcription-coupled repair (TCR) and metabolic reprogramming, using TCR-defective mouse strains and mouse and human cell lines. The authors find that ATP levels are increased in TCR mutants, impairing glycolysis and routing glucose-derived carbon into the pentose phosphate shunt to generate reducing equivalents.

A prior body of work, much of it by one of the co-authors (Dr. Hoeijmakers) has documented a cyto-protective response occurring in cells with defective TCR. This manuscript adds to this literature, and provides new potential mechanistic insight, by attempting to link defective TCR with altered metabolism. In principle it should be of interest to the readership of Nature Communications.

However, there are important aspects of the models presented that remain unproven and speculative, and moreover elements of the manuscript are somewhat descriptive in nature. Specifically:

1. Based on their decreased transcription (preferentially of long transcripts), the authors speculate that translation, an intensively energy-requiring process, may be decreased in TCR mutants. The authors should measure bulk translation in their cells, and perhaps block it chemically to see whether this also elevates cellular ATP levels.
2. AMPK is the major cellular ATP sensor, and very sensitive to the ATP/ADP ratio. The authors should test p-AMPK in their models to further document the physiologic impact of the perturbations of ATP levels.
3. The alterations of intermediates in the pentose phosphate pathway in ERCC1-mutant cells (Fig. 3F) are intriguing. Does directly inducing DNA damage produce similar changes in levels of these metabolites?
4. Altered cellular redox would affect the NAD/NADH ratio, and accumulation of NADH would be predicted to impair glycolysis and mitochondrial respiration, apart from the mechanisms proposed. The authors should explore this idea. Other investigators have leveraged proteins that can directly oxidize NAD in this context (e.g. Ndip1).
5. The authors should show levels of all glycolytic and TCA cycle enzyme mRNAs someplace in the manuscript, not simply the few shown in Fig. 2H. An enzyme that is not rate-limiting in normal cells might become so if levels of pathway enzymes are sufficiently perturbed.
6. How were the doses of NAC and rotenone used in mice (Fig. 4L) arrived at? Can the authors document that they exerted on-target biological effects using these dosing regimens, particularly for rotenone? Also, 2 of the WT curves (vehicle and rotenone) appear to be missing from the figure.
7. Stylistically, it would be reassuring to the reviewer if all bar graphs in the manuscript were presented as scatterplots.

Reviewer #3 (Remarks to the Author):

Overall evaluation
Milanese, Bombardieri et al. present a work in which a relevant connexion between nuclear and cytosolic events is highlighted. We assist in recent times to the emergence of links between previously unconnected fields, and the research as the one presented in this paper contributes to creating awareness of the importance that these relationships are unmasked, characterized and known by the scientific community. The rationale of the work is well stated, the experimental flow is well designed and explained, the experimental execution is in most cases accurate and the overall message is important. In general terms, I think this research should be released to the community and would be of interest and of inspiration to instigate further research on these novel grounds. At this stage, however, in my opinion, there are relevant concerns, some experimental and some conceptual, that need to be either further reinforced or substantially remodelled.

Major concerns (conceptually)
- There is a strong bias, already from the introduction and in the very first paragraph of results, driving the concept towards DNA lesions being at the basis of the random, stochastic transcription halt that, subsequently, remodels ATP metabolism. While very possibly true, I estimate that the real heart of the message is that ANY transcription halt would entail the same outcome. The proof is provided by the authors themselves, when they recapitulate the same impact by using up to three different transcription inhibitors. I understand the interest of the authors in wanting to put forward that DNA damage is at the origin of the phenomenon, for this has implications in itself, but the ercc1 deficiency is just a “tool” to promote the transcriptional halt, as could have been a defective RNA pol II mutation, for example. Moreover, they insist enormously on the accumulation of unrepaired lesions being the major cause for transcriptional decline. Yet, it is well established that the TFIIH complex will abandon promoters and then abrogate new transcription events AT THE LEVEL OF INITIATION because of its recruitment to NER sites (Vélez-Cruz, Egly 2012 PNAS), where it can furthermore be retained,

worsening the problem of not entailing new initiation rounds (Herrera-Moyano 2014 PLOS Genetics). While this is a massive phenomenon in the face of highly NER-demanding lesions such as UV, it will occur though more modestly under situations as that recreated by Ercc1 deficiency, then contributing to lowering the transcriptional output. Moreover, other types of DNA damages also have the potential of impacting transcription in a yet different manner, as for example the local inhibition of transcription surrounding DNA double strand breaks. These possibilities should be considered in the rationale of the manuscript.

- The Figure Legends are, all of them, an extension of results but do not provide the information about the experiments technically talking. Is it a Western blot? Is it an enzymatic measure? What do the acronyms mean? What is exactly being quantified? How long was a particular treatment applied? Why soluble and insoluble fractions are shown?... I understand that the title of a Figure Legend can recapitulate the main message provided by the figure. But the rest of it cannot become an extension of the Results section itself. The Figure Legends in this paper explain or even add information scheduled for the Results section. For example, Figure 2G, "however, maximal glycolytic capacity after oligomycin injection is retained". Yet nothing is said about this in the main text, where it belongs. Moreover, in several instances, there are panels that are not even mentioned in the Figure Legend (see for an example Sup.Fig4). Last, the phenomenon is so strong that there are panels where, since the result is negative, the panel is not even mentioned in the Figure Legend!! (see NADP/NADPH ratio in the description of Figure 7E-G). I raise this as a major concern for I think a deep, full re-writing of all Figure Legends is mandatory.
- The Results section concerning "glucose rerouting and redox alterations manifest with aging" is poorly supported and even contradictory. The authors "question whether the metabolic changes observed progress with age". When I read this I expect to see kinetic studies, seeing how transcriptional decline correlates with redox remodelling. Yet, the authors provide the experiments done with younger (4-weeks-old) specimens and, since no alterations are significantly detected yet, invoke an aging-associated effect. In my opinion, these data constitute a Supplementary Figure when characterizing the phenomenon in 16-weeks-old animals, with a comment on it; yet do not provide solid proof to justify a whole Results section. Moreover, even if the process of aging coupled to DNA damage accumulation is accelerated in ercc1 mice, it is still expectable in WT specimens at later times of their lives. This makes me turn my attention to Supp Fig 4a, where the cabonylation levels in WT proteins at 130 weeks are clearly more elevated than at 16 weeks. This agrees with a higher oxidative status and is contrary to the message the authors want to provide. I would therefore be careful about putting too much emphasis on this direction, which nevertheless is not necessary for the relevance of the main message.

Major concerns (experimentally)

- The authors focus on RNA polymerase II transcription to support their claims. Yet, Pol I transcription accounts for the higher proportion of the cell bulk transcription (as deduced when noticing its efficient shut off after dietary restriction or rapamycin treatment), and is the most suited machine to promote significant alterations susceptible of effectively "saving resources", as is the scope of this work. Moreover, the central NER player TFIIH is now formally demonstrated as a relevant player in promoting efficient RNA polymerase I transcription elongation, as revealed by the work from the lab of G. Giglia-Mari and others. Thus, in the context of this work, the impact, extent and importance contributed by RNA polymerase I transcription should be assessed. For example, in Figure 1A and more even in Figure 1B, the strong, remaining EU signals are very much reminiscent of nucleoli. Are they so? Can the authors simultaneously hybridize for a nucleoli marker and split their EU signals into those contributed by nucleoli transcription (mostly Pol I-dependent) and the rest? Would this add something new to their conclusions? Second, can the authors envision a way to (at least to a great extent) inhibit specifically RNA Pol I and assess some of their metabolic readouts?
- In referring to Figure 1B, the authors sharply claim that the heterogeneity seen in EU incorporation is a proof of the stochasticity of the DNA damage-triggered transcription halt. Other options are possible and therefore this should just be put as an option, a suggestion. Yet, there is one very straightforward one, that is, that the cells may be markedly in different phases of the cell cycle because of the ercc1 deficiency, then bearing different transcriptional activities. A cytometry

measurement here, if unchanged, would help the authors suggest (yet not claim), their purpose.
- One main claim of the paper is that the metabolic remodelling tethers an increase in the levels of NADPH. While this is central to support one of the main paper messages, the measurements in search of increased NADPH were performed only in two conditions (ercc1 and xpg mutants), and only found altered as expected in the first case. This part needs therefore to be reinforced. I would suggest repeating the measurements when inhibiting transcription pharmacologically for a short time, for example. In this same line, it is also very striking that, while the transcription decline in the xpg mutant is dramatic, much stronger than in the ercc1 animals, still the NADPH measurement does not fulfil the predictions. I do not think this invalidates the main notion, but I think that the authors should find an experimental approach to try to reconcile this apparent paradox. Is the NADPH being redirected to something else in the xpg mouse that prevents us from seeing it?
- In the same line, the authors do not find a significant increase in G6PD activity when trying to induce metabolism remodelling using externally provided A. Why would this be? Can the authors try a way / a condition to prove this point? Would it be possible to overcome this "negative result" by scoring an alternative readout, for example, that the PPP products are indeed exacerbated?
- The model of the authors predicts that a hyper-active, boosted transcription would deplete ATP and lead to a situation where an oxidative redox status would predominate. This is in agreement with our notion of, for example, cancer cells growth. While not being critical, if a system (even a non-mouse system) could be used to suddenly boost transcription and evaluate the key parameters the authors measure, this would beautifully reinforce their model.
- The notion that derives from the paper that a too reduced environment is deleterious for the cell is of high relevance. We tend to read and hear that a highly oxidative environment is harmful. We are less used to the opposite notion. On these grounds, it would be better for the impact and the consideration of the paper to probe for a molecular process being negatively affected by the reducing environment. For example, I imagine that protein folding within the Endoplasmic Reticulum would enormously suffer from this. In this case, the cells will activate the Unfolded protein response (UPR). There is a high degree of correlation between genome instability and ER stress, yet little molecular connexions. If the authors manage to validate a transcriptional activation of the UPR and/or an expansion of the ER folding compartment by microscopy, for example, not only will they reinforce the physiological consequences of the altered redox state but also will provide a connexion for these, otherwise, only correlated events in the bibliography.

Minor concerns (conceptually)
- At a point in the Discussion the authors write "a key role of the PPP in the DNA damage response". The DNA Damage Response, the DDR, responds to a well-established term to designate the cascade of sensing, transmitting and reacting to DNA lesions in the DNA, which is orchestrated by apical kinases such as ATM and ATR, and effectors such as Chk2 and Chk1. Given the existence of such a network, with which the present work does not cope, I suggest to simply change this and other similar sentences of the paper by "the response to DNA damage". It is very minor change but, conceptually, it changes a lot.
- All the Oxygen consumption rates curves (as in Figure 2E, for example), the drugs used at each step, why, what they do, what this evaluates, etc, please explain widely and with details in the main Results text, it is greatly under-explained even for experts, therefore imagine for those who are not.
- The event of polyploidization is not apparent or even understood for the non-specialist. A quantification (if possible), a better visual representation and a better explanation would be appreciated.
- The affirmation that "protein carbonyls are a footprint of RS" needs to be justified by a reference.
- There is a small, yet important raise in the number of works assessing the reciprocal impact of metabolic alterations and genome status. It is important to give visibility to these works, for they constitute the basis for a more open attitude towards this research. The authors make reference to the work by Vermeij et al, 2016. The paragraph in the discussion "DNA damage and glucose metabolism" would be a good place to enlarge the view about this issue. I encourage the authors to make a more extensive search and dissertation around this theme. For example the short Mol. Cell by Brian Luke's lab discussing genome fidelity maintenance and dietary restriction-like treatments would be a good

starting point.
- The authors discuss briefly that the increased activation of G6PD can be mediated by the DNA damage response protein ATM. This observation, which matches so beautifully with their observation, deserves, in my view, a bit more development. How does the kinetics of this phenomenon compare to the one the authors describe here? We have the general idea that the transcriptional response is fast, and that the DNA damage response mediated by proteins such as ATM is even faster. But the allosteric regulation proposed in this work would be even "more" faster. Are these timings compatible with what is described?
- Measurement of glycolysis as indicated by medium acidification requires using a culturing medium that IS NOT BUFFERED (Mekhail Lee 2006 Cell Cycle). Yet, authors claim in the methods section that they used a medium containing 100 mM sodium pyruvate at pH 7. Is this perhaps relevant to the accuracy of the measurements?
- It is not intuitive to understand why the short genes would be UPREGULATED in the ercc1 mutants. Since the reason why the ercc1 mutants display decreased general transcription is not so critical for the message of the paper, could this transcriptome analysis be given less importance in the main paper flow?

Minor concerns (experimentally)
- Quantification of EU in Figure 1A is not satisfactory. Authors do not use the same representation than in Figure 1B, which is much fairer, for raw EU intensity (even in arbitrary units) is provided. The way shown in Figure 1A prevents us from seeing the actual evolution of transcription activity in the WT, and does not allow us to see the individual values for different cells of the population. This is a loss, for perhaps two populations may be apparent, one more affected than other. I propose to display one representative experiment of the actual population of cells with all the raw values. To see the reproducibility of the experiment, other experiments can be provided with the same format in Supplementary.
- Given the strong heterogeneity observed in EU incorporation in the experiment presented in Figure 1B and Sup Fig 1, one would expect that the quantification yields two population of points, which is not the case. Is there a mistake in this quantification? And in the same line as above, could authors provide the same quantification for the biological replicates of this experiment in supplementary figures?
- How sure are we that the used drugs (i.e. transcription inhibitors) do not affect mitochondrial performance? Can the authors comment on this?
- The authors claim "genes of key glycolytic enzymes are very compact, thus relatively unaffected by the DNA damage-induced decline": this is not shown by any analysis.
- The panels from Supp Figure 1 would be appreciated already in the main Figure 1, to have a better overview of the cell population.
- The WB for GSH level in Figure 4H needs to be shown!
- The survival curves in Figure 4L need to be much bigger.
- The y-axes and the titles in Figure 3E cannot be read.
- The amount of carbonylated proteins in Ercc1 mutants at 16 weeks is decreased compared to the WT, yet the difference is not significant. I get it from the Figure that the experiment was done only twice? Could this be reinforced?

I congratulate the authors for this interesting, necessary and inspiring work and encourage them with the eventual revision of the manuscript, for this will be a beautiful contribution to science.

We would like to thank the reviewers for the careful and insightful comments. We certainly believe that addressing the referees' criticism greatly improved our study.

Point-to-point response to the reviewers

(the original reviewers' comments are in blue)

Changes in the main text are highlighted in yellow

Reviewer #1 (Remarks to the Author):

In this Milanese et al. paper, the authors describe how the presence of transcription-blocking lesions, primarily but not exclusively, in an ERCC1Δ/- context, induces a DNA damage dependent change of cellular metabolism. In particular, the authors propose that because of the presence of transcription-blocking lesions, there is an accumulation of ATP in cells, as ATP is not anymore needed for transcription. ATP allosterically inhibits the PFK enzyme inhibiting the glycolisis pathway favouring consequently the pentose phosphate pathway. This change in metabolic pathways alters the redox metabolism in the cells ending up generating reductive stress. This paper builds on a series of high profile publications that over the last decade have showed how in contexts with defective nucleotide excision repair there is an alteration of the metabolism. In this sense, although the actual findings presented in the paper are new and add an important level of details to the field, the wholeconcept that DNA damage accumulation/persistence has an impact on the choice of metabolic pathways is not that novel. Importantly, while the change in the metabolic pathway choice in the cells in clear and convincing, there are however some major concerns I have with this paper.

We appreciate that the reviewer finds our data on metabolic rewiring, which constitute the object of the study, clear and convincing. We would also like to take the chance to emphasize that the concept of transcription stress as a primary cause of metabolic redesign via a mechanisms involving ATP levels and activating antioxidant defenses is unprecedented - at least to our best knowledge – and thus highly innovative. Besides

unraveling important molecular insights, these findings have major clinical implications e.g. for stress resistance and ageing.

Throughout the paper the authors refer to the presence of transcription-blocking lesions and/or transcription stress as the reason that reduces the transcription levels in an ERCC1Δ/- context, leading to the accumulation of ATP in the cells. However, neither in this paper, nor in any of the cited papers, there is a direct evidence of the presence of transcription stress or transcription stalling lesions in untreated conditions. There is indeed evidence that there are higher levels of DNA damage present in these cells, and ERCC1 plays a role in multiple DNA damage repair pathways and not only NER as highlighted by the authors, that will potentially hinder on cells ability of performing transcription; but there is no direct evidence that this is due to the presence of transcription stalling lesions because of an inability of completing NER. This is for me however a crucial point that the authors should address, also in light of data produced by several labs over the years (Egly and Svejstrup to mention a couple), that show that NER factors, including ERCC1, have also endogenous roles in transcription regulation independent from their role in NER. Therefore, is the reduced transcription observed in the ERCC1 mutant mice due to the accumulation and persistence of transcription-stalling lesions, or is it due to a defect in transcribing specific genes independently of the presence of lesions? In other words, is the progressive phenotype due to an increased accumulation of DNA damage in the cells, or a progressive inability to express genes because of the intrinsic role of ERCC1 in transcription regulation? This is a crucial point to me that needs to be addressed considering the general statements done in this paper.

We appreciate the reviewer's comments and we fully agree that this manuscript does not provide direct proof that DNA damage causes the dramatic transcription decline in the liver of NER-defective organisms. However, the expert will agree that direct demonstration of DNA damage is unfortunately extremely difficult from the technical standpoint. In fact no reliable, accurate, and fully reproducible methods have been developed to measure physiological levels of spontaneous DNA lesions, with the only exception of cyclopurines. These lesions have been already reported to be elevated in liver of *Ercc1*

mouse mutants (Wang, Clauson et al. 2012) and, to our best knowledge, this analysis can be reliably performed only in one very specialized lab. Nonetheless, we would like to offer the following arguments favoring the concept that RNA synthesis collapse in *Ercc1* (and *Xpg*) mutant mice liver is attributable to the repair function of these proteins, and thus to DNA damage. First, the drastic decline of the overall transcriptional output, which is up to 90% at the age of 20 weeks (see figure 1A), is difficult to reconcile with any transcription factor that controls expression of a specific subset of genes. A specific transcription factor is not expected to reduce overall transcription to only 10% of wt. Second, also the progressive nature of the decline with age (4, 16 and 20 weeks, see figure 1A) is hard to explain as a consequence of the lack of a specific transcription factor (e.g. *Ercc1*); its absence would exert an immediate rather than a progressive effect - over a timespan of months - on the transcription of genes controlled by the target promoter. Third, our observation that transcription suppression is gene length-dependent (see fig. 1C) would be unexpected as hitherto, and to our best knowledge, no transcription factor has been described to regulate genes on the base of their length. Fourth, the transcriptional stress occurs with normal aging, but is exaggerated in transcription-coupled repair mutants. Moreover, others (Andrade-Lima, Veloso et al. 2015) and we see the same gene-length decline when cultured cells are exposed to actual transcription-blocking UV DNA damage in a dose-dependent manner, indicating causality. Additionally, this gene-length dependent transcription decline is permanent when TCR is defective (Andrade-Lima, Veloso et al. 2015), consistently with our data in *Ercc1* and *Xpg* mutants. All these arguments strongly favor time-dependent accumulation of DNA damage over the transcription function of ERCC1 as the principal cause of transcription stress; however, as the referee correctly points out, these findings do not unequivocally prove the causality of DNA damage in transcription decline. Therefore, we have now included the above arguments to the manuscript, performed and added new experiments as requested by the referee (see below) and toned down this aspect of transcriptional stress (see changes in the manuscript). Nonetheless, as also the referee indicates, whether DNA damage is the main cause of transcription stress is not the main message of the manuscript, which deals with the molecular mechanism of metabolic redesign and its effect on cellular protection.

If there are transcription stalling lesions one prediction is that there is an increase of stalled/paused RNA Pol II, that we know will be consequently ubiquitilated and degraded: is there increased ubiquitilated RNA Pol II in these ERCC1 mutant cells? And if so, is the reduction in transcription levels associated with a progressive reduction of total RNA Pol II levels in cells while they age? This would directly link the phenotype observed in ERCC1 mutant mice with its direct role in NER rather than a more generic role in transcription regulation. This because there is also the possibility that the transcription defect could depend on reduced mRNA expression of the actual RNA Pol II subunits.
Mouse Polr2a is a 24.64 kb long gene, but Polr2b is almost 40kb, so its expression could be reduced in a transcription defective context leading to a reduction of RNA Pol II levels in the cells and reduced transcription output, unrelated to the fact that the cells may have or not transcription-stalling lesions. This can be easily assessed by real time PCR of the mRNA of RNA Pol II subunits in the aging mice.

To address the reviewer's concerns, we investigated the RNA pol II (RPB1) expression at mRNA and protein level, as well as its phosphorylation status in *Ercc1*$^{\Delta/-}$ mice of different ages.
Despite the very strong reduction in RNA synthesis in *Ercc1* liver that progresses with age, we did not detect significant changes in RNA pol II largest subunit, RPB1 at the protein level, when compared with wt controls. Also comparison between *Ercc1* mutants of different ages failed to reveal changes in RPB1 (please see fig.1D). Consistent with this finding, analysis of mRNA levels also failed to indicate a down-regulation of RNA pol II subunits (please see supplementary table 2), even using an *Polr2a* probe-set detecting the more distal portion of the transcript (i.e. toward the 3').
We also investigated RNA pol II phosphorylation on Ser2 and Ser5 in its C-terminal repeat domain. Also in this case we failed to detect major alterations (please see fig. 1D). Collectively, these data indicate that transcription decline in *Ercc1*$^{\Delta/-}$ mice is not caused by decreased RNA pol II levels. In fact, the observation that elongating Ser2-P RNA polymerase II remains constant, or even increases with age (see fig 1D) despite the

dramatic collapse of overall transcriptional output rather indicates that a large proportion of the enzyme is non-productive, consistently with the concept that RNA pol II is stalled by DNA damage.

Finally, we explored whether transcription decline in *Ercc1*$^{\Delta/-}$ is associated with increased ubiquitination of RNA Pol II as suggested by the referee. Immunoblot analysis following immunocapture of ubiquitinated proteins, however, failed to reveal any signal for RNA Pol II (please see fig. 1E). The absence of detectable ubiquitination of RNA pol II is in agreement with the absence of reduced total RNA pol II levels (Fig. 1D) and indicates that the stalled RNA polymerases are not rapidly degraded.

These new data are now included in the main text (please see the last paragraph in the results section entitled *Defective NER causes progressive decline of transcription*). We thank the referee for raising these points, which strengthen and extend the manuscript.

Detailed analysis of transcription stress in normal aging using full genome nascent RNA seq, ChIP seq for total RNA pol II, Serine 2 (elongating) and Serine 5 (initiating) phosphorylated RNA pol II in liver of normal aging is the topic of another manuscript that is in a final stage of preparation. This extensive paper rules out gene-specific regulation and e.g. methylation or altered chromatin structure as underlying mechanisms and identifies DNA damage as the -by far- most likely cause of the genome-wide progressive decline of transcriptional output, which we now find to be a new feature of ageing in post-mitotic tissues in metazoans (Gyenis, Chang, Baar, Barnhoorn, Brandt, Vermeij, Tresini, Hoeijmakers and Pothof, manuscript close to submission). Obviously, our findings do not rule out an additional role of Ercc1 and other NER factors in transcription regulation.

Related to the transcription deregulation in ERCC1 mutant cells, there is the conclusion in the paper that the phenotype observed is directly and specifically dependent on the accumulation of ATP in the cells because of the lack of transcription. However, in the Schumacher et al., 2008 paper cited by the authors, NER defective including ERCC1 mutants present changes in transcription levels of many metabolic pathways, including deregulations in glycolysis genes expression. Although the expression of many metabolic factors is not affected, there are also some that change in the paper, and again, is this due to the presence of DNA damage-induced transcription stress, or simply chang-

es in the transcriptome? The fact that transcription inhibition via DRB shows similar changes to ATP levels and metabolic changes to those observed in ERCC1 mutant cells, to me it does not solve completely this point, as DRB leads to an accumulation of RNA Pol II in the proximity of the TSS, that although showing a similar result in terms of EU output, is intrinsically different to the presence of the DNA damage in cells.

We believe that the changes in transcription observed in our TCR mutants are the result of both gene expression changes associated with ageing as well as the gene length dependent transcriptional stress, described first in Vermeij et al., 2016 (Vermeij, Dolle et al. 2016). In this regard it is interesting to note that one of the most prominent and important changes observed before, the attenuation of the IGF1 somatotropic axis (Schumacher, van der Pluijm et al. 2008) might be related to the considerable size of the IGF1 gene itself, which is 75 kb and its long size is conserved in numerous species. Accumulation of DNA damage in this gene might directly contribute to the suppression of somatotropic, which in turn has an effect on many other cellular pathways (Gyenis, Chang, Baar, Barnhoorn, Brandt, Vermeij, Tresini, Hoeijmakers and Pothof, manuscript close to submission).

As correctly indicated by the reviewer, the experiments with DRB serve as a further demonstration that persistent block of transcription leads to ATP imbalance and metabolic redesign. We agree that the mechanism by which DRB and DNA damage act on transcription are very different. The purpose of this experiment was to investigate whether decline in transcription *per se* increases ATP levels which consequently might drive metabolic redesign. Again, we believe that unraveling the precise mechanisms responsible for transcription decline falls behind the scope of the present work. We emphasized this in the text and toned down our claims on DNA damage and transcription.

There are then some specific points on which I would like to raise authors' attention:

- Fig1: the authors should prove that the reduced progressive transcription output is dependent on increasing DNA damage in the cells (gH2AX foci for example) that leads to

transcription stalling, hence increased levels of ubiquitilated RNA Pol II and/or reduced RNA Pol II levels in cells upon aging, as mentioned above.

We appreciate the suggestion of the reviewer. The referee is correct that nucleotide excision repair (NER) and e.g. UV damage can induce gH2AX foci not only in replicating cells, but even in non-S-phase cells, which are likely induced by incised repair intermediates in an ATR-dependent manner (see e.g. (Hanasoge and Ljungman 2007)). However, the vast majority of these foci is derived from global genome NER, while the contribution of transcription-coupled repair (TCR) is very small (see Hanasoge et al., cited above). Moreover, our TCR/NER mutants are not able to complete the incision step the repair cascade, so they fail to show these gH2AX foci.
As discussed above, we believe that transcription decline is caused by lesion-stalled RNA polymerases, for which there is no evidence that they are associated with DNA breaks and/or gH2AX. Since most cells in the aging *Ercc1* mutant liver are post-mitotic, no S-phase derived gH2AX foci can be expected. Hence, the analysis of gH2AX in the *Ercc1* liver is not unequivocal and unfortunately not very informative. The issues of DNA damage as the cause of the transcription stress and ubiquitilated RNA polymerase II have been addressed above. .

- Fig1C: the authors present the average mean gene length for transcripts that are up- and down-regulated in ERCC1 mutant cells. However, average means on population distributions are biased by outliers and populations are better described by average medians. Indeed, for their t-test the authors use Mann-Whitney that is a non-parametric t-test that compares medians rather than means, so please correct.

We are grateful to the referee for this suggestion, which has been taken care of.

- Fig2: in the legend the authors state that “Graphs represent the typical results of at least three independent biological replicates”, does this mean that the graphs are from a “representative” experiment or the average of multiple biological replicates? This needs to be stated clearly. Moreover, there is throughout all the figure legends the lack of im-

portant details that need to be added, like the number of replicates (N) and whether the showed plots with SEM are from technical or biological replicates, as these are not specified in the material and methods section either.

This point has been further clarified in the revised version (please see end of online methods section) and the correct n number for each graph has been incorporated in the figure legends.

- Fig3 legend, reorder F after E.

We thank the referee for this suggestion. Panels have been reordered.

- Fig3E: are the quantifications from multiple biological replicates on cells extracted from the same mouse, or from multiple mice? Please specify in the legend.

This information has been now specified in the figures' legends.

- Fig4H: the authors should present also a western blot for GSH and not only the quantification, as consistency within the same figure with panel I.

We apologize for the lack of clarity; GSH was measured using a well-established spectrophotometric assay. In the revised version of the manuscript, this has been explained in greater detail in both the methods (please see the section entitled *Glutathione measurement*) and in the figure legend.

- Fig4L: in the legend there are 6 curves but only 4 are visible in the plot, are the missing ones completely overlapping or are they not present in the graph? It would be important mentioning this in the figure legend.

Thank you for the remark. The overlapping curves have been specified in the legend.

- Lines 166-172: in the text, the authors go from describing Fig3 to figure 4C and then back to Fig3E; this is a bit confusing for the reader, so please reorder.

This has been done.

- Line 203: the paragraph title is “Manipulation of transcription levels…” but there is nothing in the paragraph about it, please change title.

The title has now been corrected: “*Manipulation of intracellular ATP concentration is sufficient to elicit metabolic redesign*”

- Line 208: “human” instead of “mouse” fibroblasts.

This has been done.

- Line 241: the authors should provide some description of where this mutation is, just to give and idea of how severe this mutation is for protein activity.

The patient (96RD235) has a deletion of a deoxycytidine in the codon coding for Arg45, causing a frameshift. This information has been added in the text, in the results section.

- Line 454: which is the GSH antibody used? It is not described

This analysis does not involve the use of antibodies and, as described in the methods (please see the section entitled *Determination of thiol- disulfide redox equilibrium*), relies on the direct derivatization of oxidized and reduced cysteines (the authors described the method in a previous article, please see {Horowitz, 2011 #51}). We apologize for the lack of clarity; this issue has been now explained in greater detail in both the main text (please see second paragraph in the results' section entitled *Metabolic redesign in Ercc1*$^{\Delta/-}$ *culminates in an abnormal reduction of the cellular redox state*) and in the figures' legends.

- Reporting summary: the authors have ticked the boxes for the first two points (“The exact sample size (n) for each experimental group/condition, given as a discrete number and unit of measurement” and “An indication of whether measurements were taken from distinct samples or whether the same sample was measured repeatedly”) but this is not done consistently throughout the paper and as previously raised, these are missing in the figure legends for many results.

We thank the reviewer for this and all previous remarks, which have led to a substantially more complete and convincing manuscript.

Reviewer #2 (Remarks to the Author):

This manuscript by Milanese et al. explores relationships between transcription-coupled repair (TCR) and metabolic reprogramming, using TCR-defective mouse strains and mouse and human cell lines. The authors find that ATP levels are increased in TCR mutants, impairing glycolysis and routing glucose-derived carbon into the pentose phosphate shunt to generate reducing equivalents.

A prior body of work, much of it by one of the co-authors (Dr. Hoeijmakers) has documented a cyto-protective response occurring in cells with defective TCR. This manuscript adds to this literature, and provides new potential mechanistic insight, by attempting to link defective TCR with altered metabolism. In principle it should be of interest to the readership of Nature Communications. However, there are important aspects of the models presented that remain unproven and speculative, and moreover elements of the manuscript are somewhat descriptive in nature. Specifically:

1. Based on their decreased transcription (preferentially of long transcripts), the authors speculate that translation, an intensively energy-requiring process, may be decreased in

TCR mutants. The authors should measure bulk translation in their cells, and perhaps block it chemically to see whether this also elevates cellular ATP levels.

This is a very interesting suggestion and we fully agree that this next level of exploration is highly important. Despite the fact that various biological buffering systems are known to operate, one expects that a decline of 90% of overall transcriptional output (as in the case of the liver of 20 week-old Ercc1 mutant mice, see Fig 1A) will not be without effects on translation and many other cellular processes. We believe, this would be a perfect explanation for the recent interesting discovery of increased loss of stoichiometry for numerous protein complexes including the ribosome in multiple organs with ageing in various species (Cellerino A. and Ori A, unpublished results) and the observation that transcript levels and protein abundance become progressively decoupled e.g. in chronological aging in the primate brain ((Wei, Hu et al. 2015); see also (Cellerino and Ori 2017)). Thus, this is a very promising direction following transcriptional stress, which we would like to embark upon for an in-dept investigation including ribosome profiling, mass spec analysis and metabolic labeling. However, this requires a new cohort of ageing *Ercc1* mutant mice and for this we are in the process of applying to get permission from the animal ethical committee, which unfortunately is a very lengthy procedure that takes half a year or more. Therefore, the interesting studies suggested by the referee are certainly with high priority on our to-do list to be pursued in the future but they are not within the realm of the current manuscript.

2. AMPK is the major cellular ATP sensor, and very sensitive to the ATP/ADP ratio. The authors should test p-AMPK in their models to further document the physiologic impact of the perturbations of ATP levels.

As per request of the reviewer, we have evaluated AMPK phosphorylation in NER deficient models. The expert is correct when stating that AMPK is very sensitive to the ATP/ADP ratio. We would like to emphasize, however, that AMPK activation is a consequence of ATP depletion rather than surplus and its phosphorylation leads to a potentiation of ATP-producing pathways and/or a reduction of pathways with high bioenergetics

demand. It is therefore not surprising that pAMPK levels do not change in NER models, which feature a surplus of ATP. These considerations have been now added to the main text (please see beginning of the second paragraph in the results section entitled *Decreased transcription leads to elevated ATP pool*) and the data have been in included in supplementary figure 4.

3. The alterations of intermediates in the pentose phosphate pathway in ERCC1-mutant cells (Fig. 3F) are intriguing. Does directly inducing DNA damage produce similar changes in levels of these metabolites?

The reviewer raises a very interesting question, which unfortunately does not have a straightforward answer. From our studies it is apparent that only DNA damage chronically interfering with transcription will trigger transcriptional stress, which in turn leads to activation of the pentose-phosphate shunt. This means that only specific types of DNA damage (UV, Illudin S) chronically applied over an extended period of time will be able to mimic the situation in the liver of *Ercc1* mice upon aging. Moreover, the cells should be in non-replicating cell state, because replication complexes will release blocked RNA polymerases, explaining why transcription stress is found in largely post-mitotic tissues. These conditions are difficult to realize *in vitro*. An additional unknown and possibly confounding factor stems from the bioenergetics demand of DNA repair; DNA damage repair and the DNA damage response are very energy demanding in terms of signaling (phosphorylation of 1000-nds protein sites), DNA re-synthesis, repair protein turn-over, and activation of e.g. PARP1 for poly-ADP-ribosylation of target proteins. In this scenario, reduced ATP use because of interrupted transcription could be paralleled by augmented ATP hydrolysis, which is necessary to fuel the DNA repair machinery and the DNA damage response. The net effects of these divergent bioenergetics dynamics on overall ATP balance is therefore unpredictable.

4. Altered cellular redox would affect the NAD/NADH ratio, and accumulation of NADH would be predicted to impair glycolysis and mitochondrial respiration, apart from the

mechanisms proposed. The authors should explore this idea. Other investigators have leveraged proteins that can directly oxidize NAD in this context (e.g. Ndip1).

We are afraid we do not fully understand the experiment suggested by the reviewer. Generally speaking, NAD and NADP have different primary roles in the cell: NADP is principally used as an electron donor in anabolic pathways, and is therefore maintained in a reduced state, while NAD is predominantly used as an electron acceptor in catabolic pathways, and is therefore maintained in an oxidized state (Pollak, Dölle et al. 2007; Sun, Dai et al. 2012). Hence, these couples are at different equilibria and redox changes in one couple does not necessarily imply changes in the other (in any case, different redox couples are generally at non-equilibrium, please see (Kemp, Go et al. 2008)). Moreover, excess of NADH would likely increase mitochondrial respiration because of increased substrate availability (i.e. NADH) for complex I and the latter possibility is in contrast with our data showing reduced rotenone sensitive respiration (i.e. attributable to complex I) in *Ercc1* mutants (please see figure 2F). On these grounds, we respectfully suggest that analyzing the NAD/NADH ratio would not be particularly informative for the purposes of the present study.

5. The authors should show levels of all glycolytic and TCA cycle enzyme mRNAs someplace in the manuscript, not simply the few shown in Fig. 2H. An enzyme that is not rate-limiting in normal cells might become so if levels of pathway enzymes are sufficiently perturbed.

As per request of the reviewer, we extracted this information from the transcriptomic data. When we analyzed the genes in the glycolysis/gluconeogenesis pathways and the TCR pathway (i.e. KEGG_GLYCOLYSIS_GLUCONEOGENESIS and KEGG_CITRATE_CYCLE), we found only six differently regulated genes. While we could not detect any upregulated gene, we identified five down-regulated genes. The only dysregulated gene belonging to the glycolytic pathway was the pyruvate kinase (*Pklr*), which catalyzes the final glycolytic reaction to generate pyruvate. Transcriptional repression of the very last step of glycolysis is consistent with rerouting of glucose in

upstream reactions as detected in the multiple convergent experiments presented in this study. Pyruvate dehydrogenase beta subunit (*Pdhb*) and cytoplasmic Acetyl-Coenzyme A Synthetase (*Acss2*) are involved in the production of acetyl-CoenzymeA for the TCA cycle; ATP-citrate synthase (*Acly*) functions in the entry step of the TCA cycle and catalyzes the condensation of oxaloacetate and Acetyl-CoenzymeA to generate citrate. Overall, downregulation of these genes is consistent with both mitochondrial anomalies (i.e. diminished reserve capacity and rotenone-sensitive respiration) observed in *Ercc1* mutants, as well as diminished glycolysis and consequent reduction in availability of pyruvate, that is the main substrate for acetyl-CoenzymeA formation. The other two downregulated genes are involved in glucose storage and particularly in gluconeogenesis (*G6pc*, i.e. glucose 6-phosphatase) and in glycogen formation (*Gck*, i.e glucokinase). This information has now been added in the supplementary material (please see supplementary table 3,4,5) and in the main text (please see last paragraph of the results section entitled *Decreased transcription leads to elevated ATP pool*).
Additionally, we could detect no differences in expression of genes of the Pentose Phosphate Pathway (i.e. KEGG_PENTOSE_PHOSPHATE_PATHWAY) (please see supplementary table 5 and end of the last paragraph in the results section entitled *Alterations in the ATP pool cause redesign of carbohydrate fluxes*). Collectively, these data strongly support allosteric rerouting of metabolism.

6. How were the doses of NAC and rotenone used in mice (Fig. 4L) arrived at? Can the authors document that they exerted on-target biological effects using these dosing regimens, particularly for rotenone? Also, 2 of the WT curves (vehicle and rotenone) appear to be missing from the figure.

The doses were selected on the basis of previous literature. For instance, Reyes and colleagues (Reyes, Cittolin-Santos et al. 2016) used NAC at a concentration of 25mg/mL. Clark and co-workers (Clark, Clore et al. 2010) used 6.5mg/mL for several months. Collectively, these elements indicate that the range of concentration we used falls within the doses normally used for *in vivo* studies in mice.

As far as rotenone is concerned, we do acknowledge that its potential off-target effects are a spiny issue, which we discussed in a previous article while recommending careful titration of the dosage (Greenamyre, Cannon et al. 2010). In this study, we took as a starting point the work of Pan-Montojo and colleagues (Pan-Montojo, Anichtchik et al. 2010); here, a dose of 5mg/kg principally affected the gastrointestinal tract, therefore minimizing systemic, wide-spread toxicity. 5mg/kg correspond to 0.125 mg for an average mouse with 25 grams body weight.

In our study, we decided to use a slightly lower dosage than what reported by Pan-Montojo and co-workers in light of the fragile phenotype of *Ercc1* mutants and of their general sensitivity. We therefore administered 10mg/mL rotenone in drinking water, which approximately corresponds to 0.04mg per mouse per day given that mice (including *Ercc1* mutants) drink approximately 4 mL of water daily (please see http://web.jhu.edu/animalcare/procedures/mouse.html).

These considerations and the related articles have now been added to the methods section.

7. Stylistically, it would be reassuring to the reviewer if all bar graphs in the manuscript were presented as scatterplots.

We have changed some of the graphs in a scattered dot plot format. However, we believe that the representation of the panels is crisper and more intelligible in the form of bar graphs. To reassure the reviewer about the data, we would like to emphasize that all the results are included in the Source Data file.

Reviewer #3 (Remarks to the Author):

Overall evaluationMilanese, Bombardieri et al. present a work in which a relevant connexion between nuclear and cytosolic events is highlighted. We assist in recent times to the emergence of links between previously unconnected fields, and the research as the one presented in this paper contributes to creating awareness of the importance that

these relationships are unmasked, characterized and known by the scientific community. The rationale of the work is well stated, the experimental flow is well designed and explained, the experimental execution is in most cases accurate and the overall message is important. In general terms, I think this research should be released to the community and would be of interest and of inspiration to instigate further research on these novel grounds. At this stage, however, in my opinion, there are relevant concerns, some experimental and some conceptual, that need to be either further reinforced or substantially remodelled.

Major concerns (conceptually)

- There is a strong bias, already from the introduction and in the very first paragraph of results, driving the concept towards DNA lesions being at the basis of the random, stochastic transcription halt that, subsequently, remodels ATP metabolism. While very possibly true, I estimate that the real heart of the message is that ANY transcription halt would entail the same outcome. The proof is provided by the authors themselves, when they recapitulate the same impact by using up to three different transcription inhibitors. I understand the interest of the authors in wanting to put forward that DNA damage is at the origin of the phenomenon, for this has implications in itself, but the ercc1 deficiency is just a “tool” to promote the transcriptional halt, as could have been a defective RNA pol II mutation, for example. Moreover, they insist enormously on the accumulation of unrepaired lesions being the major cause for transcriptional decline. Yet, it is well established that the TFIIH complex will abandon promoters and then abrogate new transcription events AT THE LEVEL OF INITIATION because of its recruitment to NER sites (Vélez-Cruz, Egly 2012 PNAS), where it can furthermore be retained, worsening the problem of not entailing new initiation rounds (Herrera-Moyano 2014 PLOS Genetics). While this is a massive phenomenon in the face of highly NER-demanding lesions such as UV, it will occur though more modestly under situations as that recreated by Ercc1 deficiency, then contributing to lowering the transcriptional output. Moreover, other types of DNA damages also have the potential of impacting transcription in a yet different manner, as for example the local inhibition of transcription surrounding DNA double

strand breaks. These possibilities should be considered in the rationale of the manuscript.

We thank the reviewer for the careful, articulated, and insightful feedback. We do agree that, in principle, any persistent transcription halt would lead to the observed metabolic alterations, and fully agree that this manuscript does not provide direct proof that DNA damage causes the dramatic transcription decline in the liver of NER-defective organisms. Direct demonstration of DNA damage is, unfortunately, technically extremely difficult; thus far, no reliable, accurate methods have been developed to measure physiological levels of spontaneous DNA lesions, with the exception of cyclopurines, which indeed have been reported to be elevated in liver of *Ercc1* mouse mutants and which can be measured only in one highly specialized laboratory. Because cyclopurines are indeed capable of transcription stalling, they are certainly good candidates for at least part of the transcription problems. However, as the referee correctly points out, TFIIH is simultaneously implicated in transcription initiation and its availability for this task is influenced by the extent of involvement in NER/TCR, as we have previously shown in living cells and even living mice (see (Hoogstraten, Nigg et al. 2002; Giglia-Mari, Miquel et al. 2006; Giglia-Mari, Theil et al. 2009)). Recruitment of TFIIH from transcription initiation to DNA repair will only constitute a problem for sudden high levels of DNA damage (e.g. after a high UV dose), but will not play a major role under normal conditions with low physiological levels of spontaneous DNA damage.

In addition, we have the following arguments that favor DNA damage to be mainly responsible for the collapse of RNA synthesis in the liver of *Ercc1* and *Xpg* mutant mice. First, the magnitude of the decline of the overall transcriptional output which is up to 90% at the age of 20 weeks (see figure 1A). It would be hard to imagine that spontaneous DNA levels would constantly recruit the vast cellular amount of TFIIH, which is estimated to be present in 110.000 complexes per mammalian cell (Kimura, Tao et al. 1999; Hoogstraten, Nigg et al. 2002). Secondly, the progressive nature of the decline with age (4, 16 and 20 weeks, see figure 1A) is also hard to explain as a consequence of increasing demands on TFIIH, which would limit transcription initiation to 10% of normal conditions. Third, our observation that transcription stress is gene length-

dependent with long genes preferentially being suppressed (see fig. 1C).This would be unexpected as TFIIH would have to know the length of the gene in advance when deciding whether or not to initiate transcription at a specific promoter. Fourth, as requested by referee 1 we have added experiments to measure levels of total RNA pol II, as well as of phosphorylated Ser2- (elongating) and Ser5- (initiating) RNA pol II; these analyses did not detect any reduction despite the observed high transcription stress, therefore providing arguments that many elongating RNA polymerases may be indeed stalled (see Figure 1D and 1E and accompanying text in the manuscript).
All these elements strongly favor time-dependent accumulation of DNA damage as the principal cause of transcription stress, but - as the referee correctly points out – our data do not unequivocally demonstrate a causative role of DNA damage. Therefore, we have toned down this aspect of the transcriptional stress (see changes in the manuscript). That said, we would like to reiterate that whether DNA damage is the main cause of transcription stress is not the main message of the manuscript, which deals with the molecular mechanism of metabolic redesign and its effect on cellular protection.

- The Figure Legends are, all of them, an extension of results but do not provide the information about the experiments technically talking. Is it a Western blot? Is it an enzymatic measure? What do the acronyms mean? What is exactly being quantified? How long was a particular treatment applied? Why soluble and insoluble fractions are shown?... I understand that the title of a Figure Legend can recapitulate the main message provided by the figure. But the rest of it cannot become an extension of the Results section itself. The Figure Legends in this paper explain or even add information scheduled for the Results section. For example, Figure 2G, "however, maximal glycolytic capacity after oligomycin injection is retained". Yet nothing is said about this in the main text, where it belongs. Moreover, in several instances, there are panels that are not even mentioned in the Figure Legend (see for an example Sup.Fig4). Last, the phenomenon is so strong that there are panels where, since the result is negative, the panel is not even mentioned in the Figure Legend!! (see NADP/NADPH ratio in the descrip-

tion of Figure 7E-G). I raise this as a major concern for I think a deep, full re-writing of all Figure Legends is mandatory.

We apologize for the confusion. Figure legends have been re-written in the revised version of the manuscript and additional relevant information has been added in the results section.

- The Results section concerning “glucose rerouting and redox alterations manifest with aging” is poorly supported and even contradictory. The authors “question whether the metabolic changes observed progress with age”. When I read this I expect to see kinetic studies, seeing how transcriptional decline correlates with redox remodelling. Yet, the authors provide the experiments done with younger (4-weeks-old) specimens and, since no alterations are significantly detected yet, invoke an aging-associated effect. In my opinion, these data constitute a Supplementary Figure when characterizing the phenomenon in 16-weeks-old animals, with a comment on it; yet do not provide solid proof to justify a whole Results section. Moreover, even if the process of aging coupled to DNA damage accumulation is accelerated in ercc1 mice, it is still expectable in WT specimens at later times of their lives. This makes me turn my attention to Supp Fig 4a, where the cabonylation levels in WT proteins at 130 weeks are clearly more elevated than at 16 weeks. This agrees with a higher oxidative status and is contrary to the message the authors want to provide. I would therefore be careful about putting too much emphasis on this direction, which nevertheless is not necessary for the relevance of the main message.

As per request of the reviewer, we have now moved this figure in the supplementary material (please see supplementary figure 7). Moreover, in the revised version of the manuscript the paragraph has been incorporated in the results section entitled *Manipulation of intracellular ATP concentration is sufficient to elicit metabolic redesign*. We would like to emphasize, however, that these experiments address an important issue: defects in the glycolytic pathway are typically associated with inborn metabolic disorders

that manifest at very early stages of life. Here, we wanted to sort out whether this was also the case for NER mutants, or if the defects were rather developing in time.
That said, we do agree with the reviewer that in this manuscript is unnecessary to speculate on the affinities between *Ercc1* mutants and natural aging. However, it may also be plausible that the effects observed in NER mutants may have some relevance for aging, especially because transcription decline occurs in natural aging, and DNA damage accumulation is a hallmark of aging. Obviously, the question on whether in natural aging these phenomena reach a sufficient extent to cause ATP imbalance, glucose rerouting, and redox reduction remains open; in fact, as correctly concluded by the expert, the experiments measuring carbonyls suggest that this may not be the case.
Indeed, the main message of supplemental figure 4a (now 6A in the revised manuscript) is that no oxidation can be detected in *Ercc1* mutants. While these are very interesting research questions, an in-depth investigation of the reported phenomena in natural aging falls outside the scope of this study.

Major concerns (experimentally)

- The authors focus on RNA polymerase II transcription to support their claims. Yet, Pol I transcription accounts for the higher proportion of the cell bulk transcription (as deduced when noticing its efficient shut off after dietary restriction or rapamycin treatment), and is the most suited machine to promote significant alterations susceptible of effectively “saving resources”, as is the scope of this work. Moreover, the central NER player TFIIH is now formally demonstrated as a relevant player in promoting efficient RNA polymerase I transcription elongation, as revealed by the work from the lab of G. Giglia-Mari and others. Thus, in the context of this work, the impact, extent and importance contributed by RNA polymerase I transcription should be assessed. For example, in Figure 1A and more even in Figure 1B, the strong, remaining EU signals are very much reminiscent of nucleoli. Are they so? Can the authors simultaneously hybridize for a nucleoli marker and split their EU signals into those contributed by nucleoli transcription (mostly Pol I-dependent) and the rest? Would this add something new to their conclusions? Second, can the authors envision a way to (at least to a great extent) inhibit specifically RNA Pol I and assess some of their metabolic readouts?

As per request of the reviewer, in the revised version of the manuscript we have performed additional measures monitoring the EU signal in and outside the nucleolus (please see supplementary figure 1), also mentioning a possible involvement of RNA pol I and including the reference suggested by the expert (please see line 11 and below in the first paragraph of the results section entitled *Defective NER causes progressive decline of transcription*). The data show that the decline in EU signal is comparable in the two sub-compartments, in *Ercc1* mutants versus wild-type controls.
Concerning the second point raised by the reviewer, it is true that rRNA transcription constitutes approximately 60% of the total transcription output (Grummt 2003), However, these figures are for proliferating cells, which have to synthesize a new cellular content of ribosomes. Values for non-proliferating cells are lower as ribosomal RNA is very stable. Nevertheless, it is conceivable that inhibition of RNA pol I may lead to ATP imbalance as well. We have added these considerations to the discussion (please see end of the first section of the discussion). That said, we respectfully suggest that dissecting the transcriptional dynamics at such granular level does not add much to the fundamental message of this study – i.e. that increased ATP levels caused by general transcription decline cause glucose rerouting and reduction of the redox environment - and is rather an excellent topic for a follow up study.

- In referring to Figure 1B, the authors sharply claim that the heterogeneity seen in EU incorporation is a proof of the stochasticity of the DNA damage-triggered transcription halt. Other options are possible and therefore this should just be put as an option, a suggestion. Yet, there is one very straightforward one, that is, that the cells may be markedly in different phases of the cell cycle because of the ercc1 deficiency, then bearing different transcriptional activities. A cytometry measurement here, if unchanged, would help the authors suggest (yet not claim), their purpose.

As discussed above, we toned down our phrasing on DNA damage accumulation (please see end of the first paragraph of the results section entitled *Defective NER causes progressive decline of transcription*). Additionally, in the revised version we present also the EU data *in vivo* as scattered dot plots; this representation illustrates that

heterogeneity is high also in tissue, especially at 4 weeks of age (fig. 1A). This evidence suggests that heterogeneity cannot be attributed to cell cycle because hepatocytes *in vivo* are largely non-dividing and enter cell cycle predominantly after injury (Miyaoka, Ebato et al. 2012).

- One main claim of the paper is that the metabolic remodelling tethers an increase in the levels of NADPH. While this is central to support one of the main paper messages, the measurements in search of increased NADPH were performed only in two conditions (ercc1 and xpg mutants), and only found altered as expected in the first case. This part needs therefore to be reinforced. I would suggest repeating the measurements when inhibiting transcription pharmacologically for a short time, for example. In this same line, it is also very striking that, while the transcription decline in the xpg mutant is dramatic, much stronger than in the ercc1 animals, still the NADPH measurement does not fulfil the predictions. I do not think this invalidates the main notion, but I think that the authors should find an experimental approach to try to reconcile this apparent paradox. Is the NADPH being redirected to something else in the xpg mouse that prevents us from seeing it?

The reviewer raises a very interesting question. We would like to point out, however, that NAPDH is used as an electron donor in a plethora of biochemical pathways. Determining the reasons underlying the differences in NADPH redox state observed in *Ercc1* and *Xpg* mutants would require extensive metabolomics analyses eventually followed by investigations at protein and transcript level. These experiments are not trivial and would require a dedicated in-depth study. In addition they would involve setting up ageing cohorts for both *Ercc1* and *Xpg* mouse mutants, which need time-consuming, laborious (and costly) approval of the animal ethical committee; moreover, after positive feedback at least 6 additional months will be necessary before mice will be available for analysis. This is unfortunately beyond the scope of this manuscript.

- In the same line, the authors do not find a significant increase in G6PD activity when trying to induce metabolism remodelling using externally provided A. Why would this

be? Can the authors try a way / a condition to prove this point? Would it be possible to overcome this “negative result” by scoring an alternative readout, for example, that the PPP products are indeed exacerbated?

G6PD activity may be unaltered in this experimental setting for multiple reasons; the reviewer will certainly agree that metabolic networks are intricate and extremely plastic, and several buffering and compensatory mechanisms can be put in place to preserve homeostasis. As previously stated, a comprehensive analysis would require extensive metabolomics approaches followed by analyses at proteins and gene level, which we believe would not add much to the core message of this study.

Assessing PPP intermediates as suggested by the reviewer would require flux analysis, which in this specific case would be extremely challenging given that cells should be cultured in the presence of adenosine and isotopic glucose (i.e. the tracer), which influences adenosine uptake- see e.g. (Leung, Man et al. 2005; Pastor-Anglada and Perez-Torras 2018). These conditions do not affect the comparison between two genotypes because under these circumstances adenosine and isotopic glucose are present in equal amounts in the experiment and control groups; comparisons between groups in which the concentration of the chemicals vary, however, will likely lead to a very unreliable outcome.

We believe that, together with the other analyses in the study, the experiment using adenosine to manipulate ATP levels and cause PFK inhibition provide convergent evidence supporting the fundamental focus of the study, i.e. that increased levels of ATP caused by transcription block can allosterically inhibit glycolysis and favor glucose rerouting.

- The model of the authors predicts that a hyper-active, boosted transcription would deplete ATP and lead to a situation where an oxidative redox status would predominate. This is in agreement with our notion of, for example, cancer cells growth. While not being critical, if a system (even a non-mouse system) could be used to suddenly boost transcription and evaluate the key parameters the authors measure, this would beautifully reinforce their model.

Because it is ascertained that ATP depletion causes necrotic cell death, the cell developed systems to maintain ATP homeostasis under intense transcriptional activity. For instance, increased transcription following estrogen treatment is accompanied by bioenergetics potentiation and augmented mitochondrial respiration in particular – see e.g. (Yao, Chen et al. 2011). Under some circumstances, for instance cancer growth, increased mitochondrial respiration could be associated with increased ROS production as pointed out by the reviewer. These mechanisms, however do not necessarily rely on the pathways we described, which mainly concern post-mitotic, non-proliferating conditions, in which transcription stress can build up over time and replication does not resolve arrested transcription complexes.

- The notion that derives from the paper that a too reduced environment is deleterious for the cell is of high relevance. We tend to read and hear that a highly oxidative environment is harmful. We are less used to the opposite notion. On these grounds, it would be better for the impact and the consideration of the paper to probe for a molecular process being negatively affected by the reducing environment. For example, I imagine that protein folding within the Endoplasmic Reticulum would enormously suffer from this. In this case, the cells will activate the Unfolded protein response (UPR). There is a high degree of correlation between genome instability and ER stress, yet little molecular connexions. If the authors manage to validate a transcriptional activation of the UPR and/or an expansion of the ER folding compartment by microscopy, for example, not only will they reinforce the physiological consequences of the altered redox state but also will provide a connexion for these, otherwise, only correlated events in the bibliography.

This very interesting question does not have a straightforward answer. The reviewer hypothesizes that an alteration in the cytosolic redox environment should exert consequences at the level of protein folding in the ER. The intracellular redox state, however, is heavily compartmentalized (Kaludercic, Deshwal et al. 2014) and alterations in one location do not necessarily imply alterations in other locations. The research problem

posed by the reviewer can therefore only be pursued via imaging methods based on organelle-specific redox sensors (e.g. ro-GFP). We honestly believe that this approach falls beyond the scope of the present study.

However, as per request of the reviewer, we have measured transcriptional variations in genes of the UPR pathway and found several differently regulated genes (please see table below). At present, however, we cannot attribute these changes to altered redox state and addressing this topic requires dedicated, in depth investigations. For this reason, we would suggest to leave this piece of information out of the manuscript given that it does not add substantial information in favor of the proposed model, also in consideration of the data in figure 4J showing increased resistance of *Ercc1* mutants to the pro-oxidant diamide, which largely exert its toxic effect mediating cysteine oxidation.

Gene Symbol	logFC	Adjusted P Value	Annotated Term
Derl2	-0.74	0.0008	endoplasmic reticulum unfolded protein response
Vapb	-0.63	0.0162	endoplasmic reticulum unfolded protein response
Ern1	-0.62	0.0132	endoplasmic reticulum unfolded protein response
Dnajc3	-0.61	0.006	endoplasmic reticulum unfolded protein response
Parp16	0.7	0.0036	endoplasmic reticulum unfolded protein response
Bax	0.89	0	positive regulation of endoplasmic reticulum unfolded protein response
Herpud2	1.35	0.0004	endoplasmic reticulum unfolded protein response
Nfe2l2	1.52	0.004	endoplasmic reticulum unfolded protein response
Ccnd1	2.02	0.0161	endoplasmic reticulum unfolded protein response

Minor concerns (conceptually)

- At a point in the Discussion the authors write “a key role of the PPP in the DNA damage response”. The DNA Damage Response, the DDR, responds to a well-established term to designate the cascade of sensing, transmitting and reacting to DNA lesions in the DNA, which is orchestrated by apical kinases such as ATM and ATR, and effectors such as Chk2 and Chk1. Given the existence of such a network, with which the present work does not cope, I suggest to simply change this and other similar sentences of the paper by “the response to DNA damage”. It is very minor change but, conceptually, it changes a lot.

We thank the referee for this suggestion, which has been followed up.

- All the oxygen consumption rates curves (as in Figure 2E, for example), the drugs used at each step, why, what they do, what this evaluates, etc, please explain widely and with details in the main Results text, it is greatly under-explained even for experts, therefore imagine for those who are not.

We have now added further details in the Results section; however, we suggest providing an in-depth explanation in the figure legends, not to impact readability of the Results' section.

- The event of polyploidization is not apparent or even understood for the non-specialist. A quantification (if possible), a better visual representation and a better explanation would be appreciated.

The presence and the quantitation of polyploidy nuclei in *Ercc1* mutants have been described by our laboratories (please see the reference by Weeda et al., 1997 included in the original version) and others (please see (Nunez, Chipchase et al. 2000; Chipchase, O'Neill et al. 2003), now included in the revised text).

- The affirmation that "protein carbonyls are a footprint of RS" needs to be justified by a reference.

We added a reference (Stadtman and Levine 2000) that substantiates this claim, also in light of the limited capacity to repair protein damage that is largely confined to the reduction of oxidized sulfur-contain amino acids and contrasts with the effective repair of oxidative lesions in DNA by base excision and insertion mechanisms.

- There is a small, yet important raise in the number of works assessing the reciprocal impact of metabolic alterations and genome status. It is important to give visibility to these works, for they constitute the basis for a more open attitude towards this re-

search. The authors make reference to the work by Vermeij et al, 2016. The paragraph in the discussion “DNA damage and glucose metabolism” would be a good place to enlarge the view about this issue. I encourage the authors to make a more extensive search and dissertation around this theme. For example the short Mol. Cell by Brian Luke’s lab discussing genome fidelity maintenance and dietary restriction-like treatments would be a good starting point.

We thank the reviewer for this suggestion. However, we are afraid we could not identify the indicated article from Brian Luke’ s lab in Molecular Cell discussing genome fidelity maintenance and dietary restriction-like treatments. We identified a Molecular Cell paper in which Dr. Lukes figures as first author that focuses on Rat1p 5' to 3' exonuclease and telomere elongation in *Saccharomyces cerevisiae* (Luke, Panza et al. 2008), therefore dealing with a topic that is rather distant from the subject of our manuscript. Another paper published in Cell Reports from Dr. Luke’s laboratory explores the effects of TORC1 inhibition on checkpoint adaptation in dividing yeast (Klermund, Bender et al. 2014). Given that hepatocytes *in vivo* are quiescent and have a very slow turnover estimated in 200-300 days (please see (Bucher and Malt 1971)), it is hard to envision how the results in dividing yeast describes in this Cell Reports paper can be translated to our system.

That said, if the reviewer will indicate the specific articles to discuss, we would be more than happy to elaborate on these works in our manuscript.

- The authors discuss briefly that the increased activation of G6PD can be mediated by the DNA damage response protein ATM. This observation, which matches so beautifully with their observation, deserves, in my view, a bit more development. How does the kinetics of this phenomenon compare to the one the authors describe here? We have the general idea that the transcriptional response is fast, and that the DNA damage response mediated by proteins such as ATM is even faster. But the allosteric regulation proposed in this work would be even “more” faster. Are these timings compatible with what is described?

As mechanism of action, we anticipate that allosteric modulation will work as fast, if not faster than ATM mediated regulation of G6PD, which relies on protein-protein interactions. Obviously, careful biochemistry experiments - which we believe fall outside the scope of this work - need to be designed to address issue. That said, when contextualized longitudinally, in the living organism, the transcription stress initiating metabolic redesign via increased ATP levels builds up in time, as disclosed in our manuscript, specifically in ageing post-mitotic organs and tissues. This may also cause gradual, chronic ATM activation, which can in turn target G6PD. The kinetics of this activation is likely to be very slow as the process as a whole occurs during (accelerated) ageing over months.

- Measurement of glycolysis as indicated by medium acidification requires using a culturing medium that IS NOT BUFFERED (Mekhail Lee 2006 Cell Cycle). Yet, authors claim in the methods section that they used a medium containing 100 mM sodium pyruvate at pH 7. Is this perhaps relevant to the accuracy of the measurements?

Indeed, the medium in the Seahorse experiments is not buffered. Otherwise, it would not be possible to detect ECAR changes. We apologize for the confusion; the use of unbuffered medium has now been clearly stated (please see third line in the methods section entitled Bioenergetics Assays).

- It is not intuitive to understand why the short genes would be UPREGULATED in the ercc1 mutants. Since the reason why the ercc1 mutants display decreased general transcription is not so critical for the message of the paper, could this transcriptome analysis be given less importance in the main paper flow?

As the *Ercc1* expression profile is biased towards small genes, the relative proportion of small genes in the total transcriptional output is increased compared to wt controls. However, the referee is correct: in absolute terms there is no ‘upregulation’. That is why the sentence in the results section is phrased “*Indeed, the transcriptome of Ercc1*$^{\Delta/-}$ *liv-*

ers (16-weeks old) was relatively enriched in transcripts from short genes and significantly depleted in transcripts of long genes (fig.1C)" (please see line 7, page7).

Minor concerns (experimentally)

- Quantification of EU in Figure 1A is not satisfactory. Authors do not use the same representation than in Figure 1B, which is much fairer, for raw EU intensity (even in arbitrary units) is provided. The way shown in Figure 1A prevents us from seeing the actual evolution of transcription activity in the WT, and does not allow us to see the individual values for different cells of the population. This is a loss, for perhaps two populations may be apparent, one more affected than other. I propose to display one representative experiment of the actual population of cells with all the raw values. To see the reproducibility of the experiment, other experiments can be provided with the same format in Supplementary.

EU quantification in figure 1A is now represented as in 1B, as a scattered dot plot.

- Given the strong heterogeneity observed in EU incorporation in the experiment presented in Figure 1B and Sup Fig 1, one would expect that the quantification yields two population of points, which is not the case. Is there a mistake in this quantification? And in the same line as above, could authors provide the same quantification for the biological replicates of this experiment in supplementary figures?

It is not given that heterogeneity in vivo occurs to the same extent in an in vitro experimental setting. Please note that the principal objective of this experiment is to demonstrate that differences in vivo are not due to different penetration of EU into the tissue.

- How sure are we that the used drugs (i.e. transcription inhibitors) do not affect mitochondrial performance? Can the authors comment on this?

As indicated by the OCR trace in figure 2A and in supplementary figure 3A, direct injection of transcription inhibitors does not induce any immediate changes in respiration;

this is in sharp contrast with chemicals with ascertained and direct effects on mitochondrial activity – e.g. oligomycin, FCCP, rotenone, and antimycin – which exert an prompt effect on respiration (see e.g. the trace in figure 2E). Obviously, in the long run transcription inhibitors will affect the organelles given that the very vast majority of mitochondrial proteins is coded by nuclear DNA.

- The authors claim “genes of key glycolytic enzymes are very compact, thus relatively unaffected by the DNA damage-induced decline”: this is not shown by any analysis.

We have removed this statement from the revised version of the manuscript.

- The panels from Supp Figure 1 would be appreciated already in the main Figure 1, to have a better overview of the cell population.
As per request of the reviewer, the panel has been moved from supplementary figure 1 and has been integrated in figure 1B.

- The WB for GSH level in Figure 4H needs to be shown!

We apologize for the lack of clarity; GSH was measured using a well-established spectrophotometric assay. In the revised version of the manuscript, this has been explained in greater detail in both the methods (please see the section entitled *Glutathione measurement*) and in the figure legend.

- The survival curves in Figure 4L need to be much bigger.

We expanded to x-axis of the curve to improve readability, thanking the referee for noticing.

- The y-axes and the titles in Figure 3E cannot be read.

This has been fixed as well.

- The amount of carbonylated proteins in Ercc1 mutants at 16 weeks is decreased compared to the WT, yet the difference is not significant. I get it from the Figure that the experiment was done only twice? Could this be reinforced?

The experiment has been performed on two animals, in three independent technical replicates. While the decrease in carbonyls is not significant in *Ercc1* mutants, the experiment clearly shows – in line with the other convergent data provided in the study – that there is no excessive oxidation in mutant mice.

I congratulate the authors for this interesting, necessary and inspiring work and encourage them with the eventual revision of the manuscript, for this will be a beautiful contribution to science.

We greatly appreciate the expert encouraging feedback; we are flattered.

References used in the rebuttal

Andrade-Lima, L. C., A. Veloso, et al. (2015). "DNA repair and recovery of RNA synthesis following exposure to ultraviolet light are delayed in long genes." Nucleic Acids Res **43**(5): 2744-2756.

Bucher, N. L. R. and R. A. Malt (1971). Regeneration of liver and kidney, Little, Brown.

Cellerino, A. and A. Ori (2017). "What have we learned on aging from omics studies?" Semin Cell Dev Biol **70**: 177-189.

Chipchase, M. D., M. O'Neill, et al. (2003). "Characterization of premature liver polyploidy in DNA repair (Ercc1)-deficient mice." Hepatology **38**(4): 958-966.

Clark, J., E. L. Clore, et al. (2010). "Oral N-acetyl-cysteine attenuates loss of dopaminergic terminals in alpha-synuclein overexpressing mice." PLoS One **5**(8): e12333.

Giglia-Mari, G., C. Miquel, et al. (2006). "Dynamic interaction of TTDA with TFIIH is stabilized by nucleotide excision repair in living cells." PLoS Biol **4**(6): e156.

Giglia-Mari, G., A. F. Theil, et al. (2009). "Differentiation driven changes in the dynamic organization of Basal transcription initiation." PLoS Biol **7**(10): e1000220.

Greenamyre, J. T., J. R. Cannon, et al. (2010). "Lessons from the rotenone model of Parkinson's disease." Trends in pharmacological sciences **31**(4): 141-142; author reply 142-143.

Grummt, I. (2003). "Life on a planet of its own: regulation of RNA polymerase I transcription in the nucleolus." Genes Dev **17**(14): 1691-1702.

Hanasoge, S. and M. Ljungman (2007). "H2AX phosphorylation after UV irradiation is triggered by DNA repair intermediates and is mediated by the ATR kinase." Carcinogenesis **28**(11): 2298-2304.

Hoogstraten, D., A. L. Nigg, et al. (2002). "Rapid switching of TFIIH between RNA polymerase I and II transcription and DNA repair in vivo." Mol Cell **10**(5): 1163-1174.

Kaludercic, N., S. Deshwal, et al. (2014). "Reactive oxygen species and redox compartmentalization." Front Physiol **5**: 285.

Kemp, M., Y. M. Go, et al. (2008). "Nonequilibrium thermodynamics of thiol/disulfide redox systems: a perspective on redox systems biology." Free Radic Biol Med **44**(6): 921-937.

Kimura, H., Y. Tao, et al. (1999). "Quantitation of RNA polymerase II and its transcription factors in an HeLa cell: little soluble holoenzyme but significant amounts of polymerases attached to the nuclear substructure." Mol Cell Biol **19**(8): 5383-5392.

Klermund, J., K. Bender, et al. (2014). "High nutrient levels and TORC1 activity reduce cell viability following prolonged telomere dysfunction and cell cycle arrest." Cell Rep **9**(1): 324-335.

Leung, G. P., R. Y. Man, et al. (2005). "D-Glucose upregulates adenosine transport in cultured human aortic smooth muscle cells." Am J Physiol Heart Circ Physiol **288**(6): H2756-2762.

Luke, B., A. Panza, et al. (2008). "The Rat1p 5' to 3' exonuclease degrades telomeric repeat-containing RNA and promotes telomere elongation in Saccharomyces cerevisiae." Mol Cell **32**(4): 465-477.

Miyaoka, Y., K. Ebato, et al. (2012). "Hypertrophy and unconventional cell division of hepatocytes underlie liver regeneration." Curr Biol **22**(13): 1166-1175.

Nunez, F., M. D. Chipchase, et al. (2000). "Nucleotide excision repair gene (ERCC1) deficiency causes G(2) arrest in hepatocytes and a reduction in liver binucleation: the role of p53 and p21." FASEB J **14**(9): 1073-1082.

Pan-Montojo, F., O. Anichtchik, et al. (2010). "Progression of Parkinson's disease pathology is reproduced by intragastric administration of rotenone in mice." PLoS One **5**(1): e8762.

Pastor-Anglada, M. and S. Perez-Torras (2018). "Who Is Who in Adenosine Transport." Front Pharmacol **9**: 627.

Pollak, N., C. Dölle, et al. (2007). "The power to reduce: pyridine nucleotides – small molecules with a multitude of functions." Biochemical Journal **402**(2): 205.

Reyes, R. C., G. F. Cittolin-Santos, et al. (2016). "Neuronal Glutathione Content and Antioxidant Capacity can be Normalized In Situ by N-acetyl Cysteine Concentrations Attained in Human Cerebrospinal Fluid." Neurotherapeutics **13**(1): 217-225.

Schumacher, B., I. van der Pluijm, et al. (2008). "Delayed and accelerated aging share common longevity assurance mechanisms." PLoS Genetics **4**(8): e1000161.

Stadtman, E. R. and R. L. Levine (2000). "Protein oxidation." Ann N Y Acad Sci **899**: 191-208.

Sun, F., C. Dai, et al. (2012). "Biochemical issues in estimation of cytosolic free NAD/NADH ratio." PLoS One **7**(5): e34525.

Vermeij, W. P., M. E. Dolle, et al. (2016). "Restricted diet delays accelerated ageing and genomic stress in DNA-repair-deficient mice." Nature **537**(7620): 427-431.

Wang, J., C. L. Clauson, et al. (2012). "The oxidative DNA lesions 8,5'-cyclopurines accumulate with aging in a tissue-specific manner." Aging Cell **11**(4): 714-716.

Wei, Y. N., H. Y. Hu, et al. (2015). "Transcript and protein expression decoupling reveals RNA binding proteins and miRNAs as potential modulators of human aging." Genome Biol **16**: 41.

Yao, J., S. Chen, et al. (2011). "Estrogen protection against mitochondrial toxin-induced cell death in hippocampal neurons: antagonism by progesterone." Brain Res **1379**: 2-10.

REVIEWERS' COMMENTS:

Reviewer #1 (Remarks to the Author):

In this revised version of the Milanese et al. manuscript, the authors describe how the presence of transcription-blocking lesions induces a DNA damage dependent rewiring of cellular metabolic pathways. This occurs because ATP is now accumulated in cells as not used by an energetically expensive process such as transcription, and ATP acts as allosteric inhibitor enzymes in the pathway. The originally submitted version had expanded on previously published data about how changes in cellular metabolic pathways were observed in contexts with defective nucleotide excision repair. In this revised version of the paper, the authors address most of the comments that reviewers had, and have added many experimental evidences to support their initial finding. These added experiments have also led to a toning down some of the statements and conclusions, making them more in line with the actual data supporting their findings. In particular, that now "these arguments strongly favour time-dependent accumulation of DNA damage as the principal cause of the transcription stress".
In parallel, the overall quality of the paper has been improved, with greater experimental details provided in the figure legends and in the material and methods section, that are now solid and robust. All in all, I believe that this manuscript will be relevant for the broad audience of Nature Communications readers, as the findings presented here can have implications for many other disease-related contexts, and I am in favour of its publication.

Reviewer #2 (Remarks to the Author):

The changes made to the manuscript by Mastroberardino and colleagues have strengthened it. The submission makes a substantial novel contributions to the areas of DNA repair and metabolism, and will be of substantial interest to the readership of Nature Communications. I support publication, provided that the authors can address 3 small concerns:

1. On the prior submission, I recommended that the authors change the (many!) bar graphs in the paper to scatterplots, in the interest of transparency. Many journals (e.g. JBC) require this as a matter of course. Their claims to the contrary notwithstanding, as far as I can tell the authors have retained all of their bar graphs, and not converted any.

2. Although the authors show ECAR data in the manuscript in several contexts, they actually never show it for the Ercc1 mutant, which is a core focus of the work, though summary bar graphs are shown. I think the authors should show primary ECAR data for the mutant, to complement the summary data.

3.There are a few typos and misspellings throughout (e.g. "reumathoid arthritis").

Reviewer #3 (Remarks to the Author):

Milanese, Bombardieri and collaborators have provided the revised version of their manuscript, under consideration for publication in Nature Communications. To evaluate with the fairest possible eye their task, I have read and considered their whole activity, which therefore includes the answers to the concerns raised by other two reviewers, not only to mine.

The concerns raised by the three reviewers were, conceptually, not very far from each other, evidencing key points that needed to be assessed, notably: 1) the causative role of DNA damage as the source of transcription blockage was not demonstrated (while plausible), 2) the information

provided by the figure legends, the clarity of axes, the choice of quantification plots or even the absence of panels needed to be revised in-depth, 3) the danger of having off-target effects by the drugs and inhibitors used was not sufficiently considered and/or explained.

On the contrary, the experimental concerns raised by the reviewers were more dissimilar and requested from the authors a very wide palette of novel experiments, each promoted by the reviewer's own field of expertise and / or interests. For example, the authors have been challenged with proposals /exigences ranging from RNA polymerase II characterization or assessment of bulk translation rates to exploration of RNA polymerase I implication and microscopy cell imaging of the Endoplasmic Reticulum status. This makes the answer to all these requests inviable and, as the authors indicate, beyond of the scope of the work: the exploration could be launched on five or six novel fields, but this is in fact future research. This is why I understand and do not penalize that many of the concerns have been addressed by the simple (but elaborated) means of the argumentation and the discussion.

Yet, I humbly think that, in this task, the authors have exceeded the limits. There are things that were requested that could have been done and some that were even stated as an exigence, yet the authors have "replied" with the word, not with the experiment. The case I consider the more extreme does not belong to my requests but to one of Reviewer 1. He / she wrote: "the authors should prove that the reduced progressive transcription output is dependent on increasing DNA damage in the cells (gH2AX foci for example)….". This particular request is formulated on the grounds of an exigence and represented an opportunity for providing the experimental support for one of the most critical criticisms raised by the three of us. The authors engage into an explanation to justify why gH2AX is not a good choice, and do not provide any experimental support. The reviewer had yet proposed gH2AX as an example. If the authors had their reasons not to consider it appropriate, they could have envisioned other means, ie CPDs, or yet something else.

On my side, I only have minor concerns:

- I had raised the relevance of considering the contribution of RNA polymerase I to the phenomenon they characterize. I proposed to quantify EU signals coming from nucleoli and to specifically inhibit RNA polymerase I transcription to trigger ATP raise. This latter request has not been assessed experimentally, and I can accept they decided to skip this. As for the first request, the authors have found a useful image-based way of estimating the transcription happening in nucleoli without having to engage into co-staining of samples with nucleoli markers, which allowed them to exploit their original data. They provide this data in Supplementary Figure 1. To my surprise, they provide values for 18 nucleoli and 10 nuclei. In this type of experiment, at least 100 objects are normally evaluated, more if we want to be stringent. It is not because the trend is already clear and the p-values highly significant that we stop counting. I am not implying at all that the authors chose the 20 objects of their convenience, but from a statistical point of view this is not ideal, especially when considering that the authors may have thousands of nucleoli at their disposition within their images.
- For Figure 1A, I requested dot plots of one experiment, in order to see the whole population data and to make it comparable to the information displayed in Figure 1B, which has been done. Additionally, I indicated that, by normalizing the ercc1 data to the WT (expressed as 100%), the information relating the evolution in time of WT EU signals was lost. In this revised version, the authors still plot the ercc1 data as a percentage of the normalized WT. I imagine this decision of the authors is prompted by the apparent increase in EU signal in WT at 20 weeks, which is difficult to understand, but still this raw information should not be hidden, perhaps by placing it in Supplementary.
- Figure legend of Supplementary Figure 6 has evidently not been revised. We still cannot know which the difference between panels A and B is (if any, if they are replicates of the experiment, then specify), and what are numbers from 1 to 6.
- In the main text, first results section, when authors introduce their intention to study ubiquitination of RNA Pol II, please explain what this means. Ubiquitination is not only a modification for destruction,

the reader needs to be introduced to the logic of the experiment.
- The body of the idea posited by the authors is that the increase in ATP will trigger an allosteric modulation of some metabolic enzymes. A conceptual hinder (to me) of the way data are presented and discussed is how this ATP raise will impact and modulate, downstream, the expression of genes, for example when they claim this for glutamate-L-cysteine ligase (Fig. 4F). Could this notion be considered (even if only by speculation)?
- First sentence of page 16, the authors write "one apparent aim of rerouting metabolism from…". Please, reformulate. There is no intentionality in the cell's activities…
- Perhaps I missed it, for I have not read the paper, only checked the abstract, but I think that reference 60, used to justify DRR sensitivity (beginning of page 17) is not correct. The correct one may have escaped.
- Concerning this same sentence, the authors want to reinforce their notion that a highly reduced state accompanies DNA repair defects by claiming that fibroblasts from patients with severe DNA repair defects have been reported to be sensitive to the reducing agent DTT. Yet, this could be due to really unrelated things. For example, DTT is a strong stressor of the Endoplasmic Reticulum, for it prevents correct protein folding. The reason why DNA repair-deficient cells are DTT-sensitive could be that they tolerate badly to be confronted to both genotoxic and proteotoxic stresses. Yet, the findings of the authors could indeed provide an initial hint on our ignorance concerning the fact that ER stress and genotoxic stress are extensively correlated in the literature, although whether this is functionally related or just a correlation remains unknown. Their sentence could be re-phrased considering these notions.

To conclude, in my general view, the paper is a very comprehensive work, with a clear message, irrespective of the many concerns each of us could have raised, which holds and is trustworthy. The main body of evidence thus is supported and nothing of what I have written as concerns (which yet should be implemented) should prevent, from my side, the publication of the work. I congratulate the authors once more, and sincerely hope my contribution helped them.

Response to the reviewers

We thank once more the reviewers for the very insightful feedback.

The original reviewers' comments are in blue font.

In the main text, the changes are highlighted in green.

Reviewer #2 (Remarks to the Author):

The changes made to the manuscript by Mastroberardino and colleagues have strengthened it. The submission makes a substantial novel contributions to the areas of DNA repair and metabolism, and will be of substantial interest to the readership of Nature Communications. I support publication, provided that the authors can address 3 small concerns:

We sincerely thank the reviewer for the support and for the insightful, knowledgeable feedback.

1. On the prior submission, I recommended that the authors change the (many!) bar graphs in the paper to scatterplots, in the interest of transparency. Many journals (e.g. JBC) require this as a matter of course. Their claims to the contrary notwithstanding, as far as I can tell the authors have retained all of their bar graphs, and not converted any.

As per request of the reviewer, we now represent the graphs in scatterplot format. To improve readability, we superimposed the scatter dot plots to the bar graph, following a format used in several publication in Nature Communications (see e.g. https://www.nature.com/articles/s41467-019-11855-w; https://www.nature.com/articles/s41467-019-11843-0; https://www.nature.com/articles/s41467-019-11799-1).

2. Although the authors show ECAR data in the manuscript in several contexts, they actually never show it for the Ercc1 mutant, which is a core focus of the work, though summary bar graphs are shown. I think the authors should show primary ECAR data for the mutant, to complement the summary data.

This has been added in figure 2.

3.There are a few typos and misspellings throughout (e.g. "reumathoid arthritis").

We further double-checked for typos and misspellings.

Reviewer #3 (Remarks to the Author):

Milanese, Bombardieri and collaborators have provided the revised version of their manuscript, under consideration for publication in Nature Communications. To evaluate with the fairest possible eye their task, I have read and considered their whole activity, which therefore includes the answers to the concerns raised by other two reviewers, not only to mine.

We thanks once again the reviewer for the careful, comprehensive, and meticulous assessment of our work.

On my side, I only have minor concerns:

- I had raised the relevance of considering the contribution of RNA polymerase I to the phenomenon they characterize. I proposed to quantify EU signals coming from nucleoli and to specifically inhibit RNA polymerase I transcription to trigger ATP raise. This latter request has not been assessed experimentally, and I can accept they decided to skip this. As for the first request, the authors have found a useful image-based way of estimating the transcription happening in nucleoli without having to engage into co-staining of samples with nucleoli markers, which allowed them to exploit their original data. They provide this data in Supplementary Figure 1. To my surprise, they provide values for 18 nucleoli and 10 nuclei. In this type of experiment, at least 100 objects are normally evaluated, more if we want to be stringent. It is not because the trend is already clear and the p-values highly significant that we stop counting. I am not implying at all that the authors chose the 20 objects of their convenience, but from a statistical point of view this is not ideal, especially when considering that the authors may have thousands of nucleoli at their disposition within their images.

We have now extended these analyses include more object (n≥164 for nucleoli, n≥111 for nuclei - see supplemental figure 1B, C, and also Source Data File).

- For Figure 1A, I requested dot plots of one experiment, in order to see the whole population data and to make it comparable to the information displayed in Figure 1B, which has been done. Additionally, I indicated that, by normalizing the ercc1 data to the WT (expressed as 100%), the information relating the evolution in time of WT EU signals was lost. In this revised version, the authors still plot the ercc1 data as a percentage of the normalized WT. I imagine this decision of the authors is prompted by the apparent increase in EU signal in WT at 20 weeks, which is difficult to understand, but still this raw information should not be hidden, perhaps by placing it in Supplementary.

As per request of the reviewer, we have now plotted all EU data as absolute values.

- Figure legend of Supplementary Figure 6 has evidently not been revised. We still cannot know which the difference between panels A and B is (if any, if they are replicates of the experiment, then specify), and what are numbers from 1 to 6.

We apologize we overlooked this in the previous revision. Figure 6 legend has been now revised.

- In the main text, first results section, when authors introduce their intention to study ubiquitination of RNA Pol II, please explain what this means. Ubiquitination is not only a modification for destruction, the reader needs to be introduced to the logic of the experiment.

We have now amended the sentence, also adding new references, as follows "*We also explored whether transcription decline in Ercc1Δ/- is associated with increased ubiquitination of RNA Pol II, which – despite involvement of non-conventional signaling functions of ubiquitinylaiton in genome integrity maintenance*[21]*- has been shown to target the enzyme to degradation as a consequence of DNA damage*[22, 23]*.*"

- The body of the idea posited by the authors is that the increase in ATP will trigger an allosteric modulation of some metabolic enzymes. A conceptual hinder (to me) of the way data are presented and discussed is how this ATP raise will impact and modulate, downstream, the expression of genes, for example when they claim this for glutamate-L-cysteine ligase (Fig. 4F). Could this notion be considered (even if only by speculation)?

We thank the reviewer for this insightful consideration. We have now added the following sentences in the discussion "*Obviously, additional ATP based mechanisms independent from allosteric modulation may participate in the adaptive cellular response, possibly by reshaping the transcriptional landscape. In bacteria, for instance, it has been demonstrated that both high and low intracellular ATP concentrations affect macromolecular synthesis*[11, 51] *and that some promoters are directly activated by ATP*[11, 52]*. These mechanisms, however, have not been investigated in comparable detail in eukaryotic systems - at least to our best knowledge - and further studies will be necessary to elucidate how intracellular ATP concentration regulates the transcriptome.*"

- First sentence of page 16, the authors write "one apparent aim of rerouting metabolism from…". Please, reformulate. There is no intentionality in the cell's activities…

Done.

- Perhaps I missed it, for I have not read the paper, only checked the abstract, but I think that reference 60, used to justify DRR sensitivity (beginning of page 17) is not correct. The correct one may have escaped.

The reference is correct; please see figure 3 in the text showing increased sensitivity to DTT of specimens from patients harboring mutations in the polynucleotide kinase 3'-phosphatase gene, which has a key role in different DNA repair pathways.

- Concerning this same sentence, the authors want to reinforce their notion that a highly reduced state accompanies DNA repair defects by claiming that fibroblasts from patients with severe DNA repair defects have been reported to be sensitive to the reducing agent DTT. Yet, this could be due to really unrelated things. For example, DTT is a strong stressor of the Endoplasmic Reticulum, for it prevents correct protein folding. The reason why DNA repair-deficient cells are DTT-sensitive could be that they tolerate badly to be confronted to both genotoxic and proteotoxic stresses. Yet, the findings of the authors could indeed provide an initial hint on our ignorance concerning the fact that ER stress and genotoxic stress are extensively correlated in the literature, although whether this is

functionally related or just a correlation remains unknown. Their sentence could be re-phrased considering these notions.

We have now added the following sentence "…*while this feature could be caused by effects not directly related to reducing stress – for instance DTT mediated ER stress and consequent failure to properly fold proteins – it certainly establishes a nexus between reductive redox stress and defective DNA repair*"

To conclude, in my general view, the paper is a very comprehensive work, with a clear message, irrespective of the many concerns each of us could have raised, which holds and is trustworthy. The main body of evidence thus is supported and nothing of what I have written as concerns (which yet should be implemented) should prevent, from my side, the publication of the work. I congratulate the authors once more, and sincerely hope my contribution helped them.

We sincerely thank the reviewer for the insightful comments. We frankly believe that this expert knowledgeable comments greatly improved our work.
